# A behavioural dataset for studying individual differences in language skills

**DOI:** 10.1038/s41597-020-00758-x

**Published:** 2020-12-08

**Authors:** Florian Hintz, Marjolijn Dijkhuis, Vera van ‘t Hoff, James M. McQueen, Antje S. Meyer

**Affiliations:** 1 Max Planck Institute for Psycholinguistics, Nijmegen, The Netherlands; 2 Radboud University, Nijmegen, The Netherlands

**Keywords:** Human behaviour, Human behaviour

## Abstract

This resource contains data from 112 Dutch adults (18–29 years of age) who completed the Individual Differences in Language Skills test battery that included 33 behavioural tests assessing language skills and domain-general cognitive skills likely involved in language tasks. The battery included tests measuring linguistic experience (e.g. vocabulary size, prescriptive grammar knowledge), general cognitive skills (e.g. working memory, non-verbal intelligence) and linguistic processing skills (word production/comprehension, sentence production/comprehension). Testing was done in a lab-based setting resulting in high quality data due to tight monitoring of the experimental protocol and to the use of software and hardware that were optimized for behavioural testing. Each participant completed the battery twice (i.e., two test days of four hours each). We provide the raw data from all tests on both days as well as pre-processed data that were used to calculate various reliability measures (including internal consistency and test-retest reliability). We encourage other researchers to use this resource for conducting exploratory and/or targeted analyses of individual differences in language and general cognitive skills.

## Abstract (summary)

**Table:** 

Measurement(s)	Natural Language • cognitive function measurement
Technology Type(s)	Behavioral Assessment
Factor Type(s)	sex • age • education
Sample Characteristic - Organism	Homo sapiens
Sample Characteristic - Environment	laboratory environment

Machine-accessible metadata file describing the reported data: 10.6084/m9.figshare.13200365

## Background & Summary

Being able to produce and comprehend spoken utterances is what sets us apart from other species in the animal kingdom. Although we all have the ability to use language, we differ in that ability. And yet little is known about how we do so. The present dataset is a resource that can be used to study individual differences in language skills.

Since the advent of modern psycholinguistics, most experiments studying language have focused on describing the average behaviour of groups of individuals^1^. Consequently, most psychological models concern processing principles that are expected to apply to all language users.

In recent years, however, researchers have argued that psychological models should also explain the spread around the central tendency^2–5^. Thus, there has been growing interest in individual differences in language skills^6^. Using an individual-differences approach, previous studies have started exploring the relationships between different types of linguistic knowledge^3^ and the involvement of executive functions^7,8^ and working memory^9^ in language processing.

Compared to group-level experiments, however, the number of individual-differences studies is still very limited. There are important practical reasons for this. First, such studies naturally require large numbers of participants, thereby increasing testing time and participant fees. Second, the analyses – of language production data in particular – are labour-intensive, requiring hours of manual coding. Therefore, previous studies have often been confined to small sets of tests measuring specific skills of interest. Furthermore, while these studies provide a good starting point for investigating the cognitive architecture of the language system, interpretation is often difficult because potentially relevant variables were not assessed. For example, it has become clear that when conducting studies including language tests that require a speeded response, researchers should also assess participants’ non-verbal processing speed^10^.

The design of the present dataset addressed these concerns. We collected behavioural data from 112 native speakers of Dutch using our recently developed Individual Differences in Language Skills (IDLaS) test battery. The dataset allows researchers to obtain a comprehensive view on participants’ language skills and domain-general cognitive skills implicated in language. We make available data from three domains: (1) Linguistic experience, which is the knowledge acquired through an individual’s use of language (e.g., vocabulary, normative rules) and frequency of language exposure (e.g., reading frequency); (2) general cognitive skills, capturing variability in non-verbal skills that have been implicated in language processing (e.g., processing speed, working memory, non-verbal intelligence); and (3) linguistic processing skills, capturing variability in the four main tasks that individuals have to carry out when using language (i.e., word- and sentence-level processing in comprehension and in production). Following a latent-variable approach^11^, we included multiple tests per cognitive construct within each of these four domains.

The data were collected at the Max Planck Institute for Psycholinguistics in Nijmegen (NL) between March and July 2019 in the context of a large-scale project on individual differences in language processing and learning. Each participant completed the battery twice with approximately four weeks between test days. For each of the 33 tests, we make available the raw data output, including experiment logfiles and annotated speech recordings, from both test days. Moreover, we provide aggregated performance indicators for each participant, each test, and each test day. These were obtained by using a generic pre-processing pipeline. The pre-processed data were used for calculating measures of statistical reliability, including mean, standard deviation, range, skewness, kurtosis, internal consistency, and test-retest reliability.

This *Data descriptor* comprehensively describes the individual tests, the experimental procedures, the pre-processing protocol and the folder structure of the data, which are available at the UK Data Service data archive (UKDA). We encourage anyone interested to exploit this dataset for exploratory and/or targeted analyses. These analyses may focus on conceptual questions concerning, for example, the interactions between verbal and non-verbal cognitive systems. Analyses may also focus on addressing methodological questions concerning the stability (e.g. test-retest reliability) of the provided measures. As we make available both test scores aggregated by participants as well as the trial-level data, researchers may perform different types of analyses, such as (generalized) linear mixed-effects model analyses or structural equation modelling.

The dataset is owned by the Max Planck Institute for Psycholinguistics (Psychology of Language Department).

## Methods

### Participants

One-hundred and twelve participants were tested. Eighty-seven participants were students at or graduates from Radboud University or the HAN University of Applied Sciences (both in Nijmegen; 27 male, mean age: 22.6 years, range: 18 to 29 years); 24 attended or had attended a vocational college (12 male, mean age: 21.0 years, range: 18 to 29 years), and one participant was a high school graduate (female, 25 years). All participants were native speakers of Dutch. All participants gave written informed consent to take part in the study and were paid €92 for participation. In line with the informed consent, we used universally unique identifiers (UUIDs) as participant codes. Permission to conduct the study was provided by the Ethics Board of the Social Sciences Faculty of Radboud University (application number: ECSW-2019-19).

### Design and general procedure

Participants were tested in groups of up to eight individuals at the same time in a quiet room of about 30 m^2^ at the Max Planck Institute for Psycholinguistics. Each participant was seated at a desk with an experimental laptop, a computer mouse and a custom-made button box (two buttons) in front of them. Experimental laptops were Hewlett Packard ‘ProBooks 640G1’ with 14-inch screens, running Windows 7, optimized for experimentation. Participants were seated in a semicircle around the room facing the wall, with approximately 1 m – 1.5 m space between them. Noise cancelling divider walls (height 1.80 m, width 1 m) were placed between participant desks and the walls in front of them were covered with curtains to absorb as much noise as possible. Beyerdynamic DT790 headsets were used for the presentation of the auditory stimuli and to record participants’ speech. These headsets are commonly used in TV broadcasting and are known to shield environmental noise quite effectively. The headsets also come with high-quality close-to-mouth microphones. For the speech production tests, participants were additionally equipped with earplugs, which they wore underneath the headsets. This was done to ensure that participants’ own speech or speech planning was not influenced by overhearing other participants speak. Participants could still monitor their speech via bone conduction. Most participants indicated that they could not understand what other participants were saying and reported that the discomfort due to wearing earplugs and having to speak at the same time was minimal. Speech was recorded at a sampling rate of 44100 Hz, 16-bit resolution.

The tests were either implemented in Presentation© (version 20.0, www.neurobs.com) and run ‘locally’ on the laptops or were implemented as a web application in ‘Frinex’ (FRramework for INteractive EXperiments, an environment developed by the technical group at the Max Planck Institute for Psycholinguistics) and run online in the laptops’ web browser (Chrome, version 75.0.3770.142). Specifically, all tests where exact timing was critical (e.g., Reaction Time (RT) tests) were run in Presentation, while the rest was implemented in Frinex (see Tables 1 and 2 for an overview). As Frinex has been developed only recently, we did not have reliable data concerning its timing precision (i.e., time stamping of auditory, visual and response events).

**Table 1:** Overview of the order of tests in sessions 1 and 2, their sources, performance indicators and durations.

Session	Test	Source	Performance indicator	Duration
1 Frinex	0.1 Background questionnaire	New	—	10
	0.2 Short questionnaire on well-being	New	—	2
	1. Stairs4Words*	New	Prevalence band	10
	7. Syntest*	Janssen *et al*.^21^	Accuracy	5
	5. Idiom recognition test	Adapted from Hubers *et al*.^18^	Accuracy	3
	13. Digit span test (forward and backward)	Adapted from Wechsler^25^	Sum of correct trials (separate for forward and backward runs)	5
	17. Raven’s advanced progressive matrices test	Raven *et al*.^32^	Accuracy	25
2 Presentation	18. Picture naming test	Hintz *et al*.^10^	Mean onset latency	5
	29. Phrase and sentence generation	New	Mean speech duration (phrases) and accuracy (sentences)	15
	23. One-minute-test	Brus & Voeten^40^	Number of correctly pronounced words – number of incorrectly pronounced words in one minute	2
	24. Klepel test	Van den Bos *et al*.^41^	Number of correctly pronounced non-words – number of incorrectly pronounced non-words in one minute	3
	25. Monitoring in noise in lists	New	Proportion of correct responses to target-present trials – proportion of false alarms on target-absent trials (separate for each of the three monitoring tasks)	15
	15. Eriksen Flanker test	Eriksen^30^	Flanker effect (incongruent – congruent condition)	5
	32. Verb semantics activation during sentence comprehension	New	Mean RT predictable condition	8
	8. Auditory simple reaction time test	Hintz *et al*.^10^	Mean RT	3
	9. Auditory choice reaction time test	Hintz *et al*.^10^	Mean RT	5

Order was the same for all participants.

Note: Test number indexes in this table correspond to the order the tests are discussed in the text below, in the Supplementary Information and in the dataset itself. Data usage is discouraged for tests marked with an asterisk (see Usage Notes section for details).

**Table 2:** Overview of the order of tests in sessions 3 and 4, their sources, performance indicators and durations.

Session	Test	Source	Performance indicator	Duration
3 Presentation	33. Monitoring in noise in sentences	New	Difference between false-alarm corrected accuracy scores in predictable and non-predictable conditions	10
	16. Antisaccade test	Roberts *et al*.^31^	Accuracy	7
	31. Gender cue activation during sentence comprehension	New	Mean RT predictable condition	10
	27. Auditory lexical decision	Hintz *et al*.^10^	Mean RT words	7
	11. Visual simple reaction time test	Hintz *et al*.^10^	Mean RT	7
	12. Visual simple reaction time test	Hintz *et al*.^10^	Mean RT	
	26. Rhyme judgment	New	Mean RT rhyming trials	5
	10. Letter comparison test	Huettig & Janse^9^	Mean RT	5
	28. Semantic categorization	New	Mean RT category members	7
4 Frinex	21. Verbal fluency	Shao *et al*.^39^	Average number of produced words (separate for semantic categories and letters)	5
	19. Rapid automatized naming*	Araújo *et al*.^37^	Number of produced words per second	7
	20. Antonym production	Mainz *et al*.^38^	Accuracy	5
	30. Spontaneous speech	Jongman *et al*.^47^	-	5
	22. Maximal speech rate	New	Average speech duration	2
	14. Corsi block clicking test (forward and backward)	Chu *et al*.^27^	Sum of correct trials (separate for forward and backward runs)	7
	6. Prescriptive grammar	Adapted from Favier *et al*.^19^ Hubers *et al*.^20^	Accuracy	10
	2. Peabody picture vocabulary test	Dunn & Dunn^15^ Schlichting^16^	Percentile	10
	3. Spelling test	New	Proportion of correct responses to correctly-spelled words – proportion of incorrect responses to incorrectly-speed words	5
	4. Author recognition test	Brysbaert *et al*.^17^	Proportion of correct responses to authors – proportion of incorrect responses to foils	5

Order was the same for all participants.

Note: Test number indexes in this table correspond to the order the tests are discussed in the text below, in the Supplementary Information and in the dataset itself. Data usage is discouraged for tests marked with an asterisk (see Usage Notes section for details).

A test day started at 9.30 a.m., ended at 3.00 o’ clock p.m. and was divided into four sessions. Two sessions featured Presentation experiments and two sessions featured Frinex experiments. One session of each kind was run in the morning and one in the afternoon. Session length varied between 45 and 70 minutes depending on participants’ speed in carrying out the tests. Between sessions 1 and 2, and between sessions 3 and 4, participants were invited to take breaks of about 15–20 minutes. Between sessions 2 and 3, participants had a lunch break of 45 minutes. As is common practice in individual differences research, the order of tests (see Tables 1 and 2) and the order of trials within each test was the same for each participant to minimize potential influences of the test procedure on participants’ test performance.

**Fig. 1:**
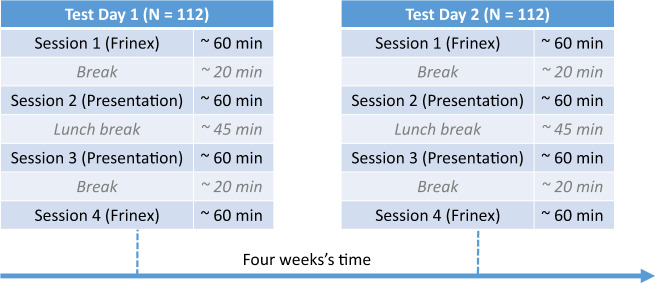
Schematic overview of the study procedure.

As many of the tests were newly developed, no data on test-retest reliability were available. Therefore, all participants were tested twice, with approximately one month’s time between test days (on average 33 days, SD = 8, range = 24–93; see Fig. 1 for a schematic overview of the study procedure). The test procedure on the second day was identical to that of the first day, except that participants did not fill out the intake questionnaire at the beginning of the first session anymore. Participant codes (UUIDs) were augmented with a ‘_2’ extension on the second test day.

The materials, design, and procedure of the individual tests are described below. Picture and text stimuli (font Calibri 17, font colour RGB 0, 102, 102 (green/blue) were presented against a white background. Moreover, if not stated otherwise, auditory stimuli had a sampling rate of 44100 Hz, 16-bit resolution. We used the Dutch Subtlex^12^ and prevalence^13^ databases to retrieve the stimulus words’ frequency and prevalence, respectively. The materials used in each test can be found in the Supplementary Information file.

Below we describe the stimulus materials, procedure and performance indicators of all 33 individual-differences tests. Note that the order of the test descriptions was not the order in which they were administered (see Tables 1 and 2). We first describe the intake questionnaire, the test day-initial questions about well-being, and the audio test procedure. Then, we describe all tests belonging to the ‘Linguistic experience’ domain, followed by descriptions of all tests belonging to the ‘General cognitive skills’ domain and, finally, we describe all ‘Linguistic processing skills’ tests.

#### Questionnaire, questions about well-being, audio tests

The intake questionnaire assessed general demographic information (e.g., sex, age, handedness, education), language background and musical training, as well as potential (developmental) disorders and other medical conditions (see Supplementary Information for all questions). After completion of the questionnaire, participants responded to three questions assessing (1) how well they slept the night before the test day, (2) how their mood was on the test day, and (3) how they would rate their health, each by selecting the appropriate option from a drop-down menu (options: very good, good, average, bad, very bad). Next, participants completed a test assessing whether their headsets were calibrated properly (i.e., whether playback and recording of speech worked). To that end, participants first read aloud four Dutch words. The recordings of these words were played back to them in random order and they indicated via mouse click which of the words they heard. This procedure was repeated until the participant reached 100% accuracy.

### Linguistic experience tests

*1. Stairs4Words*. We used Stairs4Words, a newly developed adaptive test, to assess receptive vocabulary size. Owing to a serious programming error, we strongly discourse the usage of these data (see the Usage Notes section for details). In order to be open and transparent in reporting our results, however, we describe the test procedure and make available the raw data.

On each trial of the test, participants saw a word or a non-word foil and indicated whether or not they knew the item. They were told that ‘knowing a word’ implied having heard or read the word before, and having at least a general idea of its meaning. Participants were informed that some of the stimuli were made-up non-words and that they thus had to carefully consider whether they knew a word or not.

The selection of words and non-words was based on prevalence norms (i.e., norms specifying which proportions of a sample knew the words) provided by Keuleers *et al*.^13^. The database by Keuleers and colleagues contains prevalence measures for approximately 54.000 Dutch words. These measures approximate to what extent a given word is known to the whole population. The words were sorted by prevalence and then divided into 53 bands of 1000 words and one band of 582 words. We selected 40 words from each band, amounting to an item bank of 2160 words covering the entire prevalence range (from words known by >99% of the sample to words known by <1% of the sample) in 54 bands. Words were selected to have similar prevalence values across males and females, different age groups, and Belgian and Dutch speakers. Plural forms, past tense forms of verbs, first person singular forms of verbs and English loanwords were not selected. We devised two parallel versions of the test that were identical in structure and task but used different words in each band. That is, the 40 words selected from each band were evenly distributed across both versions of the test.

The test began with the presentation of an easy word, randomly sampled from band 20 (representing words known by >95% of the norming sample), shown in the centre of the screen. Participants responded by mouse-clicking on an ‘I know the word’ (right-hand side) or ‘I don’t know the word’ (left-hand side) button, respectively, positioned under the target word. In case of a positive response (‘I know the word’), the next word item was harder than the previous one (it came from the next higher prevalence band). In case of a negative response (I don’t know the word), participants were presented with another word from the same difficulty band. Non-words were randomly interspersed with the existing words at a ratio of 6:2 (two non-words on six existing words). Two non-word trials were never presented in succession. Non-words required a negative response (I don’t know the word); a correct rejection also moved the participant to a more difficult band.

This process continued until participants made two successive ‘mistakes’: either (a) indicating twice in a row that they did not know an existing word or (b) indicating that they did not know an existing word *and* indicating they knew a non-existing word. This ‘fast-track’ part of the test was followed by a ‘fine-tuning’ part using a staircase procedure, as is often used in psychometric testing^14^. The crucial difference to the fast-track, not noticeable to the participants, was that in order to move to a more difficult band of items, they needed to respond correctly to four items (three words and a foil) in a row. A single error moved them to a lower band.

The test was untimed and ended after an ‘incorrect’ response (no to an existing word or yes to a non-existing word) occurring after two consecutive oscillations between two adjacent bands. The test also ended when all test words from a given had been used up such that no test word was available for presentation. In the latter case, the number of the band most frequently visited during the fine-tuning part was the end score. In case the test was terminated after two consecutive oscillations between adjacent bands, the lower number of the two bands was the end score. Participants carried out both test versions and received two scores. The participant’s performance indicator was operationalized as the average of both scores.

*2. Peabody picture vocabulary test*. We used a digitized version of the Dutch Peabody picture vocabulary test^15,16^ (PPVT) as a second test for assessing receptive vocabulary size. On each trial, participants first previewed four numbered line drawings on their screen. When they were ready, they pressed the space bar on their keyboard to hear the probe word. They had to indicate which of the four pictures best corresponded to the meaning of the spoken word by clicking on it. Participants could listen to the probe word as often as they wanted, but had to listen to it at least once before a response was recorded. The test had 17 blocks, each consisting of twelve items of roughly the same difficulty. The test started at block 13 (normed entry block for participants aged between 18 and 35). Based on their performance (four or fewer errors in block 13), participants’ next block was either more difficult (block 14) or easier (block 12) than the entry block. The test terminated when more than eight errors were made within a block that was not the starting block or when a participant reached the last item of the test. The participant’s end score was the difference between the item number of the last test word and the number of errors participants made. As prescribed by the test manual, this score was corrected for age, using Dutch norms provided in the test manual (‘WBQ’ tables), which were then transformed into percentiles constituting the performance indicator.

*3. Spelling test*. We designed a test to assess language users’ spelling skills. The test consisted of a list of 60 Dutch words whose spelling adult language users often find difficult. These concern for example the correct use of diaeresis (i.e., ‘bacteriën’, bacteria), the use of double consonants in plural forms (‘slimmeriken’, wise guys), and use of ei/ij (diphthong [ɛi],. e.g. ‘allerlei’, all kinds). Participants were presented with a list of 60 words, in pseudo-random order, divided into three columns of 20 words each. Half of the test words was spelled incorrectly. The ratio of correctly and incorrectly spelled words was not known to the participants. Participants were instructed to use their mouse to click the boxes next to words they thought were spelled incorrectly. The participant’s performance indicator was the proportion correctly categorized misspelled words minus the proportion incorrectly selected words that were spelled correctly.

*4. Author recognition test*. We included a digital version of the Dutch author recognition test^17^. Participants were presented with a written list of 132 names, divided into three columns of 44 words each. They had to indicate which of the listed persons were authors (e.g., Roald Dahl, Nicci French). Ninety of the listed persons were authors and 42 were non-author foils. Authors and non-authors were listed in pseudo-random order and the ratio of authors/non-authors was not known to participants. The performance indicator was the proportion of correctly identified authors minus the proportion non-authors wrongly selected.

*5. Idiom recognition test*. Participants’ knowledge of Dutch idiomatic expressions was tested using an idiom recognition test. On each trial, the participants were presented with a Dutch idiom, such as ‘tussen de regels doorlezen’ (to read between the lines) and a set of four candidate meanings. They had to select the correct meaning among the set of four candidates.

We selected a subset of ten items from the normative database described by Hubers, Cucchiarini, Strik and Dijkstra^18^. In Hubers *et al*.’s study, Dutch native speakers (on average 26 speakers per item) rated 374 different idiomatic expressions on various dimensions and carried out a multiple-choice meaning recognition test. To vary the item difficulty in our test, the ten selected items ranged between 1.35 and 4.39 on the familiarity rating dimension (1–5 scale) and between 0.15 and 1 in meaning recognition accuracy.

In the present test, an idiom was presented at the top of the screen and four meaning candidates were shown underneath in four quadrants of the screen. The position of the target meaning was varied across trials. Both the idiom and the four candidate meanings could be listened to (by mouse-clicking on a loudspeaker icon) to account for the possibility that some idioms are predominantly encountered in the spoken modality and to reduce the influence of reading skill on recognition performance. The performance indicator was the proportion of correctly recognized idioms.

*6. Prescriptive grammar test*. To assess participants’ knowledge of Dutch prescriptive grammar, a recently developed grammaticality judgment test^19,20^ was used. Participants heard spoken sentences and indicated for each of them whether they thought it was a correct Dutch sentence. The sentences featured five grammatical categories (eight trials per category, 50% correct), which adult native speakers of Dutch often find difficult to use correctly: personal pronouns (‘ze’, they vs. ‘hun’, their; ‘ik’, I vs. ‘mij’, me), comparatives (‘als’, as vs. ‘dan’, than), relative pronouns (‘die’, this vs. ‘dat’, that) and participle formation of complex verbs (e.g., ‘stofzuigen’, to vacuum). Stimuli were recorded in a soundproof booth. Average sentence duration was 4344 ms (*SD* = 653, range = 3056–5901).

Each trial started with the presentation of a fixation cross, which coincided with the playback of the spoken sentence. The fixation cross remained in view for the duration of the sentence. Each sentence was presented only once. Participants could respond during or after the presentation of the sentence by mouse-clicking on the appropriate button on the screen (labelled ‘correct’, right-hand position and ‘incorrect’, left-hand position). The mouse-click terminated the trial. The inter-trial interval was 500 ms. The performance indicator was the proportion of correct responses.

*7. Syntest*. This sentence-picture verification test assessed participants’ knowledge of various syntactic sentence structures. We used an extended version of the Syntest recently developed by Janssen, de Swart, Roelofs, Kessels, and Piai^21^, which was primarily designed for testing clinical populations (e.g. aphasic patients). By adding additional, more complex items to the test, we aimed at making the test suitable (i.e., harder) for unimpaired participants. However, our analyses revealed close-to-ceiling performance indicating that the test was too easy (see Usage Notes section). We therefore discourage using these data.

The participants’ task was to listen to a spoken sentence and select the picture among four alternatives that best matched the contents of the sentence. Before the test, participants were familiarized with a set of four generic cartoon characters and their labels (‘jongen’, boy, ‘meisje’, girl, ‘man’, man, ‘vrouw’, woman). These characters were shown in the test pictures carrying out an action (e.g. to run, laugh, or interact with other characters). The four test pictures of a trial were very similar and differed mostly in grammatical role assignment (i.e., who is doing what, who is doing what to whom). The test consisted of 35 experimental trials, divided into seven syntactic categories (e.g. active vs. passive voice, complex relative clauses, clefts) with five items each. Item presentation was blocked by category and item difficulty increased over the course of the test.

On each trial, participants saw the set of four pictures, one in each quadrant of the screen. Upon presentation of the pictures, the spoken sentence was played back. Participants could listen to the sentence as often as they wished. They selected the target picture by mouse click. The position of the target picture was pseudo-randomized. Participants completed one practice trial before the experimental trials. The performance indicator was the proportion of correct responses.

### General cognitive skills tests: Non-verbal processing speed

#### Auditory reaction time (A-RT) tests

Two tests tapping response speed to auditory stimuli were used^10^. In both cases, the task was to respond as fast as possible to the onset of an auditory stimulus.

*8. Auditory simple reaction time test*. In the simple A-RT test, participants saw a fixation cross in the centre of the screen. After an interval varying between one and three seconds, a sine tone (550 Hz, 400 ms) was played. Participants were instructed to press the right-hand button of the button box as soon as they heard the tone, which terminated the trial. After one second, the next trial began. The simple A-RT test consisted of 20 test trials, preceded by eight practice trials. The performance indicator was participants’ mean RT.

*9. Auditory choice reaction time test*. In the choice A-RT test, the task was to respond as quickly as possible to each of two auditory stimuli, presented in pseudo-random order, by pressing the associated button. Participants first saw a fixation cross in the centre of the screen. After an interval varying between one and three seconds, a low or high sine tone (300 and 800 Hz, respectively, both 400 ms) was played. Participants pressed the right-hand button on the button box when they heard the high tone, and the left-hand button on the button box when they heard the low tone. The inter-trial interval was one second. The choice A-RT test consisted of 40 experimental trials, preceded by 16 practice trials. Average RT was calculated based on correct responses.

*10. Letter comparison test*. The letter comparison test was adapted from a paper-and-pencil task developed by Salthouse and colleagues^22,23^. We used the computerized version by Huettig and Janse^9^. The participants’ task was to decide whether or not two strings of letters were identical. The first test block featured pairs of three-letter strings (e.g. TZF) and the second block pairs of six-letter strings (e.g. RNHKTG). Letters were presented in a large monospaced font (Courier New, font size 70). The space between the letter strings in a pair was 300 pixels. Participants were asked to indicate as quickly and accurately as possible whether the two letter strings were the same or different by pressing the left-hand button on the button box (‘different’) or the right-hand button on the button box (‘same’). To start, there were six three-letter practice trials. Each test block consisted of twelve trials containing identical strings and twelve trials with different strings. Each trial started with the presentation of a fixation cross, which stayed on the screen for 600 ms. Subsequently, the two letter strings were presented until a response was made. The next trial began after one second. Response speed, as performance indicator, was determined by averaging over participants’ correct responses.

#### Visual reaction time tests (V-RT)

These tests were the visual counterparts of the A-RT tests. Participants were asked to respond as quickly as possible to the onset of a visual stimulus by pressing the correct button on the button box. Both V-RT tests are based on tests designed by Deary, Liewald, and Nissan^24^.

*11. Visual simple reaction time test*. On each trial of the simple V-RT test, participants first saw a fixation cross in the centre of the screen. After an interval varying between one and three seconds, it was replaced by a line drawing of a triangle (200 × 200 pixels, black contours). Participants were instructed to press the right-hand button on the button box as soon as the triangle appeared. The response terminated the trial. After an inter-trial interval of one second, the next trial began. The test consisted of 20 experimental trials, preceded by eight practice trials. The performance indicator was participants’ average RT.

*12. Visual choice reaction time test*. On each trial of the choice V-RT test, participants first saw a fixation cross in the centre of the screen. After an interval varying between one and three seconds, it was replaced with a line drawing of either a star or a circle (black contours, 200 × 200 pixels). Participants were instructed to press the left-hand button on the button box as fast as possible upon appearance of a star, and the right-hand button of the button box upon appearance of a circle. The star and circle appeared equally often throughout the experiment and in pseudo-random order. The test consisted of 40 experimental trials, preceded by 16 practice trials. The performance indicator was participants’ average RT on correct trials.

### General cognitive skills tests: Working memory

*13. Digit span test (forward and backward)*. We used a computerized version of the digit span test^25^ to assess auditory working memory. At the beginning of each trial, a fixation cross appeared in the centre of the screen. After 2000 ms, the playback of a sequence of spoken digits was initiated while the fixation cross remained in view. Digits were presented at approximately 500 ms intervals. Following auditory playback, a response field appeared at the bottom of the screen and participants were requested to type in the digits in the order they were encountered (forward version) or in the reversed order (backward version). The first two trials of each version featured a two-digit sequence; these were considered practice trials. When at least one of two consecutive trials of the same length was recalled correctly, the sequence was extended by one digit. The test ended when two consecutive trials for a sequence length were responded to incorrectly or when participants reached the end of the test (nine digits in the forward version, eight digits in the backward version). Separate performance indicators were obtained for forward and backward versions, operationalized as the sum of correct responses per version^26^.

*14. Corsi block clicking test (forward and backward)*. This test was included to assess visual-spatial short-term memory capacity^26–29^ and formed the counter-part to the auditory digit span test. Participants were presented with nine squares, which were randomly distributed across the screen. Different squares lit up successively at a rate of one square per second. At the end of a sequence, a green frame appeared around the display, prompting participants for a response. The participants were instructed to repeat the sequence by clicking on the respective squares, either by forward repetition or backward reproduction. When clicking on the squares, they briefly lit up in black for 200 ms and then turned blank again. After having reproduced the sequence in forward or backward fashion, participants clicked on a button at the bottom of the screen to proceed to the next trial. They were familiarized with the test, completing two practice trials of two-square sequences. The first experimental trial featured a sequence length of three squares. The sequence length was extended by one square when at least one of two consecutive trials was recalled correctly. The test ended when two consecutive trials for a given sequence length were responded to incorrectly or when participants reached the end of the test (sequence of nine blocks in both versions). The performance indicator was the sum of correct responses on experimental trials in forward and backward versions, respectively.

### General cognitive skills tests: Inhibition

*15. Eriksen Flanker test*. We included the Eriksen Flanker test, which is assumed to measure selective inhibition^30^. Participants responded to the direction of a central arrow (‘ < ’ or ‘ > ’) flanked by either neutral distractors (neutral condition, e.g. ‘-- > --‘) or by distractors pointing in the same direction (congruent condition, e.g. ‘>>>>>’) or the opposite direction (incongruent condition, e.g. ‘<<><<’). On each trial, participants first saw a fixation cross for 600 ms, then the target stimulus was presented. The trial was terminated by participants’ response and the next trial started after one second. Six practice trials (two of each trial type) were followed by 72 experimental trials (24 of each trial type in pseudo-random order). The performance indicator was the Flanker effect (mean RT to incongruent trials minus mean RT to congruent trials), reflecting participants’ ability to inhibit task-irrelevant distractor information.

*16. Antisaccade test*. As a second test tapping inhibitory skills, we included the antisaccade test^31^. This test measured participants’ ability to deliberately inhibit dominant, automatic, or prepotent responses. On each trial, a fixation point was first presented in the middle of the computer screen for a pseudo-randomized interval between 1 and 3 seconds. A visual cue (0.4°) was then presented on one side of the screen (e.g., left) for 225 ms, followed by the presentation of a target stimulus (2.0°) on the opposite side (e.g., right). The target was shown for 150 ms before being masked. The visual cue was a black square, and the target stimulus was an arrow pointing to the left, the right or upwards. Participants’ task was to indicate the direction of the arrow by pressing the associated arrow-button on the keyboard. They were instructed to use their dominant hand and place index, middle, and ring finger on the left-arrow, upper-arrow, and right-arrow, respectively. Participants’ response terminated the trial. The inter-trial interval was one second.

Given that the target appeared for only 150 ms before being masked, participants were required to inhibit the reflexive response of looking at the initial cue because doing so would make it difficult to identify the direction of the arrow. The cues and targets were presented 10 cm away from the fixation point (on opposite sides) and the participants were seated approximately 50 cm from the computer monitor. The participants carried out 22 practice trials and then completed 90 target trials (30 of each arrow type) in pseudo-random order. The proportion of experimental trials responded to correctly served as the performance indicator.

### General cognitive skills test: Non-verbal intelligence

*17. Raven’s advanced progressive matrices test.* To assess non-verbal intelligence, a computerized version of Ravens’ advanced progressive matrices^32^ was used. On each trial, participants indicated which of eight possible shapes completed a matrix of geometric patterns. They selected the shape by clicking on it. Participants could skip items by clicking on a button labelled ‘Skip’; these items were shown again at the end of the test. When they did not know the answer to a skipped item, participants could click on an ‘I don’t know’ button. There were 36 test items, increasing in difficulty, preceded by six untimed practice items. Participants had 20 minutes to complete the experimental items. Throughout the test, a clock in the right top corner of the screen showed the time remaining. The performance indicator was the proportion of correctly answered experimental items.

### Linguistic processing skills tests: Word production

*18. Picture naming test*. To test participants’ word production skills, we included a picture-naming test. In this test, participants were shown photographs of common objects and were asked to name these as quickly as possible^10^. The test materials consisted of 40 photographs, taken from de Groot *et al*.^33^ or retrieved online via a search engine. The object names varied substantially in lexical frequency (average ZipfF = 3.83, *SD* = 0.88, range = 2.04–5.39; as retrieved from the Subtlex Corpus^12^). As recommended by van Heuven *et al*.^34^, we used Zipf-transformed word frequency values (ZipfF), which were operationalized as log10(frequency per million words) + 3. Prevalence norms^13^ indicated that the object names were likely to be known by all participants (average prevalence 99.6%, *SD* = 0.4, range 97.7–100). The average number of phonological neighbours (sum of additions, substitutions, deletions of segments) of the object names was 4.05 (*SD* = 3.99, range = 0–18; as retrieved from Clearpond^35^). Four additional photographs were used as practice trials. All pictures were scaled to 300 × 300 pixels.

The test began with the presentation of the four practice items. On each trial, participants first saw a fixation cross in the centre of the screen, which was shown for 800 ms. Then, the target picture was shown for three seconds. After an inter-trial interval of one second, the next trial began. Participants’ utterances were recorded. Naming accuracy as well as word onsets and offsets were coded offline using the Praat software^36^. The performance indicator was participants’ average onset latency for correctly named experimental trials. In addition to incorrect picture names, self-corrections, hesitations, diminutives, and plural forms were counted as incorrect.

*19. Rapid automatized naming (RAN)*. This test was included to assess speed of word form access during word production. Participants were first familiarized with a set of five line drawings and their names. During this familiarization phase, participants saw the line drawings and heard a recording of a female speaker name each of them. Subsequently, they saw the line drawings randomly arranged in an array consisting of five rows of six objects; each object was thus repeated six times throughout the array. At the beginning of the trial, a fixation cross was shown in the left upper corner of the screen, at the position of the first object of the array. The fixation cross disappeared after two seconds and the object array was shown. Participants named all objects row-by-row, from left to right. They were instructed to name them as quickly as possible, while making as few mistakes as possible. Upon completion, they pressed the spacebar.

We used a version of the RAN developed for Dutch at the Max Planck Institute for Psycholinguistics^37^. The test featured four sets of five line drawings whose names orthogonally varied in word frequency, and neighbourhood density, thereby comprising an easy set (high frequency: *M* = 4.94, high density: *M* = 25, set 1), a hard set (low frequency: *M* = 3.4, low density: *M* = 8, set 4) and two intermediate sets (high frequency: *M* = 4.95, low density: *M* = 10, set 2; low frequency: *M* = 3.45, high density: *M* = 22, set 3). Each set was named twice, featuring different orders of the line drawings.

Naming accuracy and latencies were coded offline using Praat^36^. For each trial, a ratio was calculated by dividing the number of correctly named objects by the total speech duration for that trial. These eight scores were then averaged, yielding one performance indicator per participant. A trial was excluded when substantial amounts of errors were made: For instance, one of the objects was consistently named incorrectly, a participant did not complete the run (i.e., stopped speaking half way through), or had misunderstood the instructions (i.e., named the items from top to bottom instead of left to right). Applying this criterion would lead to the exclusion of 34 participants, almost one third of the participants. As for tests 1. Stairs4Words and 7. Syntest, we therefore discourage researchers from using the RAN test data (see Usage Notes section).

*20. Antonym production.* As an additional test of lexical access ability, focusing on the activation of semantic representations, an open-ended, untimed antonym production test was included. This test was recently developed by Mainz *et al*.^38^ at the Max Planck Institute for Psycholinguistics. Participants were provided with a word cue and were instructed to produce its antonym (e.g., cue: hot, antonym: cold). The test consisted of 28 trials (3 practice and 25 experimental trials). Before each trial, participants saw a fixation cross for 500 ms, after which the cue word was presented (in written form *and* once in spoken form). Participants provided a spoken response and their answer was recorded. They clicked on a button on the screen to advance to the next trial. The cue words varied in word frequency^12^ (*M* = 3.84, *SD* = 1.41, range = 1.70–5.26) but not in prevalence^13^ (*M* = 1.00, *SD* = 0.04, range = 0.85–1.00) and thus in how easily an antonym could be retrieved. Accuracy was coded offline. The performance indicator was the proportion of correct experimental trials.

*21. Verbal fluency*. We included a digitized version of the verbal fluency test used in an earlier study with Dutch participants^39^. In the first part of this test, participants were presented with two semantic categories (‘animals’ and ‘food & drinks’), one at a time. They were told they would have one minute to name as many words belonging to the categories as they could. The second part of the test was similar, however, participants were now presented with a letter (‘M’ and ‘S’) and had to produce as many unique words as possible beginning with that letter. Each trial started with a timer counting down from three to zero indicating the start of the recording. Then, the category or letter was presented. A timer was shown on the screen counting down from 60 to 0. After one minute, the next trial started. Speech was transcribed and scored offline (see Shao *et al*., for scoring details). For each category/letter, we counted the number of unique words produced within one minute and calculated average scores over both semantic categories and both letters, yielding two performance indicators per participant.

*22. Maximal speech rate.* To assess their maximal speech rate, participants were asked to recite the months of the year as quickly as possible with good pronunciation. This test had been piloted in an earlier study carried out at the Max Planck Institute for Psycholinguistics and showed a good degree of variability between participants (compared to reciting the days of the week or simple counting, which most participants did at great speed). Participants performed two runs. Speech was coded offline using Praat^36^. The participants’ maximal speech rate was the average speech duration of both runs. In case only one of the two runs was correct (e.g., one or more months were skipped), the speech duration of the correct run was the performance indicator.

*23. One-minute-test*. During the one-minute-test^40^ (Dutch één-minuut-test), participants were presented with a list consisting of 116 Dutch words. Participants were instructed to read aloud as many words as possible within one minute. The words became progressively more difficult to read in terms of syllable length (range: 1–5 syllables). To calculate the performance indicator, the number of errors (incorrectly pronounced words) was subtracted from the number of words read within one minute.

*24. Klepel test*. During the Klepel test^41^, participants were presented with a list of 116 non-words. They were instructed to read aloud as many non-words as possible within two minutes. The non-words became progressively more difficult in a similar fashion as in the 23. One-minute-test. Previous pilot studies had shown that the majority of participants were able to produce all non-words within two minutes. Therefore, the performance indicator here was operationalized as the number of non-words read correctly within the first minute of the test minus the number of errors in that first minute. Notwithstanding, the database contains the item coding for both one- and two-minute reading times.

### Linguistic processing skills tests: Word comprehension

*25. Monitoring in noise in lists*. The following set of three tasks was recently developed at the Max Planck Institute for Psycholinguistics to assess word comprehension processes. The three tasks involved monitoring 30 spoken lists of words or non-words for the occurrence of targets that matched an auditory cue or bore a semantic relationship with the cue^42^. The cue, a monosyllabic Dutch noun or non-word, was provided at the beginning of each trial and varied from trial to trial. While the cue was presented in the clear, the subsequent lists of three to six words or non-words were presented in stationary speech-shaped background noise. On the first trial of each task, the signal-to-noise ratio (SNR) was set to 0 dB (equal proportions of speech and noise) and decreased from trial to trial in steps of two until −18 dB (more portions of noise than speech). Within each task, each SNR was presented three times (i.e., three rounds of going from SNR 0 dB to SNR −18 dB). Twenty of the 30 trials were target-present trials and required a button press; ten were target-absent trials and required no button press. There were two target-present and one target-absent trials per SNR, presented in pseudo-random order.

The trial structure in all three tasks was as follows: Participants pressed the right-hand button on their button box to start the trial; a fixation cross was displayed in the centre of the screen and after 200 ms the spoken cue was presented. After an interval of one second, the list was presented, with 500 ms of silence between words or non-words. Participants were instructed to press the right-hand button on their button box as soon as they recognized the cue within the list or (in the meaning monitoring task) a word semantically related to it. Pressing the button terminated the trial. Button presses were counted as correct responses when they occurred within the playback period of the target word or non-word, or 500 ms after its offset. Before the first experimental trial, participants completed three practice trials. The performance indicator was the proportion of correct responses to target-present trials minus the proportion of false alarms on target-absent trials. Separate performance indicators were computed for each of the three monitoring tasks.

*Task 1: Non-word monitoring in non-word lists*. This task assessed participants’ ability to extract a speech signal from noisy input and their ability to map the extracted phonological information onto stored mental representations. On each trial, participants listened to a mono-syllabic non-word cue prior to a list of mono- and disyllabic non-words. Their task was to monitor for the non-word cue and to press a button upon detection. Non-word cues and distractors were created on the basis of existing Dutch words using the non-word generator Wuggy^43^. The generated non-words matched the number of letters and the number of phonemes in the original word.

Audio recordings of the stimuli were made in a soundproof booth. The same speaker produced the stimuli for all three tasks. Speech-shaped noise was added to each individual file using Praat software^36^. To that end, the original recordings were down-sampled to 16 kHz to match the sampling frequency of the noise. Two-hundred and five milliseconds of ramping noise preceded the speech onsets, providing a head start for the listeners to get used to the noise. Noise was added over the entire file. Peak intensity in all (clear and noise-added) files was set to 65 dB.

The non-word monitoring in non-word lists task consisted of 30 trials (see (1) for examples): ten were target-absent trials, where the cue non-word did not appear in the list; ten were target-present trials; and ten were ‘target-present plus foil trials’. On these trials, the target was preceded by a non-word foil that overlapped with the target in phonological onset (on average two phonemes). We included these trials to ensure that participants listened carefully until the end of each non-word. We used different recordings of the same non-word for cue and target presentations to avoid response strategies based on low-level perceptual matching. Non-word cues were on average 736 ms long (*SD* = 130, range = 473–1139); targets were on average 752 ms long (*SD* = 120, range = 589–1049). The target word position within the list varied from trial to trial; it never occurred in list-initial or list-final position.

1.
  - Target present: Cue non-word: **broon**. List: nijmsaard – wulen – pluif – **broon –** swi
  - Target + foil: Cue non-word: **broog**. List: dauk – broopkimp – **broog** – knekgel
  - Target absent: Cue non-word: **praan**. List: veg – gebog – siekoed – fonguin

*Task 2: Word-form monitoring in word lists*. This task measured lexical access ability. It was identical to the non-word monitoring in non-word lists task, except that the cues and distractors were existing Dutch words. The words varied in word frequency^12^ (cues: *M* = 4.16, *SD* = 0.95, range = 2.36–6.42; distractors: *M* = 3.97, *SD* = 0.95, range = 1.30–6.60), but were all high in prevalence^13^ (cues: *M* = 0.99, *SD* = 0.01, range = 0.94–1.00; *M* = 0.99, *SD* = 0.02, range = 0.82–1.00). Cue and distractor words were semantically unrelated. As in the non-word monitoring task, half of the target-present trials contained a foil that overlapped with the target in phonological onset. The stimuli were processed as in the non-word monitoring task. Word cues were on average 764 ms long (*SD* = 126, range = 501–996); targets were on average 792 ms long (*SD* = 109, range = 552–979).

(2)
  - Target present: Cue word: **spook** (‘*ghost*’**)**. List: ruil (‘*exchange*’) – vlecht (‘*braid*’) – klomp (‘*clog*’) – **spook** – applaus (‘*applause*’)
  - Target + foil: Cue word: **oog** (‘*eye*’). List: spoor (‘*track*’) – oostgrens (‘*eastern border*’) – **oog** – versie (‘*version*’)
  - Target absent: Cue word: **uil** (‘*owl*’). List: folder (‘*flyer*’) – pil (‘*pill*’) – lijn (‘*line*’) – ballon (‘*balloon*’)

*Task 3: Meaning monitoring in word lists*. This task assessed semantic access during word recognition. It was identical to the previous two tasks, except that participants now monitored for words that were moderately to highly semantically related, rather than identical to the cue. Cues and semantically related targets were monosyllabic Dutch nouns, varying in word frequency^12^ (cues: *M* = 3.99, *SD* = 0.84, range = 2.26–5.91; targets: *M* = 4.23; *SD* = 0.91, range 1.95–6.15). All target and cue words were high in word prevalence^13^ (cues: *M* = 0.99, *SD* = 0.02, range = 0.90–1.00; targets: *M* = 1.00, *SD* = 0.00, range = 0.99–1.00. The semantic relationship between cue and target was operationalized as forward association strength^44^ (FAS). FAS was on average 0.21 (*SD* = 0.20, range = 0–0.64). In contrast to both previous tasks, no target-present plus foil trials were included. Distractors were semantically unrelated to each other and to the cue.

(3)
  - Target present: Cue word: **prins** (‘*prince*’**)**. List: nijlpaard (‘*hippo*’) – regen (‘*rain*’) – druif (‘*grape*’) – **kroon** (‘*crown*’) – ski (‘*ski*’)
  - Target absent: Cue word: **nest** (‘*nest*’). List: vel (‘*skin*’) – gebak (‘*pastry*’) – sieraad (‘*ornament*’) – fontein (‘*fountain*’)

*26. Rhyme judgment*. Similar to the non-word monitoring task, the rhyme judgment test assessed phonological mapping abilities. On each trial, participants were presented with two monosyllabic non-words and were asked to judge as fast as possible whether they rhymed. Rhyme overlap was defined as an overlap in the vowel and the following consonant(s). The test consisted of 40 experimental trials: 24 of which were ‘rhyming trials’ (e.g., ‘noost’-‘woost’) and required a yes-response. Eight were ‘non-rhyming trials’ (e.g., ‘beus’-‘fuug’) and required a no-response. Another eight trials were ‘non-rhyming foils’, which featured non-word pairs sharing the vowel but not the following consonants (e.g., ‘bruip’, ‘fluik’). These trials were included to ensure that participants listened carefully until the end of the second non-word^45^; they also required a no-response. Rhyming and non-rhyming trials were presented in a pseudo-random order. Prior to the experimental trials, participants completed four practice trials (two rhyming, one non-rhyming and one non-rhyming foil, in random order). The non-words used in the test were based on existing Dutch words and were generated using Wuggy^43^. Recordings were made in a sound-shielded booth. Length of first and second non-words in the three trial types were as follows: Rhyming items: first word average = 670 ms, *SD* = 86, range = 470–810; second word average = 621 ms, *SD* = 74, range = 521–770; Non-rhyming items: first word average = 653 ms, *SD* = 58, range = 547–720; second word average = 562, *SD* = 64, range = 461–638; Non-rhyming foils: first word average = 588 ms, *SD* = 88, range = 443–701; second word average = 550 ms, *SD* = 62, range = 449–649.

The trial structure was as follows: A fixation cross appeared in the centre of the screen for 500 ms. Then, the first non-word was played back. Five-hundred ms after the offset of the first non-word, the second non-word was played back. Participants were instructed to indicate as fast as possible whether the non-words rhymed by pushing a key on the button box (right-hand button for ‘rhyme’, left-hand button for ‘no rhyme’). Their response and its latency, measured from the onset of the second non-word, were recorded. The button press terminated the trial. The inter-trial interval was 2000 ms. Participants’ average RT, based on correct responses to rhyming trials, was taken as performance indicator.

*27. Auditory lexical decision*. This test complemented the word form monitoring in noise task and was assumed to measure lexical access speed. Participants were instructed to listen to the recording of a word or non-word and judge whether it existed in the Dutch language^10^. To that end, sixty Dutch words were selected from the Subtlex database^12^. The words varied substantially in word frequency (average ZipfF = 3.65, *SD* = 0.85, range = 2.04–5.66), but were highly prevalent and thus well known to all participants (average prevalence^13^ = 99.6, *SD* = 0.5, range = 97.3–100). The average number of phonological neighbours (as retrieved from Clearpond^35^, defined as deletions, additions, and substitutions) was 2.8 (*SD* = 3, range = 0–12). For each word, a matched non-word was created using Wuggy^43^ (applying the same constraints as for the non-word monitoring and the rhyme judgement task). One additional word and two non-words were used as practice trials. Recordings were made in a sound-attenuated booth. The average stimulus length was 746 ms (*SD* = 93, range = 568–967) for words and 808 ms (*SD* = 119, range = 540–1164) for non-words.

The test began with the presentation of the practice items, followed by the experimental items, both presented in a pseudo-random order. At the beginning of each trial, a fixation cross was shown in the centre of the screen for 300 ms. Then the stimulus was presented. Participants were instructed to listen carefully to each stimulus and decide as quickly as possibly whether or not it was an existing Dutch word. They pressed the right-hand button on their button box to give a ‘word’ response and the left-hand button to give a ‘not a word’ response. The response terminated the trial. After one second, the next trial began. The response latency was the time interval between the spoken word onset and the button press. The performance indicator was the average response latency on correct responses to words.

*28. Semantic categorization*. As a second test tapping semantic access during spoken word recognition, we included a semantic categorization test. There were two test blocks. At the beginning of a block participants were presented with a semantic category, ‘professions’ for block 1 and ‘means of transportation’ for block 2. On each of the following trials, they heard a word and had to judge whether or not it was a member of that category. We selected professions and means of transportation categories as they featured enough easy-to-recognize members. Each test block consisted of 32 trials, 20 category members and 12 distractors. Each part was preceded by four practice trials (two category members, two distractors). Targets and distractors were matched on word frequency (professions: ZipfF^12^ *M* = 3.63, *SD* = .23, range = 3.25–4.07; means of transportation *M* = 3.27, *SD* = .51, range = 2.15–4.12). All words used in the test were highly prevalent^13^ (known to 99–100% of all people). Recordings were made in a sound-shielded booth. The recordings of the words had the following duration: Professions: M = 880 ms (SD = 155, range = 657–1186), Distractors: M = 800 ms (SD = 160, range = 569–1047); Means of transportation: M: 724 ms (SD = 143, range = 404–957), distractors: M = 726 ms (SD = 169, range = 491–1106).

The trial structure was as follows: Participants were first presented with a fixation cross for 500 ms after which they heard the spoken word. They were instructed to indicate as fast as possible whether the word belonged to the category provided beforehand by pushing the associated key on the button box (right-hand button for ‘yes, this word belongs to the category’ and left-hand button for ‘no, this word does not belong to the category’). The inter-trial interval was 2000 ms. Response latency was measured from the onset of the spoken word. Participants’ average RT, based on correct responses to semantic category members, was taken as the performance indicator.

### Linguistic processing skills tests: Sentence production

*29. Phrase and sentence generation*. This new phrase and sentence generation test was recently developed at the Max Planck Institute for Psycholinguistics. Participants were asked to generate descriptions of objects (Phrase generation) or scenes (Sentence generation) varying in structure and complexity. All displays referred to a small set of objects and actions, which minimized between-item variability due to differences in ease of lexical access. The phrase and sentence generation tests were implemented as one application in Presentation©.

#### Phrase generation

In the phrase generation test, participants were first familiarized with a set of 16 pictures of common objects, selected from the database by de Groot *et al*.^33^. Four of these pictures were used for practice trials. The remaining twelve pictures were experimental items. The object names were monosyllabic and high in word frequency^12^ (M = 4.58, SD = 0.42, range = 4.05–5.23) and prevalence^13^ (M = 1.00, SD = 0.00, range = 0.99–1.00). Dutch nouns differ in grammatical gender (common or neuter) and the corresponding definite determiners (de or het). Half of the object names had common gender and half had neuter gender.

The phrase generation test consisted of four blocks featuring twelve trials each. Block 1 was preceded by four practice trials, Blocks 2 to 4 were each preceded by two practice trials. The twelve experimental and the four practice objects were repeated in various combinations across the four blocks. The trial structure was the same in all blocks: A fixation cross was shown in the centre of the screen for 500 ms and was replaced with the to-be-named object or combination of objects. Participants were instructed to name these as quickly as possible. They were instructed to press the right button on their button box after they were done speaking.

In Block 1, participants were asked to name single objects (e.g. ‘hond’, dog). In Block 2, they named pairs of objects shown next to each other in noun phrase conjunctions such as ‘aap en neus’, (monkey and nose). In Block 3, they either saw two or three identical objects and named them in phrases such as ‘drie honden’ (three dogs), or they saw a single object, in blue or yellow and had to produce an adjective-noun phrase, such as ‘blauwe hond’. We opted for the two colour adjectives blue (blauw), yellow (geel) and the two numerals (two, twee, three, drie) as in Dutch these words are perceptually and phonologically distinct from each other. Finally, Block 4 required participants to name two or three objects appearing in one of the two colours in complex adjective noun phrases (e.g. ‘twee blauwe honden’, two blue dogs).

Participants’ responses were recorded, starting with the presentation of the visual stimuli. The recording was terminated one second after participants’ button press or timed out after ten seconds (in case participants forgot to press the button). Speech was coded offline using Praat software^36^. The performance indicator for the phrase generation test was the duration of the participants’ speech (difference between speech onset and speech offset) on correct trials, averaged over trials from Blocks 1–4.

#### Sentence generation

The visual stimuli for the sentence generation test (Blocks 5 to 7) were taken from Menenti *et al*.^46^. They consisted of 20 photographs of two actors, a man and a woman, carrying out different highly prevalent^13^ transitive actions (e.g. interview each other; mean prevalence 1.00, SD = 0.00, 0.99–1.00). The frequency^12^ of the verbs was on average 4.25 (SD = 0.74, range = 3.42–5.78). The actors had been coloured in blue and yellow using Adobe Photoshop©. Each actor appeared equally often in each colour and on the left and right side of the screen. Block 5 featured six trials, Block 6 and 7 featured 12 trials each. Each block was preceded by two practice trials providing participants with the expected sentence structure.

The trial structure in Blocks 5 and 6 was as follows: Participants saw a fixation cross for 500 ms, which was replaced with the presentation of the written transitive verb to be used in the sentence. The verb was displayed for one second, and then the scene was shown, featuring the two actors, one coloured in blue and one in yellow. The actors carried out the action implied by the verb. Participants were instructed to name the yellow person first. Depending on the color-coding of agent and patient, naming the yellow actor elicited either an active or a passive sentence^46^.

In Block 5, participants were required to produce questions (e.g. ‘Interviewt de man de vrouw?’, Is the man interviewing the woman?). Formulating these types of questions in Dutch requires the inflected verb to be placed at the beginning of the sentence. However, as the verb was provided before the scene appeared, the task was rather easy as participants could immediately start speaking and carry out the remaining sentence planning processes as they spoke. Thus, Block 5 was included to provide the participants with the opportunity to get accustomed to the trial structure and the color-coding. Block 6 required the production of active and passive sentences (e.g. ‘De vrouw troost de man.’, The woman is comforting the man. vs. ‘De man wordt door de vrouw gestopt.’, The man is being stopped by the woman.). Block 6 included six active and six passive trials. The trial structure in Block 7 was slightly different compared to the previous blocks. Following the fixation cross, participants saw and read aloud the first part of a sentence (matrix clause), which included a coordinate or subordinate conjunction (‘want’, because or ‘omdat’, due to). By pushing the right button on the button box, they advanced to the next screen, which provided them with the transitive verb to be used in the subsequent scene description. The verb disappeared after one second and, as before, participants saw a scene containing color-coded actors. Their task was to complete the sentence they had read aloud by describing the scene. However, depending on the conjunction the direct object either followed or preceded the verb (as in ‘want de man draagt de vrouw’ or ‘omdat de man de vrouw draagt’). There were six trials of each type. All had active structure.

As in the phrase generation test, participants pressed the right button on their button box when they had completed the description in the sentence generation test. The recording was terminated one second after participants’ button press or timed out after ten seconds. The performance indicator for the sentence generation part was proportion of correct trials (averaged over trials from Blocks 6 and 7).

*30. Spontaneous speech*. In this test, participants were asked to talk freely about three topics: (1) their activities during the last weekend, or any other weekend of their choice, (2) a book or a movie they have recently enjoyed and (3) their dream holiday. These topics were chosen as they were expected to elicit natural speech in the past, present, future and conditional tenses. Such tasks have been used widely in the neuropsychological and aging literature and allow for the analysis of various properties of spontaneous speech including speech rate, fluency (frequency and duration of silent and filled pauses), type-token ratio of words, average word frequency, utterance length, syntactic and conceptual richness. Moreover, in a recent pilot study, we have shown that measures extracted from spontaneous speech correlated positively with participants’ receptive vocabulary size^47^.

Participants were presented with the questions (one at a time) and were given time to conceptualize what they wanted to say. When ready, they clicked on a button to start the recording. After one minute, the recording stopped automatically and participants were presented with the next question. We transcribed and annotated (marking onset and offset of each word) all 672 recordings using Praat software^36^. The text grids containing transcriptions and annotations are made available along with the recordings as a resource for other researchers to explore.

### Linguistic processing skills tests: Sentence comprehension

*31. Gender cue activation during sentence comprehension*. This test assessed whether and to what degree listeners exploit grammatical gender cues for predicting upcoming target words in an unfolding sentence. The first part of the test was a gender judgment task, where participants were presented with 84 common objects^48^ (one at a time) and were instructed to indicate the grammatical gender of the object names (42 ‘de’, common-gender nouns, 42 ‘het’, neuter-gender nouns) by pressing the associated key on their button box (left-hand button for ‘de’, right-hand button for ‘het’). This part was included to tap participants’ accuracy in judging a word’s grammatical gender and to increase their sensitivity toward this type of word knowledge. The average word frequency^12^ of the object names was 4.05 (*SD* = 0.68, range = 2.36–5.66). Prevalence^13^ of the object names was high (*M* = 1.00, *SD* = 0.00 range = 0.98–1.00). Objects with neuter and common gender (‘het’ and ‘de’ items) were presented in pseudo-random order. The participant’s button press terminated the trial.

The second part of the test was inspired by the study by Huettig and Janse^9^. The same objects as in the first part were used. The second part consisted of two practice trials and 40 experimental trials. Each trial featured two objects which differed in grammatical gender. Participants were first presented with a fixation cross for 800 ms in the centre of the screen, followed by the presentation of the two objects. Then, they heard the recording of a question asking on which side of the screen (left or right) one of the two objects was located (e.g., ‘Waar is het weergegeven paard?’, Where is the displayed horse?). Crucially, on half of the trials, the definite article was used (de/het, the); on the other half, the indefinite article was used (‘een’, a; e.g. ‘Waar is een weergegeven boek?’, Where is a displayed book?). Trials featuring the definite article constituted the predictable condition as participants could anticipate, based on the definite article’s grammatical gender, which object would be referred to. Trials featuring the indefinite article constituted the non-predictable condition, as based on the indefinite article, it could not be anticipated which object would be referred to (the indefinite article in Dutch is not marked for gender). The presentation of the spoken sentence was timed such that on each trial participants had three seconds to preview the two objects before the onset of the gender cue in the spoken sentences (i.e., sufficient time to retrieve this type of word information from the displayed objects^9^). The definite articles (predictable condition) were on average 255 ms long (*SD* = 41, range = 170–310); the indefinite articles (non-predictable condition) were 260 ms long (*SD* = 33, range = 197–350). The target words in the predictable condition were on average 518 ms long (*SD* = 119, range 348–813); in the non-predictable condition they were on average 536 ms long (*SD* = 117, range = 391–726). The predictive window (i.e., the interval between determiner onset and target word onset on the 20 predictable trials) was on average 2053 ms long (*SD* = 107, range = 1878–2280).

Participants were instructed to press the appropriate button as soon as they knew which object would be referred to (left-hand button on the button box for left-hand object, right-hand button for right-hand object). The button press terminated the trial. The inter-trial interval was 1000 ms. The target object appeared equally often on the left and right side of the screen. Predictable and non-predictable trials were presented in a pseudo-random order. Response latencies were calculated as the difference between the onset of the target word in the spoken sentences and participants’ button press. A negative value thus reflected a button press before target word onset. The performance indicator was the average RT, based on correct responses, in the predictable condition.

*32. Verb semantics activation during sentence comprehension*. Using a similar paradigm as for the gender cue activation test, this test assessed participants’ ability to use verb-specific selectional restrictions during unfolding spoken sentences^49,50^. On each trial, participants first saw a fixation cross in the centre of the screen for 800 ms, which was replaced with pictures of two common objects^48^ (e.g. an apple and a table; average object-name frequency^12^: 4.13, *SD* = 0.64, range = 2.70–5.52; average object-name prevalence^13^: 1.00, *SD* = 0.01, range = 0.97–1.00). Next, they heard a spoken sentence describing a transitive action carried out by a fictional character (e.g. ‘De man schilt op dit moment een apple.’, The man is peeling at this moment an apple). In the predictable condition, participants could predict based on the verb semantics which of the objects would be referred to before target word onset as only one fulfilled its selectional restrictions (e.g., an apple can be peeled, a table cannot). In the non-predictable condition, this was not the case.

The verbs in the predictable condition were on average 598 ms long (*SD* = 132, range = 406–875); verbs in the non-predictable condition were on average 641 ms long (*SD* = 153, range = 403–1037). Target words in the predictable condition were on average 471 ms long (*SD* = 96, range = 363–731) and 525 ms long (*SD* = 145, range = 313–931) in the non-predictable condition. The predictive window (i.e. the interval between verb onset and target word onset on the predictable experimental trials) was on average 2598 ms long (*SD* = 218, range = 2131–2922).

The participants’ task was to press the appropriate button on their button box (left-hand button for left-hand object on screen, right-hand button on screen) as soon as they knew which object would be referred to. Their button press terminated the trial. The inter-trial interval was 1000 ms. The test consisted of 40 experimental trials, 20 of which were predictable and 20 were non-predictable. Experimental trials were preceded by two (one predictable, one non-predictable) practice trials. The onset of the spoken sentence was timed such that participants had three seconds to preview the two objects prior to the onset of the verb in the spoken sentence. Response latencies were calculated as the difference between the onset of the target word in the spoken sentence and the participant’s button press. A negative value thus reflected a button press before target word onset. The performance indicator was the average RT based on correct responses in the predictable condition.

*33. Monitoring in noise in sentences*. This sentence comprehension test was similar to the word-level counterpart (25. Monitoring in noise in lists). However, rather than monitoring for words or non-words in lists, participants monitored sentences for words that were predictable or non-predictable from the sentence context.

The test consisted of three practice and 60 experimental trials. On each trial, participants heard a word cue presented in the clear, followed by a sentence presented in stationary speech-shaped background noise. As in the other monitoring tasks, the SNR decreased from trial to trial in steps of two, starting at an SNR of 0 until −18 dB. Each SNR was repeated six times (i.e., six rounds of going from SNR 0 to −18 dB). Forty of the 60 trials were target-present items, where the cue was part of the sentence. These trials required a button press. The remaining 20 trials were target-absent items and thus did not require a button press. Sentences were selected from the materials previously used by Piai, Roelofs, Rommers & Maris^51^. One to seven words were added to the ends of these sentences such that the target word was never the final word in the sentence. In half of the target-present items, the cue word could be predicted based on the sentence context^51^ (mean cloze probability = 1, SD = 0.02, range 0.93–1.00). Similarly, half of the target-absent trials were predictive of a particular word, which was, however, not the cue word. Target-present and target-absent trials occurred equally often in each SNR. Predictable and non-predictable trials were presented in a pseudo-random order. Recordings were made from a native Dutch speaker in a sound-shielded booth. To avoid voice familiarity effects when listening in noise, we recorded a different speaker than the one who had produced the stimuli for test 25. Monitoring in noise in lists. Cue words were on average 676 ms long (*SD* = 96, range = 399–850); sentences were on average 4460 ms long (*SD* = 595, range = 3201–5586). On target-present trials, cue words occurred after on average 2538 ms (*SD* = 358, range = 1854–3346) and were on average 353 ms (*SD* = 6, range = 234–466) long in the running speech. Speech-shaped noise was added to recordings using Praat^36^. The noise was added over the entire recording.

The trial structure was as follows: Participants pressed the right-hand button on the button box to start the trial. A fixation cross appeared on the screen and the cue word was played back (in the clear). After a 500 ms interval, the sentence was played back (in speech-shaped background noise). Participants were instructed to press the right-hand button on their buttonbox when detecting the cue word in the sentence. Upon pressing the button, the fixation cross disappeared, but the sentence presentation continued until the end of the sentence. Button presses were coded as correct when they occurred within the period spanning 300 ms prior to target word onset in the unfolding sentence, during its unfolding, or 500 ms thereafter. The difference between the proportion of correct responses to target-present trials and the proportion of incorrect responses on target-absent trials (false alarms) was calculated for the predictable and the non-predictable condition separately. The performance indicator was operationalized as the ‘predictability benefit’ (i.e., the difference between the correct-incorrect differences in the predictable vs. the non-predictable conditions).

## Data Records

**Fig. 2:**
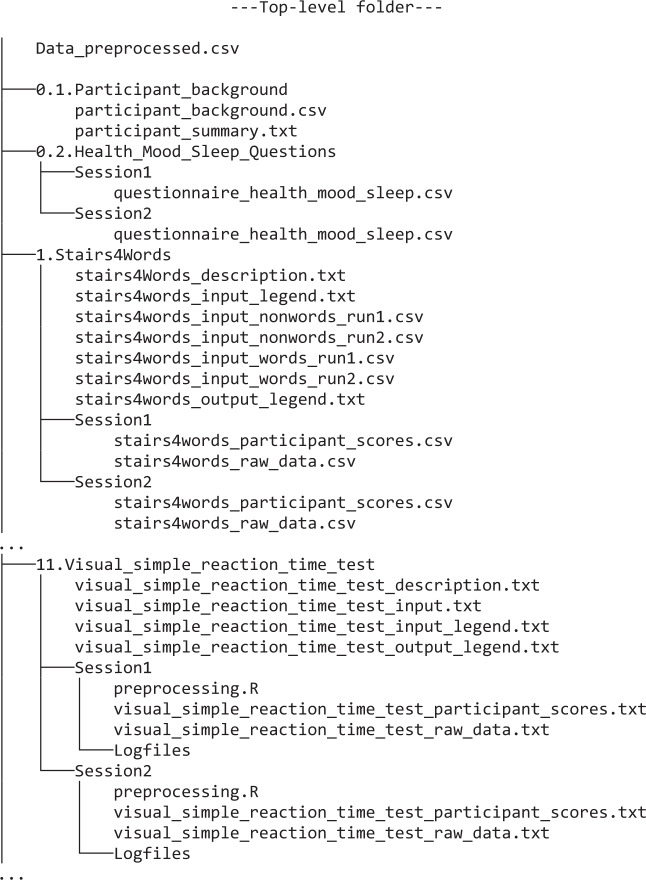
Visualization of the data structure.

The data collection can be accessed at the UKDA^52^. The organization of the data collection is illustrated in Fig. 2. The top-level folder contains 36 sub-folders, a csv file (‘Data_preprocessed.csv’) and docx file. The csv file contains the pre-processed data (one performance indicator per participant, per test, per test day). The docx file contains an overview of the archived data with technical details (e.g., description, size, type) for each folder and file.

The first two sub-folders contain the participant background data collected at the beginning of the first test day (‘0.1.Participant_background’) and participants’ responses to the well-being questions (‘0.2.Health_Mood_Sleep_Questions’) at the beginning of each test day. The remaining 34 folders contain the test data. Note that the data from test 29., although collected in the same application, were divided into two separate folders (29.Phrase_generation and 29.Sentence_generation) for file size reasons and better handling of the data. The enumeration and names are the same as in the test descriptions above. Each test folder contains two sub-folders: ‘Session 1’ (test day 1) and ‘Session 2’ (test day 2), respectively. The test folders additionally contain a ‘description file’ summarizing the materials and procedure of each test, an ‘input’ file, specifying the list and order of stimuli used in the Frinex or Presentation implementation, as well as two ‘legend’ files, providing a key for the column headers in the test input and output files. Note that due to privacy and security reasons we cannot make available the R scripts used to retrieve the raw data from the experiments programmed in Frinex. The reason is that the scripts contain the logins and passwords to the institute web servers.

Moreover, the Session 1 and Session 2 folders each contain a ‘raw_data.txt/csv’ and a ‘participant_scores.txt/csv’ file. While the ‘raw_data’ file contains the raw test output from each participant merged into one file, the ‘participant_scores’ file contains the aggregated performance indicators for the individual participants for a given test. Where applicable, the Session 1 and 2 folders additionally contain Presentation logfiles, audio recordings, Praat annotations and the R script (‘preprocessing.R’) used to pre-process speed-based data (see Online-only Table 1, for an overview of the file types archived for each test).

### Data pre-processing

For the following tests with speed-based performance indicators, we applied a generic pre-processing pipeline^11,53^: 8. Auditory simple RT, 9. Auditory choice RT, 10. Letter comparison test, 11. Visual simple RT, 12. Visual choice RT, 15. Eriksen Flanker test, 18. Picture naming test, 26. Rhyme judgement, 27. Auditory lexical decision, 28. Semantic categorization, 29. Phrase generation, 31. Gender cue activation during sentence comprehension, 32. Verb semantics activation during sentence comprehension. This pre-processing pipeline involved data trimming (stage 1) and outlier replacement (stage 2).

For each test, we first determined upper and lower boundaries for data trimming on the basis of the overall, between-participant RT distributions. Any values below or above these boundaries were replaced with the values of the lower and upper boundaries for that test. The lower and upper boundaries used in the first stage differed from test to test (Table 3, for an overview). Next, the within-participant RT distributions were examined for RTs that were more than 3 SDs away from an individual’s mean RT (in a given condition, if applicable). Except for test 29., these observations were replaced with RTs that were 3 SDs away from that individual’s RT (in a given condition). For 29., this was not possible due to the limited number of items per block. Tests 8. to 12., 18., and 26. to 29. were log-transformed prior to applying the pre-processing pipeline to improve skewness and kurtosis. RTs from tests 31. and 32. could not be log-transformed as they involved negative RTs (responses prior to target word onset).

**Table 3:** Lower and upper boundaries (in milliseconds) for raw data trimming procedure (stage 1) in RT-based tasks.

Test	Upper limit	Lower limit
8. Auditory simple reaction time	100	1000
9. Auditory choice reaction time	200	2000
10. Letter comparison test	300	3000
11. Visual simple reaction time	100	1000
12. Visual choice reaction time	200	2000
15. Eriksen Flanker	200	2000
18. Picture naming test	300	3000
26. Rhyme judgment	300	3000
27. Auditory lexical decision	300	3000
28. Semantic categorization	300	3000
29. Phrase generation	300	3000
31. Gender cue activation during sentence comprehension	−2500	1500
32. Verb semantics activation during sentence comprehension	−2500	1500

Participants’ performance indicators for each test and each test day were merged into the Data_preprocessed.csv file (located in the top-level folder). To facilitate correlation and regression analyses, we reversed the signs for all speed-based performance indicators such that higher scores reflect better performance.

## Technical Validation

Participants were monitored during data acquisition to ensure task compliance and general data quality. Using the data listed in the ‘Data_preprocessed.csv’ file, for each test, we calculated statistical measures of reliability, including skewness, kurtosis, internal consistency, and test-retest reliability (Online-only Table 2). Please see the Usage Notes section for information about missing values.

Inspection of the raw data and descriptive analyses indicated that a serious programming error had occurred in one test (1. Stairs4words), and that two other tests (19. Rapid automatized naming and 7. Syntest) were, respectively, too difficult and too easy. Please see below for further information. We make the data from these tests available to be open and transparent but we strongly discourage others from using them.

(1) 1. Stairs4Words: Due to a programming error, the first session data of 86 of the 112 participants are not usable as the test started with a word sampled from a wrong starting band (40 instead of 20). Thus, the test was too difficult for these participants.

(2) Similarly, for 19. Rapid automatized naming, we calculated performance indicators for participants who had at least one valid run per condition. Applying this criterion would lead to the exclusion of 34 participants, almost one third of the sample.

(3) Finally, descriptive analyses of 7. Syntest revealed close-to-ceiling performance indicating that the test was too easy. Average accuracy was 95%, with a standard deviation of 6% signalling little variability across individuals.

## Usage Notes

The data collection is available at the UKDA. The link to the archive can be found in the References section. The data collection is classified as ‘safeguarded’, which means among others that – in line with the informed consent obtained from the participants – data usage is confined to academic purposes (academic use, attribution required, non-commercial, no redistribution allowed). A login is required for accessing the data collection. When creating the login with UKDA, users have to agree to a generic End-User Agreement (EUA). The files in the collection can be accessed and downloaded in partial or full. The folders within the top-level folder are zipped and need to be unpacked after download.

Below, we list the tests for which data points are missing. Data are either missing because no data were recorded (e.g. due to technical or human failure) or because our analyses revealed a problem in participants’ behaviour. In the latter case, the participants were still listed in the raw_data file, but were excluded from the participant_scores file.

10. Letter comparison test: Due to a technical error, the data of three participants were not recorded in Session 1 (4878981d-5f3e-45f2-9d0d-510f59c5b93d, 9ad6aaea-721c-4b5e-ba36-2308b6799541, cabd74f8-46fe-4986-978f-23231d300ab3). Two participants did not take the task seriously and were therefore excluded (115468d4-8264-4407-8029-0bef987a31e1: accuracy on second test day = 0.27, and a6899552-60eb-45fd-82bc-9f7344bc3156: accuracy on second test day = 0.42).

13. Digit span test: We excluded the test data of two participants. 4a572f71-1305-41e1-a3ee-fd80f922c2c9 had a score of zero on the backward run on both test days. 115468d4-8264-4407-8029-0bef987a31e1 had a score of zero on the backward run on the first test days. The score of zero suggests that they failed to understand the instructions.

14. Corsi block clicking test: Data from five participants had to be excluded as they had a score of zero on the forward (one participant: 0a433879-31ba-41a8-949b-052678732bb6) or backward (four participants: 1dba5c87-132b-4a0a-bc76-3afddd57874a, 4a572f71-1305-41e1-a3ee-fd80f922c2c9, a6899552-60eb-45fd-82bc-9f7344bc3156, c6c6fb8f-9744-4f8d-912a-ed212ab4b5f1) run, suggesting that they failed to understand the task.

15. Eriksen flanker test: Data from six participants were excluded: Three had misunderstood the task (c6c6fb8f-9744-4f8d-912a-ed212ab4b5f1, cabd74f8-46fe-4986-978f-23231d300ab3, 4878981d-5f3e-45f2-9d0d-510f59c5b93d) and three did not do the task seriously (1dba5c87-132b-4a0a-bc76-3afddd57874a: accuracy on second test day = 0.00, ad02c84d-df23-43d5-b7e2-fc659a054fec: accuracy on first test day = 0.5, e235ac1c-0f68-4e8b-8ed1-fdcf61292224: accuracy on second test day = 0.06).

16. Antisaccade test: Data from one participant had to be excluded as this person had a score of zero and thus had failed to understand the instructions (3d87acd7-141e-4ab5-8e0f-00136d473877).

18. Picture naming test: Three items were excluded from all analyses as they had naming accuracies below or close to 50% (passer ‘compasses’ = 43%, punaise ‘pushpin’ = 52%, zeef ‘sieve’ = 48%) on the first test day. Due to a technical problem, no recordings were made for one participant in Session 2 (d1cff5f9-6da2-48c7-8241-f7ed00ae2c7e).

20. Antonym production: One person did not understand the instructions and therefore had to be excluded (1dba5c87-132b-4a0a-bc76-3afddd57874a, session 2); they produced the cue word in reversed order.

21. Verbal fluency: Six participants had to be excluded as they produced zero words in one of the conditions (33cf8ad8-ba25-40cb-bd79-3b6a1a11be40, animals and food & drinks, session 2; 445db31a-9c27-408e-abe2-5bb8671cf860, animals, session 1; 636b4437-7843-4d38-b558-2906e33c0d46, animals, session 1; 69c5e4cd-2a37-4df8-8b9e-7cf13d323ad6, animals, session 1; a6899552-60eb-45fd-82bc-9f7344bc3156, animals and food & drinks, session 2; c9902150-3fbb-4841-a874-34a9d6c0839d, animals and food & drinks, session 2).

22. Maximal speech rate: Six participants had to be excluded as none of their two runs was valid (012d3684-fefb-45e2-b913-3c88d421fe74, both sessions; 0a433879-31ba-41a8-949b-052678732bb6, session 1; 371e6913-3e59-43be-a19d-f7fcbf55e214, both sessions; 8c680230-a201-4792-a729-5832e77cb56a, session 1; a6899552-60eb-45fd-82bc-9f7344bc3156, both sessions; c9902150-3fbb-4841-a874-34a9d6c0839d, both sessions).

23. One-minute-test: One participant was excluded as they did not understand the instructions (a37e9013-4a41-45b9-b8b0-54c9de094e4d, session 2).

24. Klepel test: One participant was excluded as they did not understand the instructions (a37e9013-4a41-45b9-b8b0-54c9de094e4d, both sessions).

25. Monitoring in noise in lists: Three participants misunderstood the meaning monitoring task and had a score of zero (aa1f1b90-a9e0-4f06-bf0f-31f08b4e038a, bb3d5d6c-9700-44f0-b86a-b1b6c04e9a81, d90fb2cd-41dc-4e59-a7fe-92d57dbd0770).

26. Rhyme judgement: Due to a technical problem, the data of three participants were not recorded in Session 1 (4878981d-5f3e-45f2-9d0d-510f59c5b93d, 9ad6aaea-721c-4b5e-ba36-2308b6799541, cabd74f8-46fe-4986-978f-23231d300ab3).

28. Semantic categorization: Due to a technical problem, the data of three participants were not recorded in Session 1 (4878981d-5f3e-45f2-9d0d-510f59c5b93d, 9ad6aaea-721c-4b5e-ba36-2308b6799541, cabd74f8-46fe-4986-978f-23231d300ab3).

31. Gender cue activation during sentence comprehension: Accuracy on the first part was used to filter participants for analysis of the second part. Participants who scored below 80% on the first part were excluded from further analysis.

Two items were excluded from any analyses as less than 50% of participants indicated the correct grammatical gender of these two objects in Part 1. This concerned the distractor picture ‘hippo’ (Dutch: nijlpaard, Session 1: 46%, Session 2: 53%) and the target picture ‘leg’ (Dutch: been, Session 1: 42%, Session 2: 53%).

Additionally, data from seven participants were excluded: Two of them scored below 80% on part 1 (6832c41d-fc68-480d-aae4-3cc150838622, Session 1: 69%, Session 2: 50%; a6899552-60eb-45fd-82bc-9f7344bc3156, Session 1: 68%, Session 2: 59%). Five participants average response times on non-predictable trials that were negative (4a572f71-1305-41e1-a3ee-fd80f922c2c9, Session 1: −17.71, Session 2: −181.62; 115468d4-8264-4407-8029-0bef987a31e1, Session 1: −917.85, Session 2: −1184.22; aa1f1b90-a9e0-4f06-bf0f-31f08b4e038a, Session 1: −10.53, Session 2: 139.77; 1dba5c87-132b-4a0a-bc76-3afddd57874a, Session 1: 378.00, Session 2: −407.35; 768ebb83-482c-4654-bdba-3af6c178bc99, Session 1: 442.25, Session 2: −420.06), suggesting they pressed the button too early (i.e., before target word onset), most likely based on guessing.

## Supplementary information

Supplementary Information

## Data Availability

The custom written code (R scripts) to pre-process the speed-based data is included in the data release.

## Online-only Tables

**Online-only Table1:** Overview of archived file types per test.

Test	Descr. file	Input file	Input legend	Output legend	Merged raw data	Participant scores	Logfiles	Audio recordings	Annotations
1.Stairs4Words*	✓	✓	✓	✓	✓	✓	—	—	—
2.Peabody picture vocabulary test	✓	✓	✓	✓	✓	✓	—	—	—
3.Spelling test	✓	✓	✓	✓	✓	✓	—	—	—
4.Author recognition test	✓	✓	✓	✓	✓	✓	—	—	—
5.Idiom recognition test	✓	✓	✓	✓	✓	✓	—	—	—
6.Prescriptive grammar test	✓	✓	✓	✓	✓	✓	—	—	—
7.Syntest*	✓	✓	✓	✓	✓	✓	—	—	—
8.Auditory simple RT test	✓	✓	✓	✓	✓	✓	✓	—	—
9.Auditory choice RT test	✓	✓	✓	✓	✓	✓	✓	—	—
10.Letter comparison test	✓	✓	✓	✓	✓	✓	✓	—	—
11.Visual simple RT test	✓	✓	✓	✓	✓	✓	✓	—	—
12.Visual choice RT test	✓	✓	✓	✓	✓	✓	✓	—	—
13.Digit span test	✓	✓	✓	✓	✓	✓	—	—	—
14.Corsi block clicking test	✓	✓	✓	✓	✓	✓	—	—	—
15.Eriksen flanker test	✓	✓	✓	✓	✓	✓	✓	—	—
16.Antisaccade test	✓	✓	✓	✓	✓	✓	✓	—	—
17.Raven’s advanced progressive matrices test	✓	✓	✓	✓	✓	✓	—	—	—
18.Picture naming test	✓	✓	✓	✓	✓	✓	✓	✓	✓
19.Rapid automatized naming*	✓	✓	✓	✓	✓	✓	—	✓	—
20.Antonym production	✓	✓	✓	✓	✓	✓	—	✓	—
21.Verbal fluency	✓	—	—	✓	✓	✓	—	✓	—
22.Maximal speech rate	✓	—	—	✓	✓	✓	—	✓	—
23.One-minute test	✓	✓	—	✓	✓	✓	✓	✓	—
24.Klepel test	✓	✓	—	✓	✓	✓	✓	✓	—
25.Monitoring in noise in lists	✓	✓	✓	✓	✓	✓	✓	—	—
26.Rhyme judgment	✓	✓	✓	✓	✓	✓	✓	—	—
27.Auditory Lexical decision	✓	✓	✓	✓	✓	✓	✓	—	—
28.Semantic categorization	✓	✓	✓	✓	✓	✓	✓	—	—
29.Phrase and sentence generation	✓	✓	✓	✓	✓	✓	✓	✓	✓
30.Spontaneous speech	✓	—	—	—	—	—	—	✓	✓
31.Gender cue activation during sentence comprehension	✓	✓	✓	✓	✓	✓	✓	—	—
32.Verb semantics activation during sentence comprehension	✓	✓	✓	✓	✓	✓	✓	—	—
33.Monitoring in noise in sentences	✓	✓	✓	✓	✓	✓	✓	—	—

Note. Data usage is discouraged for tests marked with an asterisk (see Usage Notes section for details).

**Online-only Table 2:** Descriptive statistics for performance indicators of the first test day.

Domain	Test	N	Mean (SD)	Range	Skewness ^*a*^	Kurtosis ^*a*^	Internal consistency	Retest reliability ^*f*^	Errors (%)	Outliers (%)	
										S1	S2
Linguistic Knowledge	2. Peabody picture vocabulary test	112	56 (25)	0–95	−0.43	−0.79	0.96 ^b^	0.91	—	—	
	3. Spelling test	112	0.56 (0.18)	0.1–0.93	−0.43	−0.37	0.83 ^c^	0.85	—	—	
	4. Author recognition test	112	0.2 (0.12)	−0.03–0.6	0.62	0.47	0.93 ^c^	0.95	—	—	
	5. Idiom recognition test	112	0.76 (0.13)	0.4–1	−0.33	0.03	0.53 ^c^	0.78	—	—	
	6. Prescriptive grammar test	112	0.69 (0.13)	0.4–1	0.04	−0.65	0.74 ^c^	0.86	—	—	
General cognitive skills	8. Auditory simple RT test	112	Log: 2.35 (0.08) Raw: 235 (48)	Log: 2.2–2.65 Raw: 160–459	−1.36 ^d^	3.1^d^	0.9 ^de^	0.59 ^d^	—	0.85	1.21 ^d^
	9. Auditory choice RT test	112	Log: 2.6 (0.09) Raw: 417 (100)	Log: 2.41–2.86 Raw: 263–799	−0.6 ^d^	0.15 ^d^	0.96 ^de^	0.76 ^d^	3.75	0.81	0.49 ^d^
	10. Letter comparison test	107	Log: 3.02 (0.08) Raw: 1167 (251)	Log: 2.86–3.28 Raw: 748–2044	0.65 ^d^	0.47 ^d^	0.89 ^de^	0.83 ^d^	6.89	2.01	0.08 ^d^
	11. Visual simple RT test	112	Log: 2.37 (0.05) Raw: 244 (33)	Log: 2.24–2.55 Raw: 179–358	−0.54 ^d^	0.51 ^d^	0.86 ^de^	0.58 ^d^	—	0.49	1.88 ^d^
	12. Visual choice RT test	112	Log: 2.62 (0.07) Raw: 439 (90)	Log: 2.5–2.86 Raw: 321–822	0.88 ^d^	0.73 ^d^	0.95 ^de^	0.78 ^d^	4.13	0.19	0.49 ^d^
	13.Digit span test forward 13. Digit span test backward	112 110	8 (2) 7 (2)	4–13 2–12	0.26 0.22	−0.74 −0.56	0.81 ^c^ 0.72 ^c^	0.75 0.7	— —	— —	
	14. Corsi block clicking test forward 14. Corsi block clicking test backward	111 108	8 (2) 7 (2)	3–12 3–12	−0.08 −0.04	0.25 −0.15	0.53 ^c^ 0.71 ^c^	0.39 0.49	— —	— —	
	15. Eriksen Flanker test	106	Log: 0.09 (0.03) Raw: 101 (56)	Log: −0.06–0.18 Raw: −110–324	−0.57	2.77	0.98 ^e^	0.50	2.67	0.2	0.75 ^d^
	16. Antisaccade test	111	0.89 (0.1)	0.4–1	−2.09	6.4	0.89 ^c^	0.71	—	—	
	17. Raven’s advanced progressive matrices test	112	0.55 (0.17)	0.14–0.89	−0.48	−0.33	0.87 ^c^	0.87	—	—	
Linguistic processing skills	18. Picture naming test	111	Log: 2.95 (0.06) Raw: 923 (120)	Log: 2.74–3.08 Raw: 604–1256	−0.28 ^d^	0.76 ^d^	0.88 ^de^	0.69^d^	6.45	0.03	0.73 ^d^
	20. Antonym production	111	0.72 (0.1)	0.48–0.92	−0.28	−0.34	0.7 ^c^	0.74	—	—	
	21. Verbal fluency categories 21. Verbal fluency phonology	106 112	24 (5) 16 (4)	14–39 3–30	0.04 0.15	−0.18 0.55	— —	0.72 0.71	— —	— —	
	22. Maximal speech rate	106	Log: 3.60 (0.09) Raw: 4028 (854)	Log: 3.39–3.82 Raw: 2458–6650	−0.24	−0.07	—	0.88	—	—	
	23. One—minute test	111	90 (14)	56–116	−0.12	−0.57	0.46 ^c^	0.79	—	—	
	24. Klepel test	111	63 (12)	34–107	0.26	0.6	0.88 ^c^	0.88	—	—	
	25. Monitoring in noise in lists Non—word Word form Semantic	112 112 109	0.58 (0.17) 0.83 (0.12) 0.3 (0.19)	−0.3–0.85 0.25–1 −0.5–0.7	−2.11 −2.47 −1.02	7.87 9.98 2.48	— — —	0.59 0.49 0.53	— — —	— — —	
	26. Rhyme judgment	109	Log: 2.89 (0.08) Raw: 817 (183)	Log: 2.72–3.12 Raw: 530–1435	−0.49 ^d^	0.1 ^d^	0.94 ^de^	0.79 ^d^	3.9	0.69	0.57 ^d^
	27. Auditory Lexical decision	112	Log: 2.93 (0.05) Raw: 884 (115)	Log: 2.84–3.1 Raw: 693–1372	0.61 ^d^	0.72 ^d^	0.97 ^de^	0.69 ^d^	4.46	0.26	0.88 ^d^
	28. Semantic categorization	109	Log: 2.92 (0.06) Raw: 861 (147)	Log: 2.8–3.12 Raw: 645–1454	−0.9 ^d^	0.72 ^d^	0.96 ^de^	0.62 ^d^	3.38	0.56	0.67 ^d^
	Phrase and sentence generation 29. Phrases 29. Sentences	112 112	Log: 2.86 (0.07) Raw: 790 (137) 0.79 (0.19)	Log: 2.71–3.08 Raw: 539–1346 0.17–1.00	0.47 ^d^ −1.14	0.8 ^d^ 0.98	0.82 ^de^ 0.95 ^c^	0.79 ^d^ 0.67	4.32 —	4.95 —	— —
	30. Spontaneous speech	112	—								
	31. Gender cue activation during sentence comprehension	105	−588 (655)	−1674–940	0.45	−0.95	0.88 ^e^	0.88	1.6	0.25	0.82
	32. Verb semantics activation during sentence comprehension	112	−742 (673)	−1701–1041	0.62	−0.72	0.86 ^e^	0.76	0.96	0.65	0.68
	33. Monitoring in noise in sentences	112	0.09 (0.13)	−0.3–0.3	−0.67	0.16	—	0.3	—	—	

Note. See Usage Notes section for missing values in column ‘N’.

The nature of the data in the different tests required the application of different tests of internal consistency (e.g. Guttman’s Lambda-2 coefficient, split-half correlation, ICC 2 coefficient).

Values in S1 and S2 columns indicate the percentage of trials replaced during Stage 1 (trimming) and Stage 2 (outlier replacement) in the pre-processing pipeline.

^a^Calculated based on aggregated performance indicators.

^b^Internal consistency was calculated as Guttman’s Lambda-2 coefficient.

^c^Internal consistency was calculated by adjusting split-half (odd–even) correlations with the Spearman-Brown prophecy formula.

^d^Calculated based on log-transformed values.

^e^Internal consistency was calculated as intra-class correlation coefficient 2 using the ‘psychometric’ package^54^in R^55^.

^f^Test-retest reliability was operationalized as two-tailed Pearson’s correlation between performance on test days 1 and 2.

## References

[1] Levelt, W. J. M. *A History of Psycholinguistics: The Pre-Chomskyan Era*. (Oxford University Press, 2013).

[2] Andrews S, Lo S (2012). Not all skilled readers have cracked the code: Individual differences in masked form priming. J. Exp. Psychol. Learn. Mem. Cogn..

[3] Dąbrowska E (2018). Experience, aptitude and individual differences in native language ultimate attainment. Cognition.

[4] Engelhardt PE, Nigg JT, Ferreira F (2017). Executive function and intelligence in the resolution of temporary syntactic ambiguity: an individual differences investigation. Q. J. Exp. Psychol..

[5] Welcome SE, Joanisse MF (2014). Individual differences in white matter anatomy predict dissociable components of reading skill in adults. NeuroImage.

[6] Kidd E, Donnelly S, Christiansen MH (2018). Individual Differences in Language Acquisition and Processing. Trends Cogn. Sci..

[7] Jongman SR, Roelofs A, Meyer AS (2015). Sustained attention in language production: An individual differences investigation. Q. J. Exp. Psychol..

[8] Shao Z, Roelofs A, Meyer AS (2012). Sources of individual differences in the speed of naming objects and actions: The contribution of executive control. Q. J. Exp. Psychol..

[9] Huettig F, Janse E (2016). Individual differences in working memory and processing speed predict anticipatory spoken language processing in the visual world. Lang. Cogn. Neurosci..

[10] Hintz F (2020). Shared lexical access processes in speaking and listening? An individual differences study. J. Exp. Psychol. Learn. Mem. Cogn..

[11] Miyake A (2000). The Unity and Diversity of Executive Functions and Their Contributions to Complex ‘Frontal Lobe’ Tasks: A Latent Variable Analysis. Cognit. Psychol..

[12] Keuleers E, Brysbaert M, New B (2010). SUBTLEX-NL: a new measure for Dutch word frequency based on film subtitles. Behav. Res. Methods.

[13] Keuleers E, Stevens M, Mandera P, Brysbaert M (2015). Word knowledge in the crowd: Measuring vocabulary size and word prevalence in a massive online experiment. Q. J. Exp. Psychol..

[14] Leek MR (2001). Adaptive procedures in psychophysical research. Percept. Psychophys..

[15] Dunn, L. M. & Dunn, D. *Peabody Picture Vocabulary Test (3rd Edition)*. (American Guidance Service, 1997).

[16] Schlichting, L. *Peabody Picture Vocabulary Test Dutch-III-NL*. (Amsterdam, NL: Harcourt Assessment BV, 2005).

[17] Brysbaert M, Sui L, Dirix N, Hintz F (2020). Dutch Author Recognition. Test. J. Cogn..

[18] Hubers, F., Cucchiarini, C., Strik, H. & Dijkstra, T. Normative Data of Dutch Idiomatic Expressions: Subjective Judgments You Can Bank on. *Front. Psychol*. **10**, (2019). 10.3389/fpsyg.2019.01075 PMC6527779 31139119

[19] Favier, S., Meyer, A. S. & Huettig, F. Investigating the relationship between literacy experience and syntactic processing in spoken language. (in prep.).

[20] Hubers F, Snijders TM, de Hoop H (2016). How the brain processes violations of the grammatical norm: An fMRI study. Brain Lang..

[21] Janssen, N., de Swart, P. J. F., Roelofs, A. P. A., Kessels, R. P. C. & Piai, V. Syntest: A Dutch test for the comprehension of (complex) syntactic structures. (in prep.).

[22] Earles JL, Salthouse TA (1995). Interrelations of age, health, and speed. J. Gerontol. B. Psychol. Sci. Soc. Sci..

[23] Salthouse TA (1996). The processing-speed theory of adult age differences in cognition. Psychol. Rev..

[24] Deary IJ, Liewald D, Nissan J (2011). A free, easy-to-use, computer-based simple and four-choice reaction time programme: The Deary-Liewald reaction time task. Behav. Res. Methods.

[25] Wechsler, D. *WAIS-III (3rd edition)*. (Harcourt Test Publishers, 2004).

[26] Kessels RPC, van den Berg E, Ruis C, Brands AMA (2008). The Backward Span of the Corsi Block-Tapping Task and Its Association With the WAIS-III Digit Span. Assessment.

[27] Chu, M., Meyer, A., Foulkes, L. & Kita, S. Individual differences in frequency and saliency of speech-accompanying gestures: The role of cognitive abilities and empathy. *J. Exp. Psychol. Gen*. **143**, 694 (20130805). 10.1037/a0033861 PMC3970852 23915128

[28] Berch DB, Krikorian R, Huha EM (1998). The Corsi Block-Tapping Task: Methodological and Theoretical Considerations. Brain Cogn..

[29] Vandierendonck A, Kemps E, Fastame MC, Szmalec A (2004). Working memory components of the Corsi blocks task. Br. J. Psychol..

[30] Eriksen CW (1995). The flankers task and response competition: A useful tool for investigating a variety of cognitive problems. Vis. Sel. Atten..

[31] Roberts, R. J., Hager, L. D. & Heron, C. Prefrontal cognitive processes: Working memory and inhibition in the antisaccade task. *J. Exp. Psychol. Gen*. **123**, 374 (19950301).

[32] Raven, J., Raven, J. C. & Court, J. H. *Raven manual section 4: advanced progressive matrices*. (Oxford, UK: Oxford Psychologists Press., 1998).

[33] de Groot F, Koelewijn T, Huettig F, Olivers CNL (2016). A stimulus set of words and pictures matched for visual and semantic similarity. J. Cogn. Psychol..

[34] van Heuven WJB, Mandera P, Keuleers E, Brysbaert M (2014). SUBTLEX-UK: A new and improved word frequency database for British English. Q. J. Exp. Psychol..

[35] Marian, V., Bartolotti, J., Chabal, S. & Shook, A. CLEARPOND: Cross-Linguistic Easy-Access Resource for Phonological and Orthographic Neighborhood Densities. *PLoS ONE* **7**, (2012). 10.1371/journal.pone.0043230 PMC3423352 22916227

[36] Boersma, P. P. G. Praat, a system for doing phonetics by computer (Version 5.1.19) [Computer program]. (2001).

[37] Araújo, S., Huettig, F. & Meyer, A. S. An eyetracking study of the dynamics of lexical processing in dyslexic readers. (subm.).

[38] Mainz N, Shao Z, Brysbaert M, Meyer AS (2017). Vocabulary Knowledge Predicts Lexical Processing: Evidence from a Group of Participants with Diverse Educational Backgrounds. Front. Psychol..

[39] Shao, Z., Janse, E., Visser, K. & Meyer, A. What do verbal fluency tasks measure? Predictors of verbal fluency performance in older adults. *Front. Psychol*. **5**, (2014). 10.3389/fpsyg.2014.00772 PMC4106453 25101034

[40] Brus, B. T. & Voeten, M. J. M. *Een-Minuut-Test*. (Berkhout, 1973).

[41] Van den Bos, K. P., Spelberg, H., Scheepsma, A. & De Vries, J. *De Klepel. Vorm A en B. Een test voor de leesvaardigheid van pseudowoorden. Verantwoording, handleiding, diagnostiek en behandeling*. (Berkhout, 1994).

[42] Kilborn K, Moss H (1996). Word monitoring. Lang. Cogn. Process..

[43] Keuleers E, Brysbaert M (2010). Wuggy: a multilingual pseudoword generator. Behav. Res. Methods.

[44] De Deyne S, Navarro DJ, Storms G (2012). Better explanations of lexical and semantic cognition using networks derived from continued rather than single-word associations. Behav. Res. Methods.

[45] McQueen JM (1993). Rhyme decisions to spoken words and nonwords. Mem. Cognit..

[46] Menenti L, Gierhan SME, Segaert K, Hagoort P (2011). Shared Language: Overlap and Segregation of the Neuronal Infrastructure for Speaking and Listening Revealed by Functional MRI. Psychol. Sci..

[47] Jongman, S. R., Khoe, Y. H. & Hintz, F. Vocabulary Size Influences Spontaneous Speech in Native Language Users: Validating the Use of Automatic Speech Recognition in Individual Differences Research. *Lang. Speech*10.1177/0023830920911079 (2020). 10.1177/0023830920911079 32223517

[48] Snodgrass JG, Vanderwart M (1980). A standardized set of 260 pictures: norms for name agreement, image agreement, familiarity, and visual complexity. J. Exp. Psychol. [Hum. Learn.].

[49] Altmann GTM, Kamide Y (1999). Incremental interpretation at verbs: restricting the domain of subsequent reference. Cognition.

[50] Hintz F, Meyer AS, Huettig F (2017). Predictors of verb-mediated anticipatory eye movements in the visual world. J. Exp. Psychol. Learn. Mem. Cogn..

[51] Piai V, Roelofs A, Rommers J, Maris E (2015). Beta oscillations reflect memory and motor aspects of spoken word production. Hum. Brain Mapp..

[52] Hintz F, Dijkhuis M, van’t Hoff V, McQueen JM, Meyer AS (2020). UK Data Service.

[53] Kim AE, Oines L, Miyake A (2018). Individual differences in verbal working memory underlie a tradeoff between semantic and structural processing difficulty during language comprehension: An ERP investigation. J. Exp. Psychol. Learn. Mem. Cogn..

