# Supplementary material for: A behavioural dataset for studying individual differences in language skills
Source: Sci Data. 2020 Dec 8;7:429. doi: 10.1038/s41597-020-00758-x (PMC7722889; doi:10.1038/s41597-020-00758-x)
Supplement: Supplementary file 1 — Supplementary Information [file 41597_2020_758_MOESM1_ESM.pdf]

Test	Page	Test	Page
0.1 Intake questionnaire	1	**Linguistic processing skills tests**	
**Linguistic experience tests**		*Word production*	
1. Stairs4Words	4	18. Picture naming test	89
2. Peabody picture vocabulary test	71	19. Rapid automatized naming (RAN)	90
3. Spelling test	72	20. Antonym production	91
4. Author recognition test	74	21. Verbal fluency	92
5. Idiom recognition test	78	22. Maximal speech rate	92
6. Prescriptive grammar test	80	23. One-minute-test	93
7. Syntest	83	24. Klepel test	95
**General cognitive skills tests**		*Word comprehension*	
8. Auditory simple reaction time test	83	25. Monitoring in noise in lists	96
9. Auditory choice reaction time test	83	26. Rhyme judgment	102
10. Letter comparison test	83	27. Auditory lexical decision	103
11. Visual simple reaction time test	85	28. Semantic categorization	105
12. Visual choice reaction time test	85	*Sentence production*	
13. Digit span test (forward and backward)	85	29. Phrase and sentence generation	107
14. Corsi block clicking test (forward and backward)	86	30. Spontaneous speech	110
15. Eriksen Flanker test	87	*Sentence comprehension*	
16. Antisaccade test	87	31. Gender cue activation during sentence	111
17. Raven's advanced progressive matrices test	88	comprehension	
		32. Verb semantics activation during sentence	113
		comprehension	
		33. Monitoring in noise in sentences	114

0.1 Intake questionnaire

1. First name: ______________________
2. Last name: ______________________
3. Address
   Street: ______________________ House number: _____________________

   Postal code: ____________________ City/town/village: ______________________
4. Bank account: ______________________
5. ID number : ______________________
6. Email: ______________________
7. Date of birth (dd/mm/yyyy): ______________________
8. Gender: (M/F/other)

9. Country of birth: (choice menu)

   Choice 'Other' → Other, namely: ______________________

   Choice = Netherlands:

   Province of birth: (choice menu)

   Where did you grow up? ______________________

10. What is your native language (language acquired from birth)? (choice menu)

    Choice 'Other' → Other, namely: ______________________

    Choice = Dutch:

    Do you speak a dialect? (yes/no)

    If yes, which one: ______________________

    Choice ≠ Netherlands:

    At which age did you start learning Dutch? ______________________

11. Did you learn two or more languages from birth? (yes/no)

    Yes, namely: (choice menu)

12. Did you learn more languages at a later stage in life (for instance at school or while studying abroad)? (yes/no)

    Yes, namely: 1. choice menu since year: ______________________

    How often do you speak this language?

    choice menu (daily, weekly, monthly, incidental)

    2. choice menu since year: ______________________

    How often do you speak this language?

    choice menu (daily, weekly, monthly, incidental)

    (up until 5 languages)

Other: _______________ since year: ______________________

How often do you speak this language?

choice menu (daily, weekly, monthly, incidental)

13. Are there other languages you use regularly? (yes/no)

    Yes, namely: ______________________

14. What is your highest (completed) education? (choice menu)

    What is the highest (completed) education of your mother? (choice menu)

    What is the highest (completed) education of your father? (choice menu)

15. Are you currently a student? (yes/no)

    Yes: Do you study part-time? (yes/no)

    (if answered no, participants will only see the questions about educational level and educational support; if answered yes, participants will also see the questions about their job)

    Yes: What is your current educational level? (choice menu)

    Please select a sector for your educational program by choosing one of the options. (choice menu)

    Other: ______________________

    Do you receive educational support (for instance because of dyslexia, concentration problems, problems with hearing)? (yes/no)

    If yes, please explain: ______________________

    No:

    Do you currently have a paid job? (yes/no)

    Yes: Please select a sector for your job by choosing one of the options. (choice menu)

    What is the scale of your income? (choice menu)

16. Do you currently play an instrument and/or do you sing (for instance in a choir), or did you do so in the past? (yes/no)

    If yes, for how long? ______________________

17. How many hours per week do you watch Dutch television? ______________________
18. How many hours per week do you watch Dutch series and/or films? ______________________
19. How often do you listen to Dutch radio? ______________________

20. How many hours per week do you read in Dutch? ______________________

21. Do you carry out any of the above activities in (one of) your other languages? (yes/no)

    Yes: How many hours per week do you watch television in (one of your other languages)?
    ____

    How many hours per week do you watch series and/or films in (one of) your other languages?
    ____

    How often do you listen to the radio in (one of) your other languages?
    ____

    How many hours per week do you read in (one of) your other languages?
    ____

22. Do you have problems with your sight? (yes/no)

    Yes: Is your sight corrected using glasses or contact lenses? (yes/no)

23. Are you color blind? (yes/no)

    Yes: Which colors do you have difficulty seeing? ______________________

24. Do you have problems with your hearing? (yes/no)

    Yes: Do you wear a hearing aid? (yes/no)

25. Are you right- or left-handed? (Right/Left/Both)
26. Do you have one or more left-handed first degree family members? (yes/no)

    Yes: Is your father left-handed? (yes/no)

    Is your mother left-handed? (yes/no)

    Do you have a brother and/or sister that is left-handed? (yes/no)

27. Do you have an illness or handicap, which makes it difficult to use the keyboard? (yes/no)
28. Do you have dyslexia? (yes/no)
29. Do you have speech problems (e.g., stuttering)? (yes/no)

    Yes, namely: ______________________

30. Do you have an illness impacting on your concentration and/or causing difficulties with learning (for instance ADHD, ADD or autism)? (yes/no)

    Yes, namely: ______________________

Linguistic experience

1. Stairs4Words

Run 1: Words

Band	Word	Prevalence	Log Google Frequency	Difference Belgium/ Netherlands
1	vaak	1.00	5.26	0.0
1	stoeien	1.00	3.45	0.0
1	snurken	1.00	3.41	0.0
1	wens	1.00	4.43	0.0
1	wurgen	1.00	3.50	0.0
1	leegte	1.00	4.01	0.0
1	klimmen	1.00	3.91	0.0
1	begeleiden	1.00	3.98	0.0
1	belang	1.00	5.09	0.0
1	matig	1.00	3.87	0.0
1	binnen	1.00	5.37	0.0
1	verdwijnen	1.00	4.48	0.0
1	plechtig	1.00	3.84	0.0
1	verdrijven	1.00	3.74	0.0
1	tiener	1.00	3.54	0.0
1	bril	1.00	4.23	0.0
1	traditie	1.00	4.65	0.0
1	geheugen	1.00	4.29	0.0
1	teleurgesteld	1.00	4.04	0.0
1	groep	1.00	5.05	0.0
2	installeren	1.00	3.50	0.0
2	belegging	1.00	3.25	0.0
2	nationaal	1.00	4.73	0.0
2	struik	1.00	3.77	0.0
2	kunstzinnig	1.00	3.42	0.0
2	opwindend	1.00	3.75	0.0
2	stofzuiger	1.00	3.49	0.0
2	poort	1.00	4.23	0.0
2	stiefmoeder	1.00	3.52	0.0
2	onderschatten	1.00	3.60	0.0
2	rommel	1.00	3.87	0.0
2	authentiek	1.00	3.68	0.0
2	mos	1.00	3.85	0.0
2	appeltaart	1.00	3.52	0.0
2	bijzonder	1.00	5.03	0.0
2	sprookje	1.00	3.81	0.0
2	hangmat	1.00	3.40	0.0
2	bemoeien	1.00	3.86	0.0
2	aanvallend	1.00	3.48	0.0
2	deurknop	1.00	3.25	0.0
3	rolschaats	1.00	2.57	-0.5
3	ongeval	1.00	3.67	0.3
3	kaak	1.00	3.85	-0.4

3	snoeien	1.00	3.35	-0.4
3	verbinden	1.00	4.13	-0.5
3	afknapper	1.00	3.24	-0.1
3	voorspoedig	1.00	3.58	0.0
3	bevruchting	1.00	3.34	-0.7
3	blok	1.00	4.29	-0.4
3	knop	1.00	3.93	-0.1
3	omtrek	1.00	3.74	-0.4
3	scheerschuim	1.00	2.98	-0.4
3	gokspel	1.00	2.95	-0.4
3	geestelijk	1.00	4.16	0.0
3	worden	1.00	5.55	0.0
3	nutteloos	1.00	3.68	-0.4
3	bepaald	1.00	4.96	-0.4
3	beeldspraak	1.00	3.69	-0.4
3	zandbak	1.00	3.42	-0.4
3	gelegenheid	1.00	4.82	-0.4
4	vraagstuk	1.00	4.05	-0.1
4	kunstenaar	1.00	4.50	0.3
4	slotvraag	1.00	2.66	-0.1
4	schrikkeljaar	1.00	3.19	-0.4
4	zien	1.00	5.41	-0.4
4	spar	1.00	3.32	0.0
4	fotografisch	1.00	3.40	0.0
4	volwaardig	1.00	3.66	0.3
4	fruit	1.00	4.20	0.0
4	verfpot	1.00	2.53	0.0
4	aanvoeren	1.00	3.50	0.3
4	balkon	1.00	3.90	0.3
4	blazen	1.00	3.94	-0.5
4	alleen	1.00	5.48	-0.5
4	geel	1.00	4.33	0.3
4	eergisteren	1.00	3.53	-0.4
4	gezicht	1.00	5.15	0.4
4	verblijfplaats	1.00	3.75	-0.4
4	wachtlijst	1.00	3.35	-0.1
4	verkeer	1.00	4.46	-0.1
5	toen	1.00	5.14	-0.4
5	liggend	1.00	3.85	-0.5
5	roomijs	1.00	3.27	0.0
5	huurwoning	1.00	3.13	-0.4
5	arresteren	1.00	3.58	-0.1
5	paskamer	1.00	2.67	-0.4
5	viool	1.00	3.76	-0.5
5	zwemster	1.00	3.07	-0.4
5	zelfportret	1.00	3.84	-0.1
5	idioot	1.00	3.90	-0.1
5	afkijken	1.00	3.14	-0.1
5	slapen	1.00	4.63	-0.5
5	onrecht	1.00	3.92	-0.1
5	relatief	1.00	4.63	-0.4
5	fietstocht	1.00	3.44	-0.5
5	ijzerdraad	1.00	3.36	0.0

5	onvolledig	1.00	3.67	-0.1
5	sterven	1.00	4.47	-0.4
5	virus	1.00	3.85	-0.1
5	lef	1.00	3.71	-0.1
6	bedekt	1.00	4.15	-0.4
6	giftig	1.00	3.44	-0.4
6	chronologisch	1.00	3.84	-0.5
6	tonijn	1.00	3.23	-0.1
6	toenadering	1.00	3.76	-0.4
6	ruimdenkend	1.00	3.26	0.3
6	ontketenen	1.00	3.32	-0.5
6	geluk	1.00	4.67	0.3
6	ondertiteling	1.00	3.10	-0.6
6	oogschaduw	1.00	3.10	-0.5
6	compositie	1.00	4.15	-0.5
6	bloempot	1.00	3.29	0.3
6	waarschijnlijk	1.00	5.09	0.0
6	medewerkster	1.00	3.50	0.4
6	verwarmen	1.00	3.39	0.0
6	eerder	1.00	5.19	0.3
6	achtervolger	1.00	3.20	-0.5
6	oplichting	1.00	3.26	-0.4
6	hoofdrol	1.00	3.90	0.3
6	beker	1.00	3.90	0.0
7	missen	1.00	4.29	-0.5
7	gast	1.00	4.12	0.2
7	storen	1.00	3.72	0.7
7	bijspringen	1.00	2.99	0.0
7	trommelen	1.00	3.52	0.6
7	siroop	1.00	3.14	0.6
7	knagen	1.00	3.48	0.4
7	genieten	1.00	4.40	-0.4
7	tas	1.00	4.31	0.0
7	overhandigen	1.00	3.55	0.0
7	portret	1.00	4.44	0.3
7	behoedzaam	1.00	3.81	0.7
7	krab	1.00	3.47	-0.1
7	handbagage	1.00	3.16	-0.6
7	conditie	1.00	3.89	-0.5
7	beperkt	1.00	4.73	-0.1
7	opluchting	1.00	4.02	-0.1
7	samen	1.00	5.23	0.7
7	schurk	1.00	3.58	-0.2
7	grondgebied	1.00	4.08	-0.5
8	verwoesting	1.00	3.68	0.3
8	schaal	1.00	4.50	-0.6
8	ontnemen	1.00	3.56	0.2
8	overbrengen	1.00	3.73	0.3
8	socialist	1.00	3.80	0.2
8	worm	1.00	3.56	0.6
8	ovaal	1.00	3.47	0.3
8	studeren	1.00	4.11	-0.3
8	trillen	1.00	3.86	-0.1

8	bovenbeen	1.00	3.17	-0.4
8	gasleiding	1.00	2.63	0.2
8	hartslag	1.00	3.78	-0.2
8	eigen	1.00	5.34	0.3
8	bijleggen	1.00	3.04	0.6
8	recreatief	1.00	3.26	0.0
8	luxe	1.00	4.16	0.7
8	wandelstok	1.00	3.64	0.3
8	bij	1.00	5.19	0.2
8	dolfijn	1.00	3.57	0.7
8	construeren	1.00	3.54	-0.1
9	verklaren	1.00	4.50	-0.1
9	versie	1.00	4.56	0.7
9	breken	1.00	4.26	-0.7
9	veldtocht	1.00	3.66	0.6
9	tocht	1.00	4.29	0.6
9	deelwoord	1.00	3.32	0.0
9	zonsverduistering	1.00	3.26	0.2
9	adresboek	1.00	3.16	-0.2
9	toewensen	1.00	3.22	-0.6
9	toestel	1.00	3.90	0.2
9	microscoop	1.00	3.40	0.6
9	verkering	1.00	3.48	-0.1
9	gevoeligheid	1.00	3.73	-0.2
9	vissersboot	1.00	3.27	-0.1
9	tafelblad	1.00	3.56	0.2
9	verleden	1.00	4.94	0.2
9	bloedgroep	1.00	3.16	0.3
9	betreffen	1.00	3.98	-0.7
9	slechts	1.00	5.18	0.2
9	prima	1.00	4.46	0.2
10	zelfcontrole	1.00	3.12	-0.1
10	diepte	1.00	4.18	0.3
10	uitpluizen	1.00	3.18	0.1
10	bereiken	1.00	4.68	0.7
10	hemels	1.00	3.89	0.2
10	meetkundig	1.00	2.93	0.7
10	ontvanger	1.00	3.81	0.2
10	verslag	1.00	4.67	-0.2
10	luisteren	1.00	4.45	0.6
10	afsterven	1.00	3.43	0.7
10	opdragen	1.00	3.42	0.6
10	maand	1.00	4.76	0.7
10	wachttoren	1.00	3.40	-0.1
10	fietsbel	1.00	2.98	0.2
10	sterveling	1.00	3.51	-0.1
10	treuzelen	1.00	3.16	-0.5
10	blijkbaar	1.00	4.67	0.3
10	poetsen	1.00	3.71	-0.1
10	geheim	1.00	4.56	0.3
10	treinreis	1.00	3.46	-0.5
11	geladen	1.00	3.96	0.3
11	schelp	1.00	3.64	0.2

11	mislukken	1.00	3.78	-0.1
11	kunstmest	1.00	3.40	-0.7
11	voorschieten	1.00	2.68	0.3
11	buitenschools	1.00	2.67	-0.1
11	ondervragen	1.00	3.48	0.0
11	gereserveerd	1.00	3.87	0.3
11	psycholoog	1.00	3.70	-0.5
11	alvast	1.00	4.08	0.3
11	schildpad	1.00	3.49	-0.6
11	herenhuis	1.00	3.53	-0.2
11	lastpost	1.00	3.34	-0.6
11	roeiboot	1.00	3.54	0.4
11	levensles	1.00	3.31	-0.2
11	decoratie	1.00	3.74	0.7
11	uitglijden	1.00	2.95	0.5
11	menselijkheid	1.00	3.64	0.2
11	atoom	1.00	3.42	-0.4
11	moeite	1.00	4.84	0.6
12	blik	1.00	4.95	0.3
12	stoofvlees	1.00	3.10	0.5
12	splitsing	1.00	3.72	0.2
12	sommige	1.00	4.99	0.2
12	onvervangbaar	1.00	3.30	0.1
12	compleet	1.00	4.37	-0.4
12	versieren	1.00	3.64	0.7
12	dreigbrief	1.00	3.26	-0.1
12	overmatig	1.00	3.42	0.7
12	noordpool	1.00	3.52	0.1
12	stroming	1.00	3.94	0.2
12	begraafplaats	1.00	3.86	-0.1
12	veroveren	1.00	3.91	-0.6
12	overwaaien	1.00	3.16	0.5
12	appelmoes	1.00	3.40	0.7
12	gloednieuw	1.00	3.39	0.2
12	agent	1.00	4.32	0.3
12	dramatisch	1.00	3.89	0.1
12	kunstvorm	1.00	3.42	0.2
12	verzachten	1.00	3.53	0.3
13	doorbrengen	1.00	3.85	-0.3
13	afmeten	1.00	3.15	0.6
13	meevallen	1.00	3.32	-0.2
13	mogelijk	1.00	5.26	0.7
13	sneeuw	1.00	4.37	0.1
13	pier	1.00	3.81	0.3
13	overhouden	1.00	3.38	0.5
13	smal	1.00	3.96	0.6
13	opzwellen	1.00	2.91	0.2
13	inktvis	1.00	3.53	0.3
13	weggeven	1.00	3.27	-0.1
13	paardrijden	1.00	3.52	0.9
13	bevriezen	1.00	3.37	0.6
13	bloedtransfusie	1.00	3.16	0.7
13	bioscoop	1.00	3.80	-0.7

13	aangeslagen	1.00	3.71	0.6
13	mei	1.00	5.08	0.3
13	daglicht	1.00	4.00	0.1
13	weerstaan	1.00	3.75	0.6
13	gewijzigd	1.00	4.16	-0.4
14	senior	1.00	3.91	-0.7
14	fietsband	1.00	2.95	0.6
14	hoogst	1.00	4.20	-0.4
14	knoeien	1.00	3.35	0.2
14	neerleggen	1.00	3.71	0.1
14	malaria	1.00	3.53	-0.3
14	vereiste	1.00	4.08	-0.1
14	kapot	1.00	4.09	-0.3
14	muzikaal	0.99	3.47	-0.9
14	bergruimte	0.99	2.91	0.7
14	inslapen	0.99	3.39	0.8
14	scooter	0.99	3.39	0.6
14	platteland	0.99	4.28	0.5
14	machteloos	0.99	3.83	-0.7
14	bonus	0.99	3.47	-1.0
14	bekroning	0.99	3.67	-0.3
14	schoolbel	0.99	2.74	0.1
14	baksteen	0.99	3.79	0.7
14	hulp	0.99	4.79	1.0
14	opera	0.99	4.26	-0.3
15	wereldwonder	0.99	3.40	0.6
15	allereerste	0.99	3.91	0.2
15	nietszeggend	0.99	3.34	-0.3
15	overwerkt	0.99	3.25	-0.7
15	bevolkt	0.99	3.67	-0.1
15	taal	0.99	5.08	0.1
15	onderkaak	0.99	3.18	0.4
15	verdraagzaam	0.99	3.26	0.6
15	paniekzaaier	0.99	2.61	-0.8
15	wegvoeren	0.99	3.11	-0.4
15	afbijten	0.99	3.18	0.2
15	vogelpoep	0.99	2.86	-0.7
15	majoor	0.99	3.97	-0.4
15	heterdaad	0.99	3.42	0.5
15	hetzelfde	0.99	5.03	0.8
15	onafgebroken	0.99	3.77	-0.4
15	zelfvoldaan	0.99	3.10	-0.7
15	terecht	0.99	4.78	0.5
15	gala	0.99	3.41	0.4
15	gemoedelijk	0.99	3.47	-0.1
16	uitsluiting	0.99	3.90	0.4
16	griezelen	0.99	3.53	0.6
16	krokus	0.99	3.30	0.1
16	schuilhouden	0.99	3.16	-0.7
16	illegaal	0.99	3.70	-0.5
16	goddelijk	0.99	3.66	-0.1
16	klinisch	0.99	3.57	-0.8
16	pieken	0.99	3.53	-0.5

16	reporter	0.99	3.43	0.5
16	bovenop	0.99	4.01	-0.4
16	materieel	0.99	3.94	0.6
16	kwaad	0.99	4.65	0.6
16	vaarwel	0.99	3.76	0.1
16	aandoenlijk	0.99	3.62	0.8
16	handel	0.99	4.76	0.5
16	kei	0.99	3.63	0.5
16	verduidelijken	0.99	3.60	0.4
16	spelvorm	0.99	3.05	0.0
16	doorprikken	0.99	3.25	0.9
16	ingetogen	0.99	3.63	0.2
17	schouwburg	0.99	3.91	-0.9
17	zeeleeuw	0.99	3.06	0.1
17	douche	0.99	3.96	0.9
17	pauzeren	0.99	3.20	0.5
17	staatsgrens	0.99	2.74	0.2
17	handhaven	0.99	4.18	-0.1
17	betasten	0.99	3.30	-0.4
17	index	0.99	4.60	-0.2
17	triest	0.99	3.74	0.2
17	levensduur	0.99	3.48	0.7
17	continentaal	0.99	3.34	-1.0
17	baviaan	0.99	3.18	1.0
17	overtrekken	0.99	3.17	0.5
17	draad	0.99	4.15	0.4
17	aandurven	0.99	3.34	-0.1
17	oceaan	0.99	4.11	1.1
17	optie	0.99	4.06	0.5
17	onbevestigd	0.99	2.20	-0.3
17	biljart	0.99	3.27	-0.4
17	disco	0.99	3.42	0.1
18	uitwijken	0.99	3.36	1.4
18	boter	0.99	4.04	0.7
18	luisterspel	0.99	3.38	0.3
18	taille	0.99	3.72	-0.5
18	trakteren	0.99	3.38	-0.4
18	coma	0.99	3.58	-1.5
18	draaibaar	0.99	2.96	-0.2
18	tevergeefs	0.99	4.02	1.3
18	kauwgom	0.99	3.39	0.3
18	voortijdig	0.99	3.68	-0.4
18	perforator	0.99	2.82	0.5
18	bemiddelaar	0.99	3.55	0.9
18	psychiatrisch	0.99	3.51	0.6
18	stilstaan	0.99	3.84	0.4
18	achttien	0.99	4.14	-1.1
18	rugslag	0.99	2.35	-0.4
18	afscheiden	0.99	3.20	1.5
18	miauwen	0.99	2.81	-0.4
18	opdagen	0.99	3.80	-0.7
18	opstopping	0.99	2.70	-1.5
19	verprutsen	0.99	2.77	-0.1

19	ondervoed	0.99	3.18	0.6
19	drijfnat	0.99	3.11	-0.5
19	verdrinking	0.99	3.53	1.6
19	surrealisme	0.99	3.47	-1.4
19	perfectioneren	0.99	3.23	1.3
19	spray	0.99	3.12	-0.5
19	pluizig	0.99	3.21	1.2
19	regisseur	0.99	3.88	0.1
19	nachtelijk	0.99	3.62	-0.2
19	waarin	0.99	5.27	0.1
19	passer	0.99	4.00	-0.6
19	strekkend	0.99	2.85	-1.0
19	berglucht	0.99	2.69	0.0
19	vuistslag	0.99	3.34	-0.5
19	dubbele	0.99	4.44	0.7
19	uithollen	0.99	2.96	-0.9
19	oogmasker	0.99	2.45	0.6
19	uitrukken	0.99	2.98	0.2
19	verloten	0.99	3.18	-1.1
20	manicure	0.99	3.02	1.2
20	noordelijk	0.99	3.86	0.7
20	wegscheren	0.99	2.02	-0.8
20	links	0.99	4.80	0.7
20	golfspel	0.99	2.42	-0.3
20	friemelen	0.99	3.01	-1.4
20	naroepen	0.99	2.44	0.5
20	satelliet	0.99	3.36	-1.3
20	omkieperen	0.99	2.05	0.3
20	ravijn	0.99	3.62	-1.0
20	verfrommelen	0.99	2.35	-0.8
20	redenering	0.99	3.93	0.0
20	afhangen	0.99	3.60	0.1
20	inhuldigen	0.99	2.40	1.2
20	mengbak	0.99	0.60	-0.4
20	wegglijden	0.99	3.21	1.7
20	oogvocht	0.99	1.91	1.1
20	herleiden	0.99	3.57	1.0
20	parfum	0.99	3.66	0.9
20	binnenste	0.99	3.84	0.9
21	besef	0.99	4.42	1.9
21	fotokopie	0.99	3.47	-0.8
21	consequent	0.99	3.95	-0.4
21	ongeliefd	0.99	2.57	0.7
21	uitmoorden	0.99	3.09	0.0
21	getik	0.99	3.33	-1.1
21	everzwijn	0.99	3.39	1.1
21	sturing	0.99	3.58	0.5
21	suggereren	0.99	3.87	-1.1
21	zitbank	0.99	2.87	1.1
21	rabarber	0.99	3.23	-1.0
21	naad	0.99	3.32	-1.3
21	slimmerd	0.99	2.52	0.5
21	kantlijn	0.99	3.49	1.4

21	evenveel	0.99	4.06	-0.8
21	wantrouwig	0.99	3.47	1.5
21	overig	0.99	3.85	0.1
21	eigenares	0.99	3.50	0.4
21	achterhaald	0.99	3.79	1.9
21	onverwachts	0.99	3.72	-1.0
22	hooggespannen	0.99	3.41	-1.3
22	inscannen	0.99	2.85	0.2
22	beweeglijk	0.99	3.35	-0.9
22	houtvuur	0.99	3.07	0.9
22	reukloos	0.99	2.61	-1.2
22	gekneusd	0.99	3.28	0.1
22	wederzien	0.99	3.03	0.8
22	spelmaker	0.99	2.11	-0.2
22	koffiekoek	0.99	2.04	1.4
22	eindstuk	0.99	2.40	-0.3
22	mensachtig	0.99	2.59	-0.8
22	ontsnapping	0.99	3.75	2.0
22	binnenlaten	0.99	3.19	1.7
22	onbeweeglijk	0.99	3.56	1.0
22	stembrief	0.99	1.76	1.6
22	zomerzon	0.99	3.58	-1.3
22	basisteam	0.99	2.17	1.1
22	meerekenen	0.99	2.79	-0.5
22	brulaap	0.99	3.71	-0.2
22	gestoei	0.99	2.72	1.3
23	tekeer	0.98	3.64	-1.4
23	attribuut	0.98	3.38	-0.4
23	atleet	0.98	3.25	-0.2
23	baksel	0.98	2.92	-1.8
23	degraderen	0.98	3.00	-0.4
23	terugvliegen	0.98	2.52	0.0
23	gewelddadig	0.98	3.63	-0.5
23	uiensaus	0.98	2.11	-0.3
23	verven	0.98	3.52	1.7
23	cabine	0.98	3.46	-2.2
23	rondgaan	0.98	3.28	1.5
23	festijn	0.98	3.26	-0.5
23	schermer	0.98	3.56	-0.9
23	grenslijn	0.98	2.97	0.8
23	uitlezen	0.98	3.27	1.1
23	genre	0.98	4.43	1.6
23	oefenpop	0.98	0.85	1.2
23	stamlid	0.98	2.50	0.0
23	inplannen	0.98	2.97	0.7
23	maagholte	0.98	0.90	0.1
24	hendel	0.98	3.28	-0.7
24	onverslagen	0.98	2.36	0.9
24	toereiken	0.98	2.13	-1.0
24	muildier	0.98	2.74	1.4
24	erfgrond	0.98	2.16	-1.0
24	censuur	0.98	3.75	-0.3
24	topduel	0.98	0.30	-0.5

24	plaatsmaken	0.98	3.24	-0.2
24	steppe	0.98	3.63	-2.6
24	doorsteek	0.98	2.69	0.9
24	nonchalant	0.98	3.71	2.3
24	omspringen	0.98	3.24	0.1
24	opklimmen	0.98	3.22	1.8
24	waterpoel	0.98	2.85	0.4
24	minimalistisch	0.98	3.31	0.8
24	pixel	0.98	3.26	-1.5
24	uitbuiter	0.98	2.49	1.2
24	ongelooflijk	0.98	3.98	0.9
24	naturalisme	0.98	3.58	-0.1
24	kippenpoot	0.98	2.38	-1.5
25	oefenbaan	0.98	1.34	0.0
25	langverwacht	0.98	3.07	0.6
25	mestput	0.98	2.21	0.6
25	moedercel	0.98	1.65	0.7
25	eindsom	0.98	0.95	-2.1
25	gesnauw	0.98	2.16	-0.2
25	afleider	0.98	2.39	0.8
25	ingezoomd	0.98	3.21	2.5
25	heenrit	0.98	1.56	0.1
25	inwijden	0.98	3.21	-0.4
25	fitnessen	0.98	2.92	1.4
25	loopwagen	0.98	2.39	1.5
25	disfunctioneel	0.98	3.00	0.3
25	uitprinten	0.98	2.96	-0.2
25	instaan	0.98	3.40	1.6
25	stijldans	0.98	2.21	1.2
25	conceptueel	0.98	3.45	2.1
25	visdraad	0.98	2.71	0.6
25	procentteken	0.98	1.00	-1.8
25	appartement	0.98	4.16	0.6
26	landdier	0.98	2.35	0.7
26	vlakheid	0.98	2.89	0.3
26	voeder	0.98	3.37	-0.9
26	leeskaart	0.98	2.67	-1.8
26	mozzarella	0.98	3.00	1.1
26	hernoemen	0.98	2.13	0.2
26	galopperen	0.98	3.14	-0.9
26	geaarzel	0.98	2.07	-1.0
26	immigreren	0.98	2.56	-0.6
26	irrigatie	0.98	3.12	1.1
26	instructief	0.98	3.27	0.5
26	ongeboeid	0.98	1.74	0.8
26	graffiti	0.97	3.36	-1.8
26	bezitting	0.97	3.15	-1.1
26	geestigheid	0.97	3.29	1.5
26	krijsen	0.97	3.56	2.0
26	weegbaar	0.97	1.57	-0.5
26	stormlopen	0.97	2.16	3.2
26	professor	0.97	4.44	2.7
26	ledemaat	0.97	2.78	0.0

27	rugsteun	0.97	2.10	-1.2
27	zwemslag	0.97	2.46	-1.3
27	verlener	0.97	2.33	0.8
27	spaarcent	0.97	0.30	1.8
27	polariteit	0.97	3.16	2.6
27	instuderen	0.97	3.21	2.9
27	klutsen	0.97	2.57	0.2
27	vastliggen	0.97	2.94	0.9
27	bestanddeel	0.97	3.52	0.7
27	psychopathisch	0.97	2.44	1.5
27	aanreiken	0.97	3.37	-0.6
27	ongekeurd	0.97	0.85	-1.7
27	aanheffen	0.97	2.98	0.1
27	flirterig	0.97	2.71	2.2
27	oncomfortabel	0.97	2.97	0.7
27	afwending	0.97	2.67	-1.7
27	toegeeflijk	0.97	3.06	1.7
27	zelfvoldoening	0.97	2.35	-0.3
27	metabolisme	0.97	3.02	3.0
27	omroeren	0.97	2.19	2.4
28	inhalatie	0.97	2.50	-3.4
28	emigrante	0.97	3.72	-2.0
28	onstuimig	0.97	3.64	-1.9
28	onbeloond	0.97	2.11	1.3
28	concurreren	0.97	3.59	1.8
28	dennenappel	0.97	2.84	-2.9
28	vuilmaken	0.97	2.74	-1.3
28	gedachteloos	0.97	3.48	-2.3
28	daarentegen	0.97	4.62	2.3
28	aflosser	0.97	1.41	2.3
28	veehoeder	0.97	2.43	1.7
28	opdeling	0.97	3.40	1.2
28	immuun	0.97	3.37	0.1
28	loshaken	0.97	2.00	1.1
28	neerdalen	0.97	3.24	2.7
28	toevoeren	0.97	2.10	-2.7
28	invoegsel	0.96	1.00	3.7
28	verhoorder	0.96	2.42	-2.3
28	aanhaling	0.96	3.17	1.9
28	gashendel	0.96	2.40	0.9
29	betuiging	0.96	2.94	1.6
29	heerszuchtig	0.96	2.80	0.3
29	tennisnet	0.96	0.78	4.1
29	reflectief	0.96	3.08	0.0
29	openduwen	0.96	2.24	0.7
29	vleien	0.96	3.18	0.9
29	basket	0.96	3.24	4.3
29	chagrijnig	0.96	3.51	2.5
29	irriterend	0.96	2.85	3.5
29	wervelen	0.96	3.00	-0.9
29	afspringen	0.96	2.80	3.2
29	smoking	0.96	3.42	2.4
29	naspreken	0.96	2.40	-0.6

29	ongelaagd	0.96	0.70	-1.9
29	liaan	0.96	2.75	-1.3
29	kieuw	0.96	3.18	-0.6
29	ongewogen	0.96	2.48	-2.5
29	boomzaag	0.96	1.85	3.9
29	zwever	0.96	2.74	-0.2
29	continu	0.96	3.89	3.6
30	ongebogen	0.96	1.97	0.3
30	goedwillig	0.96	2.25	-0.6
30	recessief	0.96	2.41	-2.8
30	hoofdwet	0.96	2.50	-1.1
30	mascarpone	0.96	2.78	1.1
30	zeepschuim	0.96	2.14	1.4
30	leerpunt	0.96	2.78	-1.2
30	denkkader	0.96	3.24	2.8
30	ontwaking	0.96	2.93	-1.4
30	nazingen	0.96	2.62	0.4
30	formatteren	0.96	2.16	1.4
30	filtratie	0.95	2.39	-1.2
30	knelling	0.95	1.88	-0.1
30	betreding	0.95	2.61	1.1
30	huisgevel	0.95	2.13	3.1
30	opdweilen	0.95	2.27	-1.1
30	anekdotisch	0.95	3.40	-0.1
30	douchegel	0.95	2.94	2.8
30	pianospelen	0.95	3.37	-2.9
30	intypen	0.95	3.02	-0.4
31	jaargetij	0.95	2.76	-0.7
31	halsreikend	0.95	2.82	0.8
31	afzakking	0.95	0.48	-1.8
31	skioord	0.95	3.00	1.9
31	stukmaken	0.95	2.71	1.9
31	inning	0.95	3.51	1.6
31	netvormig	0.95	1.92	0.7
31	toeleven	0.95	2.74	-2.2
31	afkoping	0.95	1.73	-3.0
31	extensief	0.95	3.02	3.1
31	africhter	0.95	1.91	3.8
31	applaudisseren	0.95	3.08	-4.4
31	rempedaal	0.95	2.47	3.7
31	lokduif	0.95	0.48	1.9
31	hoogstand	0.95	2.43	-0.1
31	agressieveling	0.95	2.21	4.4
31	herbivoor	0.95	2.34	0.1
31	poederig	0.94	2.11	3.2
31	opstuwen	0.94	2.57	2.4
31	bibliografie	0.94	4.51	5.2
32	blubberen	0.94	1.72	-2.5
32	voorband	0.94	2.45	-3.7
32	danskoord	0.94	0.90	4.3
32	adellijk	0.94	3.50	-3.7
32	jachtbuit	0.94	2.50	2.9
32	stoppelhaar	0.94	1.81	1.5

32	gespook	0.94	2.30	-5.7
32	watervlak	0.94	2.76	3.5
32	afslijten	0.94	2.38	4.0
32	verstoteling	0.94	2.34	4.4
32	bijwoordelijk	0.94	2.31	1.7
32	omranding	0.94	2.42	-0.6
32	afknagen	0.94	2.00	3.5
32	verbitteren	0.94	2.44	2.1
32	tegenin	0.94	3.35	0.8
32	pasgetrouwd	0.94	3.08	-3.1
32	diezelfde	0.94	4.38	2.6
32	aftreding	0.94	2.31	1.1
32	fluwelig	0.94	2.63	-4.3
32	hondenren	0.94	1.88	2.6
33	onnoembaar	0.93	2.64	6.5
33	wrangheid	0.93	2.69	2.5
33	hoogteligging	0.93	2.40	5.6
33	afdruksel	0.93	0.90	2.1
33	buigpunt	0.93	1.63	1.9
33	desintegratie	0.93	3.41	5.2
33	avonduur	0.93	2.68	-6.7
33	militarisme	0.93	3.29	3.4
33	afkapping	0.93	2.15	2.7
33	afvoering	0.93	2.07	0.5
33	inkomst	0.93	3.19	2.9
33	glansloos	0.93	2.56	5.8
33	daartegenover	0.93	3.72	4.8
33	opmeting	0.93	2.85	6.9
33	sensorisch	0.93	2.65	-4.5
33	jeukerig	0.93	1.86	-3.2
33	overmand	0.93	3.67	-1.3
33	pyromanie	0.93	2.08	5.3
33	stereotypering	0.93	3.20	-0.6
33	verdergaan	0.93	3.41	5.7
34	gezagsvol	0.92	0.90	3.8
34	volbouwen	0.92	2.66	-4.8
34	flatteren	0.92	2.49	3.1
34	martelarij	0.92	0.85	4.4
34	ingeroest	0.92	1.58	0.5
34	halsader	0.92	1.78	-4.8
34	symbolist	0.92	3.33	-0.3
34	onderbenut	0.92	2.50	3.8
34	pronker	0.92	3.02	2.9
34	verreikend	0.92	2.44	1.9
34	reglement	0.92	3.98	7.1
34	trotsheid	0.92	2.43	1.3
34	scepticisme	0.92	3.37	5.1
34	kluisteren	0.92	3.08	-0.5
34	noodcel	0.92	0.00	2.1
34	sisklank	0.92	1.95	4.6
34	collecteur	0.91	2.75	0.6
34	ineengedrongen	0.91	2.29	-0.7
34	nawinter	0.91	2.03	1.2

34	hiertussen	0.91	3.12	2.1
35	convergeren	0.91	2.97	4.8
35	aanstotelijk	0.91	2.34	3.9
35	introversie	0.91	2.85	6.7
35	makheid	0.91	1.82	-0.9
35	inzetting	0.91	2.59	0.5
35	stoeierij	0.91	0.48	-7.5
35	daarvandaan	0.91	3.46	-1.3
35	langzaamaan	0.91	3.53	2.7
35	liefelijk	0.91	3.19	-1.5
35	zeegolf	0.91	2.26	2.3
35	tembaar	0.91	0.78	-0.2
35	bejagen	0.91	2.57	-4.1
35	kwadrateren	0.91	2.01	-0.9
35	uitpeuteren	0.90	1.62	3.7
35	vanglijn	0.90	1.89	6.2
35	omhelsd	0.90	3.39	2.2
35	binnenhuis	0.90	3.50	-2.9
35	gerook	0.90	2.04	-4.1
35	plooisel	0.90	0.85	2.3
35	vraagzin	0.90	2.57	7.5
36	verdrogen	0.90	2.87	-7.8
36	majoriteit	0.90	1.73	4.9
36	baatzucht	0.90	2.45	-0.9
36	doodsvonnis	0.90	2.88	4.1
36	bladerig	0.90	0.60	3.6
36	smaden	0.90	2.55	-0.8
36	hakkelig	0.90	2.20	-7.8
36	aanstuwen	0.90	0.78	5.2
36	volmaking	0.90	2.92	0.7
36	oliegas	0.89	0.70	-0.7
36	doelnet	0.89	0.00	2.8
36	verstrakken	0.89	2.77	7.1
36	kladderig	0.89	0.60	-6.1
36	epidemiologie	0.89	3.29	0.9
36	propeller	0.89	2.95	5.7
36	uitdoving	0.89	2.45	7.8
36	voorfilm	0.89	2.51	-1.4
36	aanvulsel	0.89	0.48	2.7
36	onromantisch	0.89	2.51	0.7
36	anatomist	0.89	2.64	3.2
37	uitlegger	0.88	2.98	-2.2
37	wikkeling	0.88	3.17	-0.2
37	veldrat	0.88	0.78	1.9
37	bidder	0.88	3.04	-4.4
37	tenietgaan	0.88	2.84	2.7
37	gescherm	0.88	1.81	-2.0
37	boekrol	0.88	3.10	3.4
37	veldnaam	0.88	2.40	4.5
37	insluizen	0.88	0.30	-4.7
37	linkerrij	0.88	1.66	0.8
37	standaardisatie	0.88	3.30	-5.0
37	schraalte	0.88	1.97	0.1

37	xenograaf	0.88	0.30	3.5
37	cilindrisch	0.87	2.72	-1.2
37	afsteek	0.87	1.79	-2.7
37	halftij	0.87	0.78	-1.5
37	ertussenin	0.87	3.29	-0.4
37	dialectiek	0.87	3.48	1.2
37	wegpraten	0.87	2.01	2.4
37	klaaghuis	0.87	0.30	0.0
38	antagonistisch	0.87	2.49	3.6
38	admissie	0.87	2.74	3.7
38	drijflaag	0.87	0.60	-1.3
38	vraagpunt	0.87	2.60	-4.8
38	blijgeestig	0.86	1.00	2.3
38	floepen	0.86	2.59	3.6
38	stamtijd	0.86	0.30	3.2
38	harigheid	0.86	2.27	-1.4
38	nesteling	0.86	2.73	-2.3
38	treinziek	0.86	0.00	5.5
38	lofprijzend	0.86	1.57	3.2
38	ijswinter	0.86	0.30	3.9
38	roesmiddel	0.85	2.45	3.4
38	onberecht	0.85	0.48	1.8
38	zwemluier	0.85	0.85	-0.7
38	graviteit	0.85	1.82	2.8
38	polijster	0.85	2.09	2.3
38	smijter	0.85	2.75	4.1
38	rumoeren	0.85	2.08	-4.9
38	bevanging	0.85	0.70	4.0
39	verwijven	0.85	0.60	-5.7
39	montering	0.85	2.29	-2.0
39	collectivistisch	0.84	2.58	6.1
39	verjaging	0.84	2.38	1.9
39	lengtecirkel	0.84	0.48	-5.0
39	droogten	0.84	1.75	-3.8
39	kielen	0.84	3.08	-1.1
39	duvelen	0.84	2.12	-4.6
39	scheper	0.84	3.21	1.3
39	graveersel	0.84	0.70	3.8
39	oervogel	0.83	2.01	-0.8
39	zeiker	0.83	2.13	6.4
39	exotisme	0.83	3.44	8.3
39	onbegaafd	0.83	1.92	4.9
39	telboek	0.83	3.76	5.1
39	polytheen	0.83	0.95	-4.4
39	aanvoegen	0.83	0.60	-2.4
39	kletserij	0.83	0.48	-4.6
39	aanrennen	0.83	3.05	-0.9
39	bekakken	0.83	0.60	0.3
40	epsilon	0.83	3.26	-2.6
40	structuralist	0.83	2.97	1.2
40	zoekvak	0.83	2.53	6.5
40	werkarm	0.82	0.48	2.9
40	remhendel	0.82	0.85	0.5

40	brouwhuis	0.82	2.51	0.6
40	aanzetsel	0.82	0.70	2.0
40	nestbak	0.82	0.00	5.6
40	versmeden	0.82	0.85	-2.9
40	alreeds	0.82	3.01	-6.3
40	bakwagen	0.81	0.95	3.4
40	visruim	0.81	0.78	4.7
40	strengelen	0.81	2.12	-4.3
40	opbieding	0.81	0.30	3.1
40	afzepen	0.81	0.00	-2.3
40	hefvlak	0.81	0.00	4.9
40	respectief	0.81	1.85	0.3
40	persman	0.81	2.47	2.0
40	meerderwaardig	0.81	1.77	2.6
40	doorwerkt	0.81	3.36	-1.2
41	omstralen	0.80	1.45	-2.8
41	zatheid	0.80	2.16	5.6
41	spokerig	0.80	0.30	1.1
41	omwerking	0.80	3.06	-0.7
41	grommer	0.80	1.77	-4.8
41	preektoon	0.80	2.56	3.5
41	blauwheid	0.80	2.00	-1.1
41	keiachtig	0.79	0.30	-2.7
41	groenheid	0.79	2.33	-5.2
41	benijder	0.79	0.95	-0.4
41	proximaal	0.79	2.36	-6.5
41	oplaaiing	0.79	1.00	2.0
41	paleografie	0.78	3.14	5.4
41	selecteur	0.78	2.37	-5.8
41	loszinnig	0.78	1.81	2.9
41	inwaaien	0.78	1.82	1.5
41	carbolineum	0.78	3.16	-9.1
41	narijden	0.78	0.60	2.7
41	messnee	0.78	0.85	-3.6
41	valkerij	0.78	3.11	2.4
42	tokken	0.78	2.27	4.3
42	snijdsel	0.77	1.70	-6.8
42	spinnig	0.77	0.85	-0.9
42	ecstasy	0.77	3.22	0.8
42	zinsbedrog	0.77	2.82	-5.1
42	rotskunst	0.77	2.31	0.4
42	zaakkundig	0.77	1.81	1.1
42	instulping	0.77	1.65	8.0
42	halfdek	0.77	2.20	-3.4
42	routeren	0.76	1.54	-4.2
42	rationale	0.76	3.40	-1.0
42	xenofilie	0.76	2.16	0.9
42	planterij	0.76	0.90	-3.5
42	repulsie	0.76	1.60	9.7
42	doorgankelijk	0.76	1.88	5.8
42	rioleren	0.76	0.30	-3.2
42	dedicatie	0.76	2.82	9.9
42	chromen	0.75	2.69	-8.6

42	doldriftig	0.75	2.03	3.6
42	statica	0.75	2.85	-1.1
43	boogarm	0.74	0.00	1.1
43	periculeus	0.74	1.90	3.8
43	zuursel	0.74	1.85	-1.6
43	depreciatie	0.74	2.86	7.9
43	verderfenis	0.74	0.90	-0.9
43	traverseren	0.74	1.00	10.1
43	plagiator	0.74	2.47	0.2
43	wasvat	0.74	1.67	4.5
43	vistrip	0.74	0.60	-3.0
43	woeling	0.74	2.54	2.5
43	reticulair	0.73	1.88	1.3
43	notulant	0.73	1.94	-4.5
43	conciliatie	0.73	2.71	3.7
43	zandkop	0.73	0.48	1.6
43	kwallig	0.73	1.00	0.8
43	prothesis	0.72	2.24	6.3
43	hymnisch	0.72	2.35	-3.4
43	titelen	0.72	2.06	1.1
43	idiomatisch	0.72	2.67	2.2
43	keisteen	0.71	2.35	-2.6
44	bezemklas	0.71	1.94	-3.7
44	codeur	0.71	2.02	2.4
44	ijsbeen	0.70	0.60	-8.0
44	uitham	0.70	2.36	0.0
44	uitkeping	0.70	0.00	9.3
44	onberijmd	0.70	0.85	-6.9
44	witoog	0.69	2.25	-4.3
44	snottig	0.69	0.30	5.6
44	subsidiariteit	0.69	3.25	-4.6
44	uitvangen	0.69	0.60	-1.1
44	stribbeling	0.69	0.70	4.0
44	spikkelen	0.69	0.78	2.5
44	gloeiing	0.69	2.08	10.1
44	endemie	0.69	2.14	2.8
44	afraken	0.69	2.36	2.0
44	humiliatie	0.68	0.30	-1.4
44	zijschip	0.68	1.71	5.9
44	inbuiging	0.68	0.70	2.2
44	aangieten	0.68	0.85	0.8
44	gezwem	0.68	0.00	0.0
45	knevelwet	0.68	0.48	-4.1
45	armengoed	0.68	0.30	-5.9
45	oculeren	0.67	1.91	-7.3
45	cholerisch	0.67	2.71	2.6
45	morrig	0.67	0.60	2.9
45	defectief	0.67	1.76	-3.6
45	omdijken	0.67	0.90	5.8
45	etheen	0.66	2.34	3.4
45	pokkig	0.66	0.60	4.8
45	atomist	0.66	2.33	7.6
45	histologie	0.66	2.99	0.7

45	geschuind	0.66	0.90	0.2
45	plankerig	0.66	0.48	-8.3
45	gruizelen	0.65	0.78	4.2
45	vetblad	0.64	1.91	-1.7
45	blokmaker	0.64	1.75	-0.1
45	hoornuil	0.64	0.70	6.5
45	smelterig	0.64	0.30	6.5
45	pijniger	0.64	0.78	-2.2
45	nazin	0.63	2.24	6.9
46	pervasief	0.63	2.05	5.2
46	volgbod	0.62	0.60	1.8
46	beboomd	0.62	0.85	6.0
46	aspot	0.62	0.95	2.4
46	nagewas	0.62	0.95	3.0
46	wierig	0.61	0.85	-5.2
46	residueel	0.61	1.86	5.4
46	ontlezing	0.61	3.21	-5.4
46	dampaal	0.61	0.30	-1.4
46	uitloving	0.60	1.88	-1.0
46	zegging	0.60	3.21	1.1
46	zakpijp	0.60	1.88	3.5
46	rijzing	0.60	2.36	1.9
46	graduale	0.60	2.79	1.3
46	resumptie	0.60	2.63	-3.0
46	ebstand	0.59	0.60	4.8
46	teelvocht	0.59	0.78	0.2
46	essayeur	0.59	2.31	-3.9
46	auctionaris	0.59	0.30	-4.0
46	uurcirkel	0.59	0.78	-1.8
47	ribbeling	0.58	0.95	7.6
47	grootbek	0.58	1.49	4.3
47	loopkat	0.57	0.85	-3.3
47	wiegeling	0.57	1.95	3.9
47	jaagpaard	0.57	1.00	-6.3
47	binomiaal	0.57	1.98	6.8
47	grovelijk	0.57	2.17	-2.5
47	rondworm	0.57	2.52	0.8
47	syllogisme	0.57	2.70	5.5
47	paaivis	0.56	0.00	2.3
47	perifrase	0.55	2.32	2.7
47	monisme	0.55	3.16	-8.1
47	manisme	0.55	2.16	8.0
47	eoliet	0.55	0.00	3.4
47	pepsine	0.54	2.12	-2.4
47	automatie	0.54	2.55	10.4
47	septiem	0.54	2.32	4.5
47	afwassing	0.54	1.97	-4.8
47	abortief	0.54	2.01	8.3
47	nabeurs	0.54	0.70	7.2
48	verpappen	0.53	0.78	-0.1
48	apoloog	0.53	0.48	2.7
48	gesteeld	0.53	2.54	2.1
48	noppig	0.53	0.30	2.7

48	proponent	0.52	3.14	3.2
48	bijboek	0.52	0.48	3.7
48	uitvalarm	0.51	0.00	1.8
48	filigram	0.51	1.90	0.5
48	brachiaal	0.51	0.70	7.4
48	zeebaak	0.50	0.00	-3.1
48	geblaseerd	0.50	2.18	4.2
48	insolentie	0.50	1.97	9.9
48	plaveiing	0.49	0.30	2.0
48	rekkerig	0.49	0.30	7.1
48	farceren	0.48	0.90	6.1
48	kannenkijker	0.48	0.30	0.7
48	begieren	0.48	0.30	-8.0
48	spinaap	0.48	0.30	7.1
48	releveren	0.48	2.64	8.0
48	valentie	0.48	2.70	3.9
49	minheid	0.47	0.78	-3.0
49	ijsazijn	0.47	1.00	2.5
49	omnevelen	0.47	0.00	6.2
49	dakvoet	0.47	1.96	-5.6
49	betomen	0.46	1.80	2.3
49	atactisch	0.46	0.90	1.1
49	warrelen	0.46	2.35	6.0
49	euvelmoed	0.46	1.72	-1.9
49	prelatuur	0.46	2.46	5.6
49	dolkop	0.46	0.95	2.8
49	antigen	0.45	2.73	7.4
49	apoplexie	0.45	2.22	5.0
49	narcotine	0.45	0.60	3.6
49	eutrofie	0.45	0.00	5.5
49	spiets	0.45	2.09	-1.4
49	titratie	0.44	2.00	4.1
49	autarchie	0.43	1.98	7.8
49	brombas	0.42	0.30	-2.3
49	comorbiditeit	0.42	3.03	5.9
49	erytrocyt	0.42	2.21	3.1
50	altist	0.41	2.48	-9.7
50	convocaat	0.41	1.81	0.7
50	unicaat	0.40	1.93	0.0
50	hematurie	0.40	1.99	2.2
50	assoneren	0.40	1.79	9.6
50	wrikriem	0.40	0.48	0.1
50	endotheel	0.39	2.07	1.4
50	lijmoor	0.39	0.00	6.7
50	deciel	0.38	2.05	-0.7
50	erratisch	0.38	1.56	-6.0
50	abolitie	0.38	2.24	9.8
50	deputaat	0.38	0.78	4.9
50	elysisch	0.37	2.69	-4.6
50	autotypie	0.37	2.72	3.4
50	aasbloem	0.37	0.00	3.7
50	tartaan	0.37	2.07	5.1
50	speelaard	0.37	0.78	-2.1

50	torus	0.36	2.80	6.5
50	ataxie	0.36	2.39	9.6
50	fijnte	0.35	1.86	1.4
51	anomaal	0.33	0.95	4.0
51	optatief	0.33	1.95	-2.7
51	boraat	0.33	0.85	0.7
51	distaal	0.32	2.49	6.9
51	letaal	0.32	2.14	3.2
51	asvaal	0.31	0.00	8.7
51	stagflatie	0.30	2.15	7.7
51	argentaan	0.30	0.30	4.3
51	discant	0.30	2.82	-1.8
51	maroniet	0.29	0.90	8.6
51	zwelkast	0.29	2.29	2.1
51	psalterium	0.29	3.02	6.7
51	idiolect	0.29	2.66	-1.1
51	foneem	0.29	2.67	2.9
51	onderzaat	0.28	0.85	-4.0
51	schrepel	0.28	0.78	-1.3
51	fosfatase	0.28	2.04	5.5
51	braveren	0.27	2.06	6.6
51	gomarist	0.27	1.90	-7.8
51	apostaat	0.27	2.26	1.7
52	thyrsus	0.26	2.06	-2.2
52	rossinant	0.26	1.81	2.3
52	felonie	0.26	1.62	4.5
52	avalist	0.25	0.70	7.9
52	tarok	0.25	2.41	-0.4
52	emfatisch	0.25	2.29	9.2
52	premolaar	0.25	2.24	-0.5
52	bevitten	0.23	1.53	0.5
52	austraal	0.23	1.43	3.9
52	pateen	0.22	2.11	5.4
52	joggelen	0.22	0.30	6.9
52	ergon	0.22	2.70	8.8
52	saffiaan	0.21	0.78	-0.4
52	mariaal	0.21	2.54	9.8
52	camarilla	0.21	2.76	-3.1
52	brozem	0.21	0.85	-4.9
52	meliniet	0.21	0.00	3.5
52	muleta	0.20	2.11	1.1
52	suppliek	0.20	2.22	-1.8
52	samoreus	0.19	0.78	-3.6
53	actinisch	0.18	0.60	3.1
53	galoet	0.18	2.00	-5.5
53	chiliasme	0.18	2.85	2.3
53	agiotage	0.18	0.70	-2.7
53	strictuur	0.18	1.52	-2.8
53	carieus	0.17	1.36	4.3
53	thiofeen	0.16	0.60	4.7
53	aterling	0.15	2.19	-3.3
53	engelin	0.15	2.17	2.1
53	pepton	0.14	0.60	5.1

53	aalgeer	0.14	0.48	1.8
53	spondee	0.13	2.37	1.6
53	bolkaf	0.13	0.30	-0.1
53	smotsen	0.13	0.00	1.9
53	liniment	0.13	1.00	0.5
53	rizofoor	0.13	0.30	-0.6
53	douarie	0.13	1.93	-5.1
53	amfioen	0.13	2.03	0.7
53	fanaal	0.12	2.69	-0.2
53	ritornel	0.11	0.78	4.7
54	galigaan	0.10	1.87	2.0
54	pollevij	0.09	0.00	-2.4
54	teemsen	0.09	0.70	-0.1
54	bekkeneel	0.08	2.36	3.1
54	boerterij	0.08	0.95	-0.9
54	fleren	0.08	1.94	1.9
54	samaar	0.07	3.61	0.6
54	zelateur	0.07	0.48	0.3
54	toetoep	0.06	2.18	0.4
54	babiroesa	0.06	0.70	-2.0
54	ramin	0.06	2.79	1.4
54	badjing	0.05	2.00	-1.4
54	tmesis	0.05	2.57	1.9
54	streks	0.04	0.70	3.7
54	kamsin	0.03	0.30	0.3
54	wimber	0.03	2.91	1.8
54	eemien	0.03	2.30	0.0
54	fijfel	0.03	0.00	1.5
54	twatwa	0.02	0.78	0.5
54	alkanna	0.02	1.76	0.0

Run 1: Non-words

**Non-word**	**% correct Belgium**	**% correct Netherlands**	**Difference Belgium/ Netherlands**
aaldijding	93.9	96.5	-2.7
aandrapt	91.7	92.1	-0.3
aanstildelijk	95.4	92.2	3.1
aantritilijk	97.1	97.7	-0.6
aanvatinen	97.6	96.3	1.2
aarkien	97.1	96.6	0.6
aarmaring	97.4	97.9	-0.4
aartieken	96	96.4	-0.4
aatslijving	94.8	94.8	0
aatsloging	97.8	97.7	0.1
aatspoving	96.6	96.1	0.6
abbeschaagt	96	96.5	-0.5
achtelkeurt	97.6	97.7	-0.1
achteteel	96	96.3	-0.3
adartlijk	97.1	97.4	-0.3
adartuur	91.1	90.4	0.6

aderrief	93.9	91.5	2.4
admedaren	93	95.1	-2.1
afflist	90.3	90	0.3
afkotelijk	94.3	94.3	0
afmoeving	96.6	97.1	-0.5
afwilsen	90.1	93.8	-3.7
akkebruikt	96.7	97	-0.3
akkoevelijk	90.7	95.6	-4.9
allervans	93.4	93.2	0.2
allizelaar	95.8	96.2	-0.4
alsprengen	95.8	93.4	2.4
altantiventie	91.9	93.8	-1.9
amenlaar	93.1	93.9	-0.8
ameping	97	95.2	1.8
amhaalding	96	97	-1
anderfermeren	91.2	90.4	0.8
andrijnlijk	96.2	97.5	-1.2
ankentement	95.4	90.2	5.2
annevirie	94.8	92.6	2.1
antowechie	97.2	97.4	-0.1
anwaad	95.9	96.2	-0.3
appirtalief	95.2	93.6	1.6
archietnist	90.5	92.7	-2.2
arelvlasie	97.8	96.2	1.6
astenuldig	94.8	97.7	-2.9
asterscheid	96	96.8	-0.8
asubukatie	92.8	90.8	2
atser	93.7	93	0.7
atteen	91.6	95.6	-4.1
barrawemaren	97.3	97.5	-0.2
batveeto	97.3	97.6	-0.2
beanggeelden	97.1	97.5	-0.4
bebrijler	96.1	97.7	-1.6
bedeuriging	93	94.2	-1.2
bedijgbaar	92.1	93.5	-1.4
bedoerderen	92.5	95.8	-3.2
begerving	95.2	93	2.2
begolen	93.1	90.3	2.7
beharmes	94.2	93.6	0.7
bekleigen	90.5	92.1	-1.5
beklig	91.5	95.4	-3.9
beknisseerd	93.1	93.1	0
belelden	94.7	94.9	-0.2
belleemheid	92.4	93.5	-1
belongelaar	93.1	95.6	-2.5
bemettalijk	96.6	95.4	1.3
bemieger	95.1	93.1	2.1
bemille	96.7	96.9	-0.2
bemonner	97.2	96.4	0.8
bemuzaarde	91.8	91.1	0.7
beneliaan	91.1	92	-0.8
benild	93.2	95.2	-2.1

benipofaant	93.8	96.7	-3
beoorlaats	95.4	94.3	1
bepegdeld	95.6	95.6	0.1
berdoep	96.5	97.4	-0.9
berduitelijk	94.9	93.1	1.7
berdussen	97.6	94.6	3
bergastenis	95.8	94.4	1.4
bergegen	96.7	97.1	-0.3
bergeinen	91.1	92.3	-1.3
berkerden	96.9	94.5	2.3
berkeuvelijlen	94.2	97	-2.8
berkikken	96.9	95.1	1.7
berklarren	93.1	95.6	-2.5
berklemen	94.6	94.1	0.5
berlongen	97.3	95.4	1.9
bermeerd	94.2	91.9	2.3
bermieken	91.4	94.5	-3.1
bermuiden	90.2	92.9	-2.8
beronming	97.3	97.2	0.1
berschiegen	96.3	96.5	-0.2
berschieven	96.1	96.9	-0.7
berschippend	95.5	95.4	0.1
berspag	95.1	97.6	-2.5
berstaling	92.1	93.8	-1.7
berstessen	96	95.2	0.9
bervloezing	93.7	97	-3.3
berzweffen	97.7	97.3	0.4
beslaren	90.9	93.3	-2.3
beutstigen	96	96.1	-0.1
bevloetedis	95.8	97.9	-2
bevrielten	96	96	-0.1
beziefd	91.2	95.3	-4.1
blacht	96.1	95.8	0.4
blijns	91.9	97.7	-5.8
blomie	93.6	94.8	-1.2
blusto	96.1	97.3	-1.3
boegtaam	91.5	92.7	-1.2
bokenlien	96.9	95.4	1.4
bonspiljet	96.8	94.6	2.2
boppelen	93.9	92.5	1.4
brap	97.8	96.3	1.5
brerine	93.4	96.8	-3.4
brijpen	94.4	93.6	0.7
brirkeling	95.7	97.8	-2.1
broffeteur	95.7	94.5	1.2
brorkelen	93.8	96.9	-3.1
buidendonten	97.5	97.1	0.4
buntesk	90.5	95.3	-4.8
buraak	93.5	95.1	-1.6
caflonsis	92.2	97.4	-5.3
cagstentieel	93	92.4	0.6
cemepisch	91.7	93.8	-2.1

chovibijn	93.6	95.1	-1.5
clormisch	92.2	92.6	-0.4
conbonsaal	90.4	91.6	-1.2
conlipent	90.5	95.1	-4.6
constrimtie	92	90.9	1.1
coptact	97	97.6	-0.6
cordinegen	94.9	95.4	-0.5
cordinu	91.7	91.7	0
creromaren	92.1	92.3	-0.3
crimodirateit	94.8	95	-0.2
criturg	90	93	-3
cunze	97	97	-0.1
curgeef	95.2	97.4	-2.2
dadegantie	93.6	95.2	-1.6
darfortant	95.4	96.5	-1.2
darmakent	95.1	97.1	-2
datsim	95.5	95.9	-0.4
dattelen	90.9	90.8	0.1
dederaar	92.7	92.7	0
deibel	93.2	90.5	2.8
dekeel	94.4	93.8	0.6
dertigeloog	95.5	93.6	2
dierin	94.8	92.5	2.3
dinst	90.6	95.2	-4.5
diowelisch	97.5	96.1	1.4
dipiadier	93.6	93.8	-0.2
dobitoseren	91.8	93.4	-1.7
doeperij	91.5	94.1	-2.5
dolstueel	95.3	93.3	2
dommeratuur	93.5	90.6	2.9
doning	92.1	95.4	-3.3
doolwist	95.2	94.2	0.9
dralk	94.8	95.3	-0.6
drookperrie	96.3	96.4	-0.1
drootsing	94.1	96.4	-2.4
drozing	93.2	91.6	1.6
duskaal	93.3	95.1	-1.8
ebelij	93.5	94.5	-0.9
ebiblom	95.7	97.6	-1.9
echterijn	92.1	92.9	-0.8
eereverniper	97.1	97.3	-0.1
efgactief	97.1	97.6	-0.5
eggenent	91.7	92.7	-1.1
eitelbaafde	97.9	97.7	0.2
ekkrosief	91.2	92.9	-1.6
ekmisch	92.9	94.6	-1.7
ekpillantie	96	96.9	-0.9
ekwarzeren	97.1	97.4	-0.3
elbotomenteel	91.8	92.2	-0.4
emedeel	94	93.6	0.4
enilitaan	90.7	95.8	-5.1
ennilie	92.6	93.8	-1.1

enstubonen	95	95.4	-0.4
entineuk	94.2	95.8	-1.6
erferaan	92.8	93.5	-0.7
erkeur	92.1	93.9	-1.8
erotaad	93.6	91.1	2.5
espoter	90.9	92.2	-1.3
estrevroleur	93.9	90.5	3.4
etdentrowateit	97.3	97.5	-0.2
evijs	95.5	97.8	-2.2
ezaktel	97.1	97.8	-0.7
fagteur	94.9	95.1	-0.2
faredrafie	93.7	95.5	-1.9
fatijd	95.7	97.9	-2.1
feiker	93.2	94.5	-1.3
fibrattaren	92	95.5	-3.6
fivortripie	94.4	95.4	-1.1
floluirijk	97.5	96.8	0.8
flosperen	90	92.3	-2.2
fluleuse	94.2	96.1	-1.8
frarsen	94.5	94.2	0.3
frieperd	92.1	95	-2.9
fudurense	91.3	95.1	-3.8
gattiaal	92.6	92.8	-0.2
geballelijk	94.1	95.9	-1.8
geeslook	91.8	93.2	-1.4
gejorarte	97.4	97.6	-0.1
geldel	92.2	93.4	-1.2
geloordelijk	92.9	95.2	-2.3
gerdrulding	96.8	97.8	-1
gerpierzel	97.5	95.8	1.7
gerplaaien	97.2	97	0.2
gersnokkend	92.7	96.3	-3.6
gersoveling	96.9	95.3	1.6
gerstieding	94.4	94.9	-0.6
gertogelijk	91.3	95.6	-4.3
gesiel	92.5	93.4	-1
geslook	91.4	93.1	-1.7
gewuksvijn	97.4	97.8	-0.5
glarkier	95.4	96.1	-0.6
gloken	95.2	96.4	-1.1
godderijn	92.8	90	2.8
gorm	92.6	94.4	-1.8
grageloog	91.9	94.4	-2.6
grankig	91.9	94.5	-2.6
grapisie	97.5	95.6	1.9
grarenisch	96.4	96	0.4
grarieel	92.8	92.2	0.7
grateusel	92.2	92.1	0.1
grazist	95	91.7	3.3
griktaat	96.9	97.5	-0.6
groegoding	97.2	97.1	0.1
haledak	94.6	94.8	-0.3
halluitend	90.7	95.7	-5

haltoen	93.5	95	-1.5
hamp	90.4	91.7	-1.3
hapiek	92.4	92.3	0.1
hapueel	93.1	92.2	0.9
helvertoling	90.7	90.5	0.2
hijmalaar	90.5	96.2	-5.7
hoemen	92.2	90.9	1.3
hogment	96.7	94.9	1.8
holinge	93.1	93.4	-0.4
honcretie	92.2	93.4	-1.2
howeliteit	94	96.5	-2.5
huistvrij	93.1	91.8	1.2
humaluur	90.9	94.9	-4
hundes	92.7	94	-1.3
ibbens	92.1	93.8	-1.6
idarliteit	90.7	95.4	-4.7
idditent	94	96.1	-2.1
igirabrie	97.8	97.1	0.7
ijkmilly	97.2	97.1	0.2
ijmgarig	95.2	97.5	-2.2
ijseraan	94	95.6	-1.6
iltrentaat	93.2	94.2	-1
infalbaren	95.9	95.6	0.2
injutome	96.2	96.7	-0.5
inlolaan	97.8	96.4	1.4
inophaarbaar	97.3	96	1.3
inschubelijk	92.3	93.5	-1.2
insiraren	96.4	97.7	-1.3
inslaletie	95.8	96	-0.2
inspuimig	90.4	92.8	-2.4
intallinaal	90.1	90.8	-0.7
intundie	92.5	91.6	0.9
inustogen	93.2	95.3	-2.2
jalmis	93.6	96.3	-2.8
jempet	96.1	96.3	-0.2
joming	91.9	96.3	-4.4
jorstifuge	97.6	97.8	-0.2
juweraren	94.9	96.5	-1.5
kamiliaal	91.1	90.8	0.3
kantimaak	94.6	93.7	0.9
kanvelijkheid	95.8	97	-1.3
kapogerie	95	93.4	1.6
karpes	90.1	90.2	0
karzien	93.3	94.7	-1.4
kasisist	90.8	90.5	0.2
kaspeligisch	94.2	93	1.2
kavinentose	91.8	94.1	-2.2
kedelijks	96.3	97.1	-0.8
kedeling	92.9	93.2	-0.4
keima	94.5	95.4	-1
kenselein	95.4	95.3	0
kenvelopen	97.2	97.2	0
kettident	95.6	93.7	1.9

keumen	90.7	90.3	0.4
keumige	93.4	92.3	1.1
kiddermaad	93.3	94.7	-1.4
killenhoos	90.4	94.8	-4.4
kilselen	95.3	96.3	-1
kinterogie	91.8	92.5	-0.7
klal	97.6	97.1	0.5
klechts	96.7	96.9	-0.2
kleffier	92.7	92.2	0.5
kleimig	91.8	95	-3.2
klellig	96.8	96.6	0.1
klondslad	97.8	97	0.8
kluinteloos	90.7	93.2	-2.5
knimmelig	93.2	94.7	-1.6
kobudant	91.1	91.9	-0.8
kofersief	94.7	97.4	-2.7
koluctie	90.6	94.4	-3.9
konbagieren	90.4	96.7	-6.3
kortalon	94.4	90.3	4.1
kosdeeld	94	95	-1
koselaat	93.4	91.9	1.5
kovioen	92.9	93	0
krijterschap	91.7	92.3	-0.7
krogherti	97.4	97	0.4
krosiaal	93.1	95	-1.9
kruffen	91.1	97.7	-6.7
krurf	96.8	96.8	0.1
krurzig	96.9	96.6	0.3
kuchtaan	94.7	97	-2.4
kuvor	95.9	95.3	0.6
kwantonelief	93.9	97.4	-3.5
kwarzend	90.1	93.1	-3
kwiecht	94	93.5	0.5
kwijger	91.2	96.1	-4.9
lacerdelijk	96.9	97.4	-0.5
ladramage	92.6	93.6	-1
lafus	93	95	-2
lagadilistisch	90	92	-2
largelings	95.3	94.9	0.4
larton	92.3	93.4	-1.1
larzieking	97.4	96	1.4
lategatuur	94.1	90.6	3.5
leglaant	97.3	97.8	-0.5
lekredie	94.3	95.4	-1.1
lelomakatie	95.8	94.4	1.3
lepij	92.1	94.3	-2.3
letmiek	92.2	95.8	-3.6
levading	97.8	97.3	0.5
ligeggie	95.5	96.8	-1.3
ligeggisch	95.3	97.3	-2
linkotioneel	94.8	96.3	-1.5
liprema	90.2	92.9	-2.7

lislekening	97.5	97.7	-0.2
locaan	92.1	95.4	-3.3
lokkel	90.3	91.4	-1.1
lommon	94	97.6	-3.7
lopimicaan	90.3	93.3	-3
lorma	90.7	93	-2.3
lunstemoles	97.2	97.1	0.1
lurlerig	95.7	97.7	-2
maafsheid	97.3	96.1	1.1
maarvaloos	95.1	95.4	-0.3
mabaat	92.9	95.5	-2.6
maberet	90	92.9	-2.9
machteteel	93.1	94.4	-1.2
maddibar	96.2	96.7	-0.5
maloen	91.7	92	-0.3
mardij	90.3	93.3	-3
marwetisch	93.4	92.4	1
mavil	95.7	96.2	-0.5
maving	94.4	94.3	0.1
meetdelijk	95.7	97.2	-1.5
meeuwelin	92.4	95.1	-2.8
megelijk	90.1	90.7	-0.6
megels	90.3	90.6	-0.3
mekjen	97.5	97.6	-0.1
mekontaan	92.3	95.2	-3
menetens	96	95.5	0.4
meponteel	95	95.3	-0.3
meunel	96	94.9	1
miaak	94.2	94.8	-0.6
mialaltisch	91.6	90.4	1.2
mienerlijk	90.3	90.2	0.1
miomenten	94.3	94.4	-0.1
mokelijk	91.7	97	-5.4
momp	93.5	96	-2.5
mosmonol	94.4	96.5	-2.1
nakelieur	97.3	96.5	0.9
nakenstitie	95	94.9	0.1
narzesiteit	91.6	93.6	-2
nastrijls	93.8	94.6	-0.8
natalping	90.7	93.7	-3
neemteperlof	97.7	97.9	-0.2
neezat	97.6	97.3	0.3
nenje	97.5	97.7	-0.1
nettiler	96.3	96.3	0
niekeling	91.9	95.6	-3.7
nijtel	91	91.3	-0.4
nogen	92.6	93.2	-0.6
noger	94.5	97.1	-2.6
nojoost	97.6	97	0.6
norgading	94.4	96.3	-1.9
noromares	93.9	91.8	2.1
norpens	95.3	96	-0.7

nostenkie	97	97.6	-0.6
numelnetiek	95.4	96.4	-1
obkatief	93.9	95.7	-1.8
ochterep	97.1	97.4	-0.3
odpesie	92.9	96.1	-3.3
odstuik	96.9	97.4	-0.5
ogernabaring	93.7	95.8	-2.1
ogin	96.1	96.4	-0.2
okdrimaat	94.5	93.5	1
omelvlieden	96	97.9	-1.9
omluiring	94.6	94.5	0.1
ompegeten	96.2	97.7	-1.5
omraarts	96.3	94.8	1.5
omrundend	92.5	94.2	-1.6
onciarettisch	90.7	91.5	-0.7
ongebels	94.6	96.6	-2
onjamming	95.3	96.5	-1.2
onslardelijk	93.8	96.3	-2.5
onsmallen	94.7	94.8	-0.1
onsolig	96.4	96.9	-0.5
onsonbalijk	96.7	97.1	-0.5
onspepen	97.3	97.3	0
ontjazen	96	95.2	0.9
ontplurben	95.1	95.7	-0.6
ontreensmaar	97.5	96	1.5
onvepeilerd	95.4	96	-0.6
oorpoop	96.7	95.3	1.4
ootpeed	97.3	96.7	0.6
opdronentie	90.8	92.5	-1.8
oppumetie	92.4	92.2	0.2
orjetier	92	95.7	-3.7
orvassantie	90.7	94.8	-4.1
otfectief	97.6	97.6	-0.1
otwate	97.3	97.9	-0.7
overgeels	91	91.4	-0.4
ozoef	96	96.9	-0.9
pabroos	94.4	92.3	2.1
padot	90.3	91.4	-1.1
paknodraal	94.5	95.1	-0.6
pakpaustine	92.2	94.7	-2.6
pamiefel	96	96.5	-0.6
pappief	96	97.2	-1.2
patabeleren	91	92.1	-1.1
pating	90.3	90.2	0.1
pattedrag	94.7	97.1	-2.4
pechtizing	97.3	97.7	-0.4
pegaadade	97.2	97.7	-0.5
pelm	91.2	92.4	-1.2
pespenbaren	97.3	97.2	0.1
pewarante	93.2	97.4	-4.3
pijtelen	92.8	90.1	2.7
pimestarie	91.5	93.9	-2.4

pindermaad	93.1	96.1	-3.1
pinikent	95.7	95.5	0.2
pizeur	94.3	91.7	2.6
pizing	97	97	0
plambing	97.5	97.4	0
plapisitie	90.5	93.5	-2.9
pleuts	92.5	93	-0.5
plurarinaan	95.9	93.3	2.6
podenschap	96	95.4	0.6
pogendaart	95.3	96.4	-1
pomite	93.7	94.4	-0.7
ponenlijk	94.4	95.8	-1.4
posstaan	95	96.6	-1.6
precuguur	90.6	92.1	-1.5
prerartief	92.5	91	1.5
preriment	92.4	91.1	1.2
presiel	90.4	93.3	-2.8
prijnenceld	97.4	97	0.4
prijper	96.6	95.5	1.1
primpel	92.2	91	1.1
priomme	93.2	93.8	-0.6
prip	94.5	93.5	1
prokomoren	92.2	95.9	-3.7
pronippe	93.9	97.4	-3.5
pronvettist	94.3	92.4	1.9
prootsing	93.9	93.5	0.3
prurt	94.5	96	-1.5
puintenij	94.7	92.6	2
radalver	94.3	95.1	-0.8
ralding	91.3	90.2	1.1
ramaxed	97.9	97.7	0.2
rangrictie	93	92.8	0.2
raterliet	93.3	95.9	-2.6
ratmelaar	96.1	96	0
recronaren	93	92.7	0.3
refronleren	91.5	90.4	1.2
relabijn	90.1	91	-0.9
remiketie	93.9	95.1	-1.2
renodregen	93.4	97.6	-4.2
rerdaturaat	92.4	95.2	-2.8
rerk	95.6	97.5	-1.9
resenloren	94	96	-2
reugen	90	90.8	-0.9
rijterzaals	96.1	96.6	-0.5
risanente	90.8	94.8	-4.1
rokeking	97.1	97.3	-0.2
rolebram	95.7	97.3	-1.6
ronbioneel	95.2	95.1	0.1
ronnotutie	90.2	96.2	-6
roportiek	93.2	93.5	-0.4
rortrormig	97.9	97.6	0.4
rumrokking	92.1	97	-4.9

safetantie	92.3	93.7	-1.4
sanoei	97.4	95.1	2.3
sastiem	91.4	91.8	-0.4
schevem	96.5	97.8	-1.3
schiettikt	94.3	91.9	2.4
schijgswerf	95.8	95.7	0
schos	90.9	91.8	-0.9
schroerverei	91.8	94.3	-2.5
schroetster	90.4	91.9	-1.5
schubes	90.3	92.3	-2.1
schurding	92	95.3	-3.3
sefamziet	95.3	94.7	0.6
sekker	92.6	97.5	-4.9
sekortiek	90.5	94.1	-3.6
senigen	95.8	96	-0.2
siltoberen	92.5	93.5	-0.9
simperechtend	93.4	94.4	-1
slaam	92.7	93	-0.3
slabiaan	91.7	95.1	-3.4
slatetie	94	95.6	-1.6
slimmelen	92.8	92.4	0.4
smelieus	94.7	95.8	-1.1
smemalig	94.5	96	-1.5
smeumeren	93.3	90.9	2.5
smeven	93.1	95.1	-2
sneem	95.7	95.6	0.2
snengen	91.3	91.7	-0.4
snink	91.7	94.4	-2.8
snom	97.3	96.1	1.2
snulten	93.2	95.2	-2
soddet	92	93.7	-1.7
sopimetief	93.1	90.4	2.7
sorkeel	93.5	97	-3.5
soskerij	92.8	96.2	-3.4
spendetig	96	97.4	-1.4
spikiek	96.2	96.4	-0.2
spucot	96.2	96.9	-0.7
starm	94.5	95.2	-0.7
statienast	91.1	95.4	-4.3
statsen	93.7	92	1.7
steik	91.6	94.2	-2.7
stezen	90.9	94.2	-3.3
stiefloen	92.8	97.2	-4.4
stobsato	96.6	97	-0.4
storf	91.4	90.2	1.2
stougelin	94	96.4	-2.4
strarokie	96.8	95.9	0.9
stritzent	97.1	97.4	-0.3
struifnever	91	91.9	-0.9
stumolen	90	91.3	-1.3
sturarte	93.9	96.4	-2.5
sturden	93.9	93.1	0.8

suppijkt	95.3	97.1	-1.7
surisie	92.1	92.2	-0.2
sutting	94.4	94.5	-0.1
taben	91.9	94.9	-3
tandeteel	90.8	94.3	-3.5
taukelle	97.6	96.9	0.7
tederie	93.4	92.8	0.7
tegerpijds	97.4	97.4	0
tegissant	92	93.3	-1.3
tekenitie	94.3	95.7	-1.4
teleniaan	90.1	92.7	-2.6
tendetorgie	90.2	94.8	-4.5
teper	91.7	92.4	-0.6
terasonaren	92.6	92	0.6
tergendioneel	94.4	97	-2.6
terutator	90.4	90.8	-0.4
tesignegen	97.2	97.7	-0.5
tetaltive	97.1	97.9	-0.8
tezoper	96.1	97.3	-1.1
tigdering	96.3	97.7	-1.3
tistorie	92.2	93.1	-0.9
tivartie	93.5	93.3	0.2
toedekaat	94	97.2	-3.1
toegons	94.4	96.2	-1.7
toevaping	97.6	97.5	0.1
torlate	97.5	96.3	1.3
tozinees	95.2	97	-1.8
tradidee	97.5	92.8	4.7
traspontij	96.7	95.8	0.9
trimalek	95.5	94.9	0.6
troekens	93.2	96	-2.9
tromsiets	95.6	97.6	-1.9
tront	90.9	92.6	-1.7
twaads	94	95.9	-1.9
tyspude	96.6	97	-0.4
uigenoord	93.5	93	0.6
uitgempen	90	93.5	-3.5
uithoktend	91.6	91.6	0
uitmepoend	97.8	97.8	0
uitsechost	97.2	97	0.2
umpstig	94.2	97.3	-3.1
vadenwinselijk	97.1	96.6	0.5
valetig	92.3	95.4	-3.1
varterel	96.4	96	0.4
vebruchten	93	93.3	-0.3
veding	94	92.4	1.6
vedraand	95.6	96.4	-0.8
veduksfurmer	97.4	97.6	-0.2
vefrarkeerd	96.4	95.1	1.3
vegorgd	96.4	96.7	-0.2
vehegen	93.5	93.2	0.3
vehoenlijk	91.9	94.7	-2.9

vejarist	92	94.8	-2.8
vekedia	97.1	97.6	-0.5
velaalkelijk	96	97.7	-1.7
veleniteerd	95.4	96.2	-0.8
vemouw	97.3	97.8	-0.5
venedegen	93.1	95.7	-2.6
venijnten	92.6	94.4	-1.8
venunding	96.9	97.3	-0.4
vepekenen	93.5	96.9	-3.4
vepikkig	97.4	97.2	0.1
verdasting	90.6	91.2	-0.6
veriede	91.8	92	-0.1
verieen	96.5	94.9	1.6
vernoldaren	97.5	96.7	0.8
verpijding	95	94.6	0.3
verponpenen	95.3	96.4	-1.1
verscheeg	92.6	92	0.6
versellen	91.8	93.4	-1.6
versuppen	94.3	97.6	-3.3
vertijstheid	91.8	94	-2.2
verzieving	92.6	94.7	-2.1
vesolcineerd	92.3	93.3	-1
vespoetening	93.8	96	-2.2
vestesseling	97.1	95.3	1.7
vestraaiing	93	91.6	1.4
vewilleloos	97.9	97.3	0.6
vewiren	97.1	97.6	-0.4
vikoor	92	95.9	-3.9
vildaren	93.3	92.6	0.7
vioron	95.4	94	1.4
vlallen	96.5	97.1	-0.6
vlox	95.2	92.4	2.8
vonlastisch	91.9	94.3	-2.4
vonoom	91.3	93.5	-2.3
vonve	97.5	97.1	0.4
voorkleller	96.6	97.3	-0.6
vrietist	92.9	97	-4.2
vuiding	97.2	95.4	1.8
vukpel	97.5	97.1	0.3
wadiwer	97	97.1	-0.1
waflex	95.6	94.7	0.9
wagerderisch	96.9	97.9	-0.9
wahaliter	94.4	95	-0.6
walarminant	95.5	96.4	-0.9
wamegreren	97.8	97.4	0.4
wanmecutie	93.8	95.6	-1.8
wanomeren	91.8	94	-2.2
warderlijk	92.7	95.5	-2.8
warenator	96.7	96.4	0.3
warspelatie	95.6	95.3	0.3
waspisitie	92.6	94.4	-1.9
wedigrafisch	92.2	93	-0.9

weenschoppelijk	94.5	97	-2.4
welprolen	94.8	97.6	-2.8
wemiak	95.4	97.5	-2.2
wenalia	93.2	97.8	-4.6
wenuratie	95.5	95	0.5
wetterijn	91.2	90.7	0.5
wijranig	95.4	96.7	-1.4
wilheur	91.7	95.7	-4
wilmgroei	92.7	93.7	-1
wistiek	92.7	95.4	-2.7
wrijmegen	96	97.8	-1.8
wuldauto	97.4	97.4	0
zeben	96.8	95.2	1.7
zem	93	95.5	-2.5
zergulatie	92	94.2	-2.2
zervens	97.9	97	0.9
ziekelen	91.8	91.8	-0.1
zieuwerheid	96	95	1
zieuwsbroef	97.1	97.2	-0.1
zimouver	97.8	97	0.8
zismiak	97.7	97.2	0.4
zondalsbond	96.8	96	0.8
zonserheid	96.2	97	-0.8
zoorn	93	95.4	-2.5
zopel	95.8	95.5	0.3
zorditaseren	97.3	97.4	-0.1
zotenilisme	91.5	93.1	-1.6
zujucal	97.5	97.8	-0.3
zummer	96.4	96.3	0.1
zwaldel	92.9	96	-3.1
zwemalaar	93.4	93	0.4
zwidde	94.5	91.4	3.1
zwockbar	97.2	97.3	-0.1
zwoost	94.6	95.5	-0.8

Run 2: Words

Band	Word	Prevalence	Log Google Frequency	Difference Belgium/ Netherlands
1	schuilnaam	1.00	3.49	0.0
1	bijbel	1.00	4.32	0.0
1	kleding	1.00	4.38	0.0
1	schommelen	1.00	3.38	0.0
1	aankondigen	1.00	3.39	0.0
1	tenzij	1.00	4.34	0.0
1	krimpen	1.00	3.71	0.0
1	papier	1.00	4.73	0.0
1	speciaal	1.00	4.58	0.0
1	loodgieter	1.00	3.33	0.0
1	opscheppen	1.00	3.33	0.0
1	uitdelen	1.00	3.47	0.0

1	oppervlakte	1.00	4.21	0.0
1	droomwereld	1.00	3.41	0.0
1	bar	1.00	4.33	0.0
1	pensioen	1.00	4.09	0.0
1	verbluffend	1.00	3.54	0.0
1	maximaal	1.00	4.17	0.0
1	bijgeloof	1.00	3.65	0.0
1	matroos	1.00	3.72	0.0
2	stoer	1.00	3.67	0.0
2	bouwgrond	1.00	3.07	0.0
2	notitieblok	1.00	3.39	0.0
2	arm	1.00	4.74	0.0
2	onschadelijk	1.00	3.47	0.0
2	contact	1.00	5.01	0.0
2	knecht	1.00	3.97	0.0
2	jongleren	1.00	3.34	0.0
2	woordenschat	1.00	3.65	0.0
2	opwarmen	1.00	3.19	0.0
2	postzegel	1.00	3.53	0.0
2	pompoen	1.00	3.36	0.0
2	ingang	1.00	4.43	0.0
2	leerzaam	1.00	3.51	0.0
2	beschaamd	1.00	3.73	0.0
2	tegenstander	1.00	4.02	0.0
2	groeien	1.00	4.36	0.0
2	fietspomp	1.00	2.73	0.0
2	tijdrovend	1.00	3.38	0.0
2	feestelijk	1.00	3.75	0.0
3	verzorgd	1.00	4.10	0.0
3	trekken	1.00	4.85	0.0
3	vraagteken	1.00	3.47	-0.4
3	lerares	1.00	3.65	-0.4
3	handbal	1.00	3.14	0.3
3	kostbaar	1.00	3.82	-0.4
3	bladzijde	1.00	4.05	-0.4
3	onbepaald	1.00	3.60	-0.5
3	bouwjaar	1.00	3.31	-0.4
3	strak	1.00	4.29	-0.4
3	jezelf	1.00	4.43	-0.4
3	paranormaal	1.00	3.25	-0.1
3	ongunstig	1.00	3.63	0.0
3	omzet	1.00	4.02	0.0
3	tegenvaller	1.00	3.37	-0.1
3	typisch	1.00	4.34	-0.4
3	plechtigheid	1.00	3.68	-0.6
3	automatisch	1.00	4.22	-0.4
3	dak	1.00	4.41	-0.4
3	gehoorzaam	1.00	3.60	-0.4
4	opbrengst	1.00	4.12	-0.1
4	ongeldig	1.00	3.26	0.3
4	inspecteren	1.00	3.47	-0.4
4	aanvraag	1.00	4.01	-0.4
4	lopen	1.00	4.99	-0.4

4	speerwerpen	1.00	2.42	0.0
4	opdracht	1.00	4.85	0.3
4	paars	1.00	3.47	0.3
4	aankijken	1.00	3.76	-0.4
4	plakboek	1.00	3.38	-0.1
4	krachtsport	1.00	3.31	-0.1
4	opgave	1.00	4.23	-0.4
4	gezinslid	1.00	3.29	0.3
4	studio	1.00	4.22	0.0
4	opzet	1.00	4.44	-0.4
4	vruchtensap	1.00	3.17	0.0
4	nauwlettend	1.00	3.60	-0.5
4	redden	1.00	4.34	0.3
4	verwennen	1.00	3.47	-0.1
4	hoofdkussen	1.00	3.38	0.0
5	versleten	1.00	3.98	-0.5
5	vriendin	1.00	4.46	-0.1
5	humor	1.00	4.14	-0.4
5	geniaal	1.00	3.50	0.3
5	machinist	1.00	3.50	-0.1
5	bubbelbad	1.00	3.07	-0.1
5	overhalen	1.00	3.66	-0.4
5	controleren	1.00	4.12	-0.1
5	hopen	1.00	4.27	0.3
5	stout	1.00	3.71	-0.5
5	wenskaart	1.00	3.46	-0.4
5	tijdperk	1.00	4.18	0.3
5	opslagplaats	1.00	3.29	-0.4
5	kunst	1.00	5.06	-0.5
5	woest	1.00	3.85	-0.1
5	behandeling	1.00	4.64	-0.1
5	zuiver	1.00	4.24	0.0
5	uitgestrekt	1.00	3.71	-0.5
5	verjaardag	1.00	4.30	-0.1
5	koppeling	1.00	3.76	-0.1
6	herberg	1.00	3.91	-0.1
6	veldslag	1.00	3.63	-0.1
6	gratis	1.00	4.35	-0.1
6	schreeuwen	1.00	4.07	-0.1
6	aanleiding	1.00	4.81	0.0
6	verdediger	1.00	3.61	-0.4
6	rennen	1.00	4.09	-0.6
6	tijdens	1.00	5.25	-0.1
6	zakenvrouw	1.00	3.37	0.0
6	sterk	1.00	5.09	-0.4
6	spinazie	1.00	3.32	-0.1
6	schieten	1.00	4.29	0.3
6	onleesbaar	1.00	3.69	-0.4
6	dichter	1.00	4.81	0.3
6	smerig	1.00	3.65	-0.5
6	docent	1.00	4.16	-0.4
6	toepassing	1.00	4.66	-0.8
6	onafhankelijk	1.00	4.25	-0.1

6	smakelijk	1.00	3.55	0.3
6	chef	1.00	4.33	0.4
7	gevolg	1.00	4.96	-0.1
7	videoband	1.00	2.94	-0.4
7	priester	1.00	4.26	-0.6
7	boomstam	1.00	3.53	-0.2
7	zolder	1.00	3.98	0.6
7	besparing	1.00	3.37	-0.1
7	verpleegster	1.00	3.78	-0.1
7	internationaal	1.00	4.60	0.2
7	waardebon	1.00	2.83	-0.6
7	aarzelend	1.00	3.95	-0.2
7	waaien	1.00	3.70	0.7
7	geweldig	1.00	4.29	0.2
7	deelnemer	1.00	3.65	0.0
7	leider	1.00	4.36	0.3
7	afdak	1.00	3.23	0.2
7	roekeloos	1.00	3.63	0.2
7	textiel	1.00	4.01	-0.1
7	aangenaam	1.00	4.11	-0.1
7	aanmelding	1.00	3.52	0.3
7	scheur	1.00	3.57	0.2
8	bescherming	1.00	4.51	0.6
8	boterham	1.00	3.72	0.3
8	mantel	1.00	4.12	0.6
8	oogarts	1.00	3.32	0.6
8	gevaarlijk	1.00	4.28	-0.1
8	voorschrift	1.00	3.64	0.6
8	winter	1.00	4.67	-0.4
8	concentreren	1.00	4.06	0.3
8	jager	1.00	4.06	-0.2
8	huurhuis	1.00	3.17	-0.6
8	versnelling	1.00	3.68	0.3
8	weergeven	1.00	3.78	0.7
8	nier	1.00	3.62	-0.6
8	negentig	1.00	4.36	-0.3
8	tak	1.00	4.23	-0.2
8	neerslag	1.00	3.84	-0.1
8	monument	1.00	4.21	-0.5
8	beginner	1.00	2.98	-0.3
8	boodschappenlijst	1.00	3.04	-0.1
8	trots	1.00	4.52	0.2
9	beproeving	1.00	3.73	-0.2
9	zichzelf	1.00	5.18	-0.6
9	omlopen	1.00	2.76	-0.1
9	brandweer	1.00	3.65	0.6
9	reservering	1.00	3.23	-0.1
9	onbewoond	1.00	3.56	0.3
9	middelmatig	1.00	3.43	-0.6
9	geslaagd	1.00	4.29	-0.4
9	uitzetten	1.00	3.50	-0.6
9	dronkenschap	1.00	3.59	-0.1
9	trouwens	1.00	4.71	0.3

9	rekenen	1.00	4.44	0.2
9	evenement	1.00	3.68	-0.2
9	begroting	1.00	3.99	-0.5
9	kast	1.00	4.29	-0.5
9	score	1.00	3.84	0.2
9	lente	1.00	4.18	-0.1
9	technologie	1.00	4.23	-0.2
9	taalvaardig	1.00	3.00	0.3
9	genoeg	1.00	5.14	0.3
10	tweevoud	1.00	3.37	-0.6
10	ramp	1.00	4.10	-0.2
10	uitroeien	1.00	3.39	0.6
10	buikdansen	1.00	2.66	0.7
10	geneeskundig	1.00	3.48	0.3
10	gewoonlijk	1.00	4.58	-0.3
10	dempen	1.00	3.39	0.2
10	suikerspin	1.00	3.57	0.7
10	constructie	1.00	4.30	-0.2
10	gunstig	1.00	4.21	0.2
10	omkopen	1.00	3.19	0.2
10	kooktijd	1.00	2.86	-0.2
10	aanbidden	1.00	3.41	0.6
10	wielrenner	1.00	3.28	-0.6
10	lint	1.00	3.80	0.2
10	code	1.00	4.47	-0.1
10	tuintafel	1.00	2.80	-0.1
10	voorzet	1.00	3.29	-0.1
10	danseres	1.00	3.57	-0.1
10	toerisme	1.00	3.86	0.2
11	vakkundig	1.00	3.55	-0.3
11	landmijn	1.00	2.82	0.6
11	tussenstand	1.00	3.25	0.1
11	gezagvoerder	1.00	3.38	0.3
11	stijgend	1.00	3.38	-0.4
11	dobbelsteen	1.00	3.18	-0.2
11	alarmbel	1.00	3.04	-0.3
11	ademhalen	1.00	3.71	0.3
11	zeebodem	1.00	3.41	-0.3
11	brandnetel	1.00	3.17	0.7
11	kin	1.00	4.19	0.6
11	misselijk	1.00	3.82	-0.6
11	pudding	1.00	3.38	0.7
11	roofdier	1.00	3.64	0.7
11	bestuurbaar	1.00	3.00	-0.2
11	verleiden	1.00	3.88	0.3
11	personeel	1.00	4.59	-0.1
11	maatschappelijk	1.00	4.50	0.2
11	vriendschappelijk	1.00	3.49	-0.3
11	stabiliteit	1.00	3.88	-0.1
12	bemachtigen	1.00	3.68	0.7
12	plunderen	1.00	3.43	0.6
12	eeuwig	1.00	4.21	0.2
12	alcohol	1.00	4.02	0.2

12	zondag	1.00	4.43	0.7
12	wachten	1.00	4.92	0.7
12	inhuren	1.00	3.23	0.5
12	zeereis	1.00	3.57	0.1
12	verwerven	1.00	4.21	-0.6
12	prachtig	1.00	4.47	0.7
12	aanduiding	1.00	4.05	0.4
12	ladder	1.00	3.83	0.6
12	netvlies	1.00	3.48	0.5
12	bijverdienen	1.00	3.08	0.2
12	schrappen	1.00	3.67	0.2
12	ringvinger	1.00	3.09	0.6
12	theater	1.00	4.37	0.7
12	logisch	1.00	4.19	-0.1
12	buikspier	1.00	2.32	-0.6
12	eikenhout	1.00	3.35	0.3
13	keuken	1.00	4.64	0.6
13	koolhydraat	1.00	2.85	-0.4
13	overtuigd	1.00	4.53	0.5
13	eetgedrag	1.00	3.12	0.2
13	warmlopen	1.00	2.64	-0.8
13	doelwit	1.00	3.81	0.2
13	bevestigen	1.00	4.06	-0.3
13	flexibiliteit	1.00	3.82	0.0
13	denkwijze	1.00	3.41	0.5
13	idealist	1.00	3.45	0.6
13	soldaat	1.00	4.20	0.6
13	ingevoegd	1.00	3.49	-0.2
13	charmeur	1.00	3.56	0.5
13	morgenavond	1.00	3.35	-0.1
13	achteraan	1.00	3.83	0.6
13	optimisme	1.00	3.80	-0.2
13	balans	1.00	4.62	0.3
13	karton	1.00	3.87	-0.1
13	peer	1.00	3.70	0.5
13	kamerdeur	1.00	3.29	0.5
14	gemeenschap	1.00	4.66	0.8
14	interactief	1.00	3.51	0.2
14	bijhouden	1.00	3.64	0.5
14	verwisselen	1.00	3.37	-0.1
14	reageren	0.99	4.26	1.0
14	kookpot	0.99	3.18	0.7
14	overdonderen	0.99	3.03	0.0
14	keelholte	0.99	2.56	0.6
14	figureren	0.99	3.50	0.0
14	openbaren	0.99	3.62	0.5
14	meetbaar	0.99	3.31	-0.2
14	twintig	0.99	4.83	0.3
14	landing	0.99	3.65	0.5
14	dadelijk	0.99	4.08	0.6
14	aanmaak	0.99	3.20	0.6
14	overgaan	0.99	3.96	-0.8
14	gericht	0.99	4.84	-0.2

14	vastzitten	0.99	3.39	0.5
14	camera	0.99	4.21	-0.1
14	wolf	0.99	4.21	0.2
15	voorwaarde	0.99	4.34	-0.2
15	wasmiddel	0.99	3.17	0.5
15	dobberen	0.99	3.31	-0.6
15	glinsteren	0.99	3.37	-0.1
15	frustreren	0.99	3.16	-0.4
15	minpunt	0.99	3.23	0.0
15	bespreekbaar	0.99	3.48	0.7
15	onaantrekkelijk	0.99	3.41	0.6
15	doek	0.99	4.39	0.7
15	onderhoudend	0.99	4.30	-0.1
15	razend	0.99	3.86	0.3
15	historie	0.99	4.46	0.6
15	delicatesse	0.99	3.23	-0.4
15	begrafenis	0.99	4.11	0.2
15	welvarend	0.99	3.44	-0.6
15	vechtpartij	0.99	3.50	0.6
15	anorexia	0.99	3.36	1.0
15	excellentie	0.99	3.64	-0.5
15	verrassing	0.99	4.17	-0.3
15	linkerkant	0.99	3.58	0.6
16	laklaag	0.99	2.54	-0.4
16	snijdend	0.99	3.31	0.0
16	videocamera	0.99	3.20	-0.3
16	lat	0.99	3.98	0.1
16	routine	0.99	3.71	0.4
16	crematie	0.99	3.43	0.0
16	sponsor	0.99	3.33	-0.8
16	treiteren	0.99	3.39	0.2
16	verbaasd	0.99	4.40	0.3
16	oorsmeer	0.99	2.77	0.5
16	spion	0.99	3.70	1.0
16	kerkstoel	0.99	2.23	0.5
16	schouw	0.99	3.66	0.2
16	leestempo	0.99	3.20	0.0
16	minder	0.99	5.21	0.6
16	zeemansgraf	0.99	3.45	0.0
16	pashok	0.99	2.11	0.7
16	admiraal	0.99	3.97	-0.2
16	percentage	0.99	4.32	0.5
16	doorstrepen	0.99	2.83	-0.6
17	eigenbelang	0.99	3.69	0.0
17	hok	0.99	3.75	0.8
17	rotten	0.99	3.49	0.1
17	nijdig	0.99	3.65	0.4
17	asiel	0.99	3.58	0.4
17	kabeljauw	0.99	3.23	0.6
17	tijger	0.99	3.73	0.9
17	grot	0.99	3.84	0.5
17	versterking	0.99	4.07	0.8
17	bijstellen	0.99	3.34	0.2

17	gestreept	0.99	3.40	0.5
17	detecteren	0.99	3.13	0.0
17	wapperen	0.99	3.48	-0.4
17	optrommelen	0.99	2.66	-0.8
17	voogdij	0.99	3.60	-1.1
17	slok	0.99	3.96	0.5
17	refrein	0.99	3.58	0.0
17	supporter	0.99	3.33	0.5
17	markering	0.99	3.30	0.0
17	stedeling	0.99	3.32	-0.6
18	sjaal	0.99	3.73	0.2
18	ontstaan	0.99	4.94	0.4
18	makkelijk	0.99	4.41	-0.7
18	moleculair	0.99	3.27	-0.7
18	meloen	0.99	3.24	1.1
18	verschijning	0.99	4.18	-0.1
18	regime	0.99	4.29	-0.2
18	koolraap	0.99	2.84	-0.2
18	slenteren	0.99	3.50	0.4
18	vaststelling	0.99	4.10	0.8
18	onthalen	0.99	2.98	-0.6
18	volleybal	0.99	3.13	-0.7
18	bewerkstelligen	0.99	3.86	-0.5
18	ontzag	0.99	3.81	0.1
18	herhaaldelijk	0.99	4.17	-0.1
18	ijsvrij	0.99	3.16	-0.7
18	afstempelen	0.99	2.53	0.2
18	belagen	0.99	3.17	-0.7
18	zuidwaarts	0.99	3.45	-0.8
18	ondeelbaar	0.99	3.35	0.0
19	menslievend	0.99	2.72	-0.5
19	muf	0.99	3.32	-0.1
19	mailen	0.99	3.48	0.9
19	principieel	0.99	3.84	0.2
19	omploegen	0.99	2.66	-0.3
19	opvliegend	0.99	3.29	1.3
19	stopzetten	0.99	3.36	1.2
19	fascineren	0.99	3.45	0.2
19	misvormd	0.99	3.32	0.6
19	doortocht	0.99	3.52	0.8
19	microchip	0.99	2.83	-0.5
19	analfabetisme	0.99	3.26	-1.0
19	statistisch	0.99	4.26	-0.6
19	levenslustig	0.99	3.40	-0.1
19	alvleesklier	0.99	2.98	-1.4
19	nomineren	0.99	3.32	-0.7
19	verschroeien	0.99	2.74	0.0
19	trol	0.99	3.52	0.1
19	borstkas	0.99	3.62	0.8
19	vertering	0.99	3.01	1.0
20	aanstormen	0.99	2.73	-1.0
20	flauwvallen	0.99	3.17	0.3
20	sabotage	0.99	3.61	0.6

20	naaldbos	0.99	2.72	0.5
20	weliswaar	0.99	4.74	-0.5
20	krantenkop	0.99	3.01	-0.6
20	wapentuig	0.99	3.27	0.0
20	eliminatie	0.99	3.19	0.4
20	korset	0.99	3.21	1.2
20	implantaat	0.99	2.79	-0.2
20	lafaard	0.99	3.54	-1.1
20	opborrelen	0.99	3.24	-1.1
20	zeedijk	0.99	3.44	-0.9
20	stoomwolk	0.99	2.06	-1.7
20	karakteriseren	0.99	3.52	1.1
20	wijnoogst	0.99	2.75	-1.4
20	begeerd	0.99	3.58	-0.7
20	uitbrengen	0.99	3.84	1.7
20	lichtschakelaar	0.99	2.61	0.7
20	stempeldoos	0.99	1.85	0.4
21	autisme	0.99	3.29	0.1
21	reiniger	0.99	2.69	0.4
21	getintel	0.99	1.93	0.8
21	administratief	0.99	3.96	1.5
21	abstractie	0.99	3.60	0.4
21	rijverbod	0.99	2.42	1.8
21	vaartijd	0.99	2.26	0.9
21	driedelig	0.99	3.52	-0.5
21	gedisciplineerd	0.99	3.34	1.6
21	binnenstebuiten	0.99	3.56	-1.7
21	recensie	0.99	4.02	0.1
21	reactor	0.99	3.38	1.0
21	terugkrijgen	0.99	3.39	0.3
21	aantrekking	0.99	3.24	-0.1
21	poppenwagen	0.99	2.85	1.0
21	vlekkeloos	0.99	3.48	1.1
21	koudbloedig	0.99	2.55	-0.5
21	filiaal	0.99	3.58	0.1
21	zeevogel	0.99	2.61	0.5
21	gesmeek	0.99	2.07	-0.4
22	daarheen	0.99	3.73	0.2
22	hagelen	0.99	2.72	-1.4
22	prinses	0.99	4.30	1.1
22	westelijk	0.99	3.85	1.3
22	presidentieel	0.99	3.08	1.7
22	debatteren	0.99	3.37	-1.0
22	alleseter	0.99	3.42	-0.6
22	afzweren	0.99	3.09	0.3
22	eentalig	0.99	2.88	0.2
22	hoofdtaal	0.99	2.32	-0.8
22	dessert	0.99	3.46	2.0
22	pathetisch	0.99	3.49	-1.0
22	bestijgen	0.99	3.33	-0.8
22	weglokken	0.99	2.25	0.9
22	kippenvlees	0.99	2.70	1.2
22	schoolbal	0.99	2.85	1.3

22	verbouwer	0.99	2.23	0.4
22	handenarbeid	0.99	3.36	-0.7
22	opgeleide	0.99	3.67	0.4
22	abonnement	0.99	3.93	0.4
23	aluminiumfolie	0.98	3.31	0.8
23	vastlijmen	0.98	2.31	0.1
23	lawaaierig	0.98	3.03	1.2
23	staand	0.98	3.76	0.0
23	opfokken	0.98	2.66	0.1
23	voorafgaan	0.98	3.41	0.0
23	schimmig	0.98	3.40	-1.8
23	butler	0.98	3.81	0.4
23	epicentrum	0.98	3.48	0.0
23	orthodoxe	0.98	3.84	-1.5
23	teugel	0.98	3.35	-0.7
23	rouwdag	0.98	1.97	1.1
23	onvoldaan	0.98	2.96	0.7
23	openhouden	0.98	3.27	1.4
23	tennisser	0.98	3.18	-0.9
23	confetti	0.98	3.42	1.0
23	marsepein	0.98	3.50	0.2
23	geknik	0.98	2.53	-1.7
23	biljet	0.98	3.43	1.1
23	onderbewust	0.98	2.87	0.0
24	zwartmaken	0.98	2.53	-0.3
24	afzienbaar	0.98	0.95	0.4
24	ontgoochelen	0.98	2.58	1.4
24	uitlokking	0.98	2.71	0.8
24	hyena	0.98	3.42	-1.5
24	verdrukken	0.98	2.51	0.7
24	wrakstuk	0.98	2.15	-0.6
24	wegdenken	0.98	2.92	-1.0
24	doodgaan	0.98	3.74	0.2
24	afwerpen	0.98	3.28	-2.2
24	monopolie	0.98	3.71	-0.4
24	gewichtheffer	0.98	2.87	0.0
24	bevliegen	0.98	1.65	-1.1
24	stroperij	0.98	2.63	-2.0
24	cardiogram	0.98	2.25	-2.1
24	ongenoemd	0.98	3.28	-0.9
24	zoekwerk	0.98	3.16	-0.7
24	daartussen	0.98	3.75	1.5
24	opwellen	0.98	3.20	-1.9
24	toedragen	0.98	3.23	-1.4
25	geflits	0.98	2.51	-2.2
25	schel	0.98	3.54	-0.6
25	finaal	0.98	3.42	1.4
25	taalschrift	0.98	2.84	-0.8
25	ongeneeslijk	0.98	3.55	0.2
25	gespartel	0.98	2.50	1.3
25	armgebaar	0.98	2.72	-1.0
25	toewuiven	0.98	2.09	0.3
25	verleren	0.98	2.99	-1.0

25	onbebouwd	0.98	2.87	0.5
25	gejodel	0.98	2.05	-0.2
25	optelling	0.98	2.97	2.1
25	whisky	0.98	3.29	-1.4
25	bibliothecaris	0.98	3.70	-0.9
25	ondertand	0.98	1.00	-1.7
25	prothese	0.98	3.19	0.6
25	bestelen	0.98	3.15	-0.1
25	imitatie	0.98	3.63	1.4
25	omdoen	0.98	2.99	0.1
25	babysitten	0.98	3.15	1.6
26	jazzdans	0.98	2.44	-1.1
26	nabloei	0.98	3.15	-0.1
26	pizzeria	0.98	3.30	2.8
26	kernzin	0.98	3.04	0.9
26	elektrocuteren	0.98	2.35	-2.3
26	smeedbaar	0.98	1.79	-0.7
26	weerkunde	0.98	3.18	1.3
26	parabool	0.98	3.05	1.8
26	trilogie	0.98	3.82	2.2
26	betweter	0.98	3.24	1.2
26	zeilpak	0.98	1.64	-0.9
26	afschuren	0.98	2.06	1.6
26	verkwikken	0.97	2.68	-2.7
26	tofu	0.97	3.14	0.4
26	omkering	0.97	3.60	1.2
26	regulatie	0.97	2.97	-2.0
26	aanmoedigend	0.97	2.98	2.1
26	dienstvaardig	0.97	2.56	-0.1
26	sacrament	0.97	3.72	2.3
26	gebloemd	0.97	2.94	-1.5
27	prutserig	0.97	0.95	-1.6
27	ongevormd	0.97	2.44	-1.2
27	overdekken	0.97	2.68	3.0
27	rijspoor	0.97	0.30	1.0
27	opklappen	0.97	1.74	-2.0
27	rangtelwoord	0.97	2.21	-0.2
27	karamel	0.97	3.24	0.6
27	wijden	0.97	3.94	-0.9
27	optrek	0.97	2.73	1.9
27	trekroute	0.97	2.27	1.3
27	neigen	0.97	3.34	-1.8
27	vredespad	0.97	0.95	-1.9
27	ontzegeld	0.97	2.62	-2.7
27	symmetrie	0.97	3.46	1.2
27	katrol	0.97	2.79	-1.8
27	gelofte	0.97	3.42	-0.6
27	koningschap	0.97	3.65	-2.3
27	ijzeren	0.97	4.26	-0.2
27	skireis	0.97	0.90	1.0
27	wier	0.97	4.27	-1.2
28	toehoren	0.97	2.34	-3.4
28	eikenboom	0.97	3.25	-3.8

28	vechthaan	0.97	3.47	2.0
28	groothartig	0.97	2.28	1.1
28	fietsrit	0.97	2.08	2.4
28	godsleer	0.97	2.86	-2.0
28	alchemie	0.97	3.49	-3.2
28	milligram	0.97	2.76	1.6
28	druppen	0.97	3.03	-3.8
28	homogeniteit	0.97	3.30	0.3
28	burgemeester	0.97	4.49	2.7
28	stookhout	0.97	2.05	-3.7
28	onderrichten	0.97	3.13	2.8
28	huurbasis	0.97	1.89	0.4
28	kreuk	0.96	3.42	0.4
28	afgrijzen	0.96	3.63	-3.4
28	verbluft	0.96	3.57	2.2
28	klimnet	0.96	1.00	0.4
28	copiloot	0.96	2.72	-0.1
28	bovenhalen	0.96	2.93	3.3
29	schouwing	0.96	3.00	0.6
29	wetteloos	0.96	2.93	-3.2
29	omblazen	0.96	1.26	-1.2
29	ruggenwervel	0.96	2.29	-3.1
29	opzicht	0.96	4.52	3.6
29	ereplicht	0.96	2.68	-3.3
29	dijkweg	0.96	2.48	0.1
29	grindpad	0.96	3.01	-1.7
29	incalculeren	0.96	2.49	4.3
29	opdroging	0.96	1.72	-3.4
29	goedgehumeurd	0.96	2.81	-2.9
29	kerkraad	0.96	2.38	3.5
29	rechtszaak	0.96	3.67	-0.3
29	nevenschikking	0.96	3.12	1.2
29	asymmetrisch	0.96	3.13	1.6
29	toonval	0.96	2.27	-2.8
29	nazomers	0.96	1.89	-2.6
29	inpraten	0.96	2.66	2.0
29	observant	0.96	3.36	-3.8
29	hernieuwen	0.96	3.07	2.2
30	vuurhaard	0.96	2.59	-2.0
30	corporatie	0.96	3.18	-1.5
30	astmatisch	0.96	2.59	-1.9
30	afremming	0.96	2.35	1.9
30	immens	0.96	3.61	1.9
30	balseming	0.96	2.54	-2.8
30	rationalisme	0.96	3.45	2.8
30	afdwaling	0.96	2.47	-0.8
30	aangapen	0.95	2.50	-4.2
30	pubertijd	0.95	3.18	1.4
30	sportieveling	0.95	2.49	3.5
30	uitboren	0.95	1.83	1.6
30	heidendom	0.95	3.40	1.5
30	kleinhartig	0.95	0.85	0.8
30	onaannemelijk	0.95	3.18	1.3

30	busstop	0.95	1.95	2.1
30	openlaten	0.95	2.74	-0.2
30	terugkopen	0.95	2.59	0.4
30	apporteren	0.95	3.23	-2.8
30	spiercel	0.95	2.00	2.1
31	notatie	0.95	3.17	-3.0
31	radicaliteit	0.95	3.24	2.1
31	bijslag	0.95	2.20	1.0
31	vlakker	0.95	2.94	-1.1
31	warmhartig	0.95	2.55	3.4
31	aankleven	0.95	2.95	2.6
31	uitvragen	0.95	2.43	4.0
31	piranha	0.95	3.41	-2.7
31	omhulling	0.95	2.92	-1.9
31	dienstig	0.95	3.42	-0.4
31	hartstikke	0.95	3.66	-0.9
31	assenstelsel	0.95	2.56	0.5
31	eromheen	0.95	3.84	0.8
31	onvertakt	0.95	1.94	3.8
31	suspensie	0.95	2.69	3.3
31	gedachtegang	0.95	3.56	-1.6
31	ervandoor	0.95	3.79	2.0
31	ploegarts	0.94	1.90	4.0
31	draaidag	0.94	2.53	4.5
31	evengoed	0.94	3.90	-3.0
32	aggregatie	0.94	2.87	4.0
32	omwerpen	0.94	1.96	0.0
32	punctuur	0.94	1.80	0.5
32	voorlaten	0.94	0.95	2.4
32	bekoeling	0.94	2.20	-3.9
32	persistentie	0.94	2.71	-1.5
32	peilloos	0.94	3.08	-2.3
32	ongeleerd	0.94	2.29	2.8
32	bijzaal	0.94	0.78	-0.6
32	hatelijkheid	0.94	2.67	-1.8
32	hoogstnodig	0.94	2.12	2.2
32	afladen	0.94	2.19	-0.3
32	hardmaken	0.94	1.40	-1.8
32	kangoeroe	0.94	3.59	4.9
32	boeddhist	0.94	3.23	-0.5
32	uitdenken	0.94	3.08	-1.4
32	drukplek	0.94	0.00	-4.5
32	hydrofoob	0.94	2.09	1.4
32	betuttelaar	0.93	0.70	-0.6
32	uurdienst	0.93	1.00	-6.8
33	induiken	0.93	3.08	-1.3
33	opkrullen	0.93	2.58	3.7
33	bebossen	0.93	2.41	3.2
33	inneming	0.93	2.88	-2.7
33	glorievol	0.93	1.96	-0.3
33	detineren	0.93	1.97	-4.2
33	omkantelen	0.93	1.81	6.5
33	hiervandaan	0.93	3.49	0.7

33	meedogend	0.93	2.63	-2.0
33	onbeschrijflijk	0.93	3.33	0.5
33	overlever	0.93	3.65	0.1
33	ongelijnd	0.93	0.85	-4.6
33	neerknielen	0.93	2.61	3.8
33	lijfknecht	0.93	2.32	0.2
33	delinquentie	0.93	2.80	6.1
33	voeteinde	0.93	2.14	7.0
33	diabeticus	0.93	2.39	6.3
33	verlating	0.93	3.43	-1.9
33	korstig	0.93	2.18	0.7
33	afzadelen	0.93	1.85	-3.2
34	heenbrengen	0.92	1.75	-2.4
34	naartoe	0.92	4.31	4.7
34	manoeuvreren	0.92	3.44	2.7
34	empirisme	0.92	3.18	0.8
34	afstormen	0.92	2.60	-4.3
34	voorlicht	0.92	2.46	3.6
34	geleedpotige	0.92	2.16	1.3
34	psychoot	0.92	2.81	3.9
34	gestipt	0.92	2.16	3.5
34	zonnewering	0.92	2.45	6.2
34	rasdier	0.92	0.78	4.2
34	opberging	0.92	2.30	5.4
34	rodekool	0.92	2.76	-3.1
34	sluimerig	0.92	0.78	-1.8
34	tsjirpen	0.92	3.18	3.4
34	knaagtand	0.91	1.00	-0.6
34	ingewand	0.91	2.77	-4.2
34	dwingerig	0.91	1.87	-4.6
34	terminatie	0.91	1.97	1.0
34	inglijden	0.91	1.90	0.6
35	deconstructief	0.91	2.10	4.1
35	generatief	0.91	2.68	3.0
35	grafkapel	0.91	2.93	4.1
35	eroverheen	0.91	3.57	2.1
35	fertilisatie	0.91	2.50	2.2
35	schranzen	0.91	2.02	-1.9
35	fopperij	0.91	2.43	-4.5
35	veeteler	0.91	1.81	6.3
35	decideren	0.91	1.98	3.1
35	duellist	0.91	2.21	-0.7
35	regressief	0.91	2.75	6.0
35	onheilig	0.90	3.33	1.0
35	erkentenis	0.90	2.83	3.5
35	distractie	0.90	2.07	-0.5
35	wilsgebrek	0.90	2.35	0.8
35	objectie	0.90	2.31	4.1
35	watertrappen	0.90	2.91	4.0
35	ophitsing	0.90	2.63	1.0
35	vaardigen	0.90	3.18	-2.3
35	ouderdag	0.90	2.03	4.0
36	incongruentie	0.90	2.75	2.2

36	spijten	0.90	2.87	-1.4
36	affectief	0.90	3.14	1.3
36	berispelijk	0.90	2.14	2.6
36	determinisme	0.90	3.30	2.3
36	doorspekken	0.89	2.85	-3.9
36	treuzelig	0.89	1.00	-5.0
36	uitschateren	0.89	1.76	0.4
36	kwellerij	0.89	0.90	-0.1
36	insoppen	0.89	0.00	4.1
36	wetgeleerde	0.89	2.30	1.5
36	nokvol	0.89	2.39	0.5
36	ongeuit	0.89	0.60	-0.8
36	flansen	0.89	2.73	-1.8
36	gedans	0.89	2.42	-3.1
36	omsnoeren	0.89	1.00	4.1
36	zorgvol	0.89	2.34	3.8
36	afspeuren	0.89	2.72	3.6
36	tinkelen	0.89	2.96	-4.8
36	beleerd	0.88	2.52	0.1
37	kosmoloog	0.88	2.59	-0.7
37	omgrenzen	0.88	2.01	1.2
37	gewaterd	0.88	2.13	-0.6
37	voorhebben	0.88	2.93	3.6
37	bergkap	0.88	0.30	0.2
37	belezing	0.88	0.95	2.7
37	grauwig	0.88	2.00	-3.2
37	tijdmaat	0.88	0.78	1.7
37	divergerend	0.88	2.18	3.7
37	smakker	0.88	0.78	-2.6
37	opraapsel	0.88	0.00	2.2
37	regulair	0.88	1.63	-4.7
37	curatief	0.87	2.85	5.9
37	godzalig	0.87	2.70	-2.1
37	onhelder	0.87	2.93	4.9
37	pakbaar	0.87	0.00	-3.5
37	welgeaard	0.87	0.30	-1.7
37	opticus	0.87	2.21	-0.6
37	executant	0.87	2.53	-3.2
37	affluiten	0.87	0.85	4.0
38	badkuur	0.87	2.18	3.1
38	zelfachting	0.87	2.21	4.0
38	zwaarheid	0.87	2.20	1.7
38	vitalist	0.87	3.63	5.5
38	ertegenover	0.87	3.16	4.2
38	dwarshoofd	0.87	0.00	3.7
38	opproppen	0.86	0.70	-5.7
38	toebuigen	0.86	1.87	2.1
38	lymf	0.86	2.08	3.6
38	besnoeien	0.86	2.42	4.2
38	expliciteren	0.86	3.21	-4.8
38	zinsritme	0.86	2.20	-2.3
38	telkaart	0.86	0.95	0.2
38	rijmer	0.86	3.14	-4.2

38	almaar	0.85	3.85	3.6
38	afvliegen	0.85	2.56	-2.2
38	nablaffen	0.85	0.30	-1.8
38	petrischaal	0.85	1.91	-3.8
38	dunheid	0.85	2.20	3.0
38	zaniker	0.85	1.66	-0.5
39	condoleantie	0.84	1.85	-6.3
39	toehalen	0.84	2.17	3.4
39	zwerfblok	0.84	0.70	-2.9
39	ruiler	0.84	1.76	-1.9
39	stembreuk	0.84	0.95	3.7
39	stoorder	0.84	2.17	2.4
39	klammig	0.84	1.62	-7.3
39	harpenist	0.84	0.70	-4.0
39	bijenboer	0.84	0.00	-2.6
39	hinkelaar	0.84	0.60	1.2
39	druksel	0.83	3.32	0.4
39	bontheid	0.83	2.46	-6.2
39	prevalentie	0.83	3.13	-7.0
39	bespikkelen	0.83	0.70	-4.3
39	koploos	0.83	0.30	-4.5
39	aangehuwd	0.83	1.00	-6.6
39	extraheren	0.83	2.42	3.4
39	wegsterven	0.83	3.07	-3.8
39	drukvel	0.83	1.00	5.1
39	instormen	0.83	1.89	-3.4
40	erfhuis	0.83	2.46	6.7
40	bijliggen	0.83	2.44	1.1
40	groeipad	0.82	2.48	1.0
40	vetput	0.82	0.60	-6.3
40	bijspelen	0.82	0.90	-3.6
40	gevest	0.82	2.90	-4.1
40	huisgod	0.82	2.74	-0.6
40	purperen	0.82	3.71	-0.8
40	predicatief	0.82	2.45	1.7
40	loopdag	0.82	0.48	-2.0
40	maffer	0.82	3.50	-5.4
40	vetbuik	0.82	0.60	1.5
40	mediaal	0.81	2.83	6.1
40	platkop	0.81	1.43	1.3
40	vergrauwd	0.81	2.10	4.6
40	poolshoogte	0.81	3.41	-0.3
40	lamelle	0.81	1.87	-7.5
40	snaterbek	0.81	0.70	0.3
40	delegaat	0.81	2.24	1.8
40	dekstuk	0.81	1.60	-3.0
41	kruisnet	0.80	1.94	2.1
41	afkrabsel	0.80	0.00	6.0
41	vermaking	0.80	2.01	3.1
41	radiatie	0.80	2.19	9.5
41	biogeen	0.80	0.95	-1.1
41	futselen	0.79	1.86	-4.0
41	onsubtiel	0.79	1.88	6.1

41	sopperig	0.79	0.78	-7.0
41	patroneren	0.79	2.13	7.3
41	sperren	0.79	2.63	2.5
41	aanmengen	0.79	0.48	-4.3
41	verzouten	0.79	2.10	3.4
41	endotherm	0.78	0.85	1.0
41	zilverig	0.78	2.95	-1.5
41	katalyse	0.78	2.53	8.5
41	leesgraag	0.78	2.06	-0.6
41	blondheid	0.78	2.13	2.0
41	legalisme	0.78	2.50	-1.5
41	homeostase	0.78	2.70	-2.0
41	trekvis	0.78	0.95	2.8
42	polynoom	0.78	2.24	-6.7
42	eedbreuk	0.78	1.87	2.9
42	eelterig	0.78	0.00	9.1
42	ordinaat	0.77	1.75	3.4
42	enigst	0.77	2.59	-7.3
42	omdoping	0.77	1.88	-1.6
42	omtollen	0.77	0.00	-5.3
42	gloeierig	0.76	0.00	6.6
42	steilte	0.76	2.64	-5.6
42	stamgod	0.76	2.28	-0.8
42	hersmeden	0.76	1.80	7.0
42	bemoeiing	0.76	2.60	3.4
42	pruttelig	0.76	0.00	-1.9
42	oproeien	0.76	2.56	-0.9
42	casseren	0.76	2.27	-5.1
42	animaal	0.76	2.94	7.1
42	heterofilie	0.75	0.00	6.8
42	afdoener	0.75	0.70	-6.5
42	zinking	0.75	1.93	-4.6
42	ontzinken	0.75	2.13	4.6
43	taxonoom	0.75	2.26	4.7
43	slijping	0.75	0.30	-0.4
43	fonoloog	0.74	1.94	4.7
43	libertair	0.74	2.85	4.5
43	annoteren	0.74	2.80	-8.9
43	kruisarm	0.73	1.62	-4.0
43	inbegrip	0.73	3.74	3.1
43	poezig	0.73	2.33	5.9
43	brongas	0.73	0.70	1.5
43	deprivatie	0.73	2.81	3.2
43	drijftol	0.73	0.95	-3.4
43	fenotype	0.73	2.49	5.6
43	bijenstal	0.72	2.62	-5.9
43	ijsschuit	0.72	1.89	-5.8
43	insolide	0.72	0.48	8.3
43	bekruisen	0.72	1.76	4.5
43	universaliteit	0.72	3.31	4.3
43	aversief	0.72	1.79	7.2
43	hartroerend	0.72	2.54	-8.5
43	morzelen	0.71	0.60	2.2

44	overjagen	0.71	0.30	6.9
44	aansmeden	0.71	0.00	7.5
44	siersel	0.71	2.31	4.5
44	wortelig	0.70	0.30	-1.9
44	zegswijs	0.70	1.41	1.0
44	wolgoed	0.70	0.30	-1.0
44	stuifbal	0.70	0.48	-0.7
44	lobberig	0.69	0.00	-5.4
44	demissie	0.69	2.31	5.9
44	peigeren	0.69	1.86	7.6
44	soezig	0.69	1.90	-6.3
44	pedologie	0.69	2.28	-5.3
44	reflexief	0.69	3.13	7.7
44	voorwal	0.68	0.30	-7.1
44	bekappen	0.68	0.95	-0.6
44	totaliter	0.68	2.34	2.6
44	sufferig	0.68	1.85	9.4
44	biggelen	0.68	2.66	-5.4
44	nestvlieder	0.68	1.73	-6.1
44	cellijn	0.68	1.79	-2.7
45	inexact	0.67	2.33	1.4
45	prohibitief	0.67	2.20	4.4
45	afbietsen	0.67	0.00	-3.7
45	beknorren	0.67	1.72	-7.0
45	omdwalen	0.67	0.78	-4.8
45	incidentie	0.67	3.14	4.6
45	cesarisme	0.66	0.90	1.6
45	dompen	0.66	1.92	-1.5
45	knarsing	0.66	0.70	2.2
45	woestaard	0.66	2.22	-8.2
45	pretorium	0.66	1.85	7.6
45	sidderrog	0.65	2.09	7.9
45	pletteren	0.65	1.91	-1.7
45	impenetrabel	0.65	0.48	0.5
45	hagelsnoer	0.65	0.60	1.4
45	raagbol	0.64	1.48	-3.0
45	belijmen	0.64	0.48	0.6
45	tolplan	0.64	0.00	4.6
45	invangen	0.64	1.61	-8.0
45	flinterig	0.64	0.48	-0.4
46	stijgtijd	0.63	0.48	3.8
46	kraanoog	0.62	0.00	4.3
46	bijbank	0.62	2.38	1.0
46	diastolisch	0.62	1.86	4.7
46	stilet	0.62	2.64	-6.2
46	spinaal	0.61	1.94	6.4
46	klankwet	0.61	2.06	3.8
46	arterieel	0.61	2.28	3.6
46	ontstelen	0.61	2.46	5.0
46	juvenaat	0.61	2.45	-1.0
46	presumptie	0.61	2.41	9.6
46	duimeling	0.60	0.78	1.7
46	duplolamp	0.60	0.30	-4.6

46	oncogeen	0.60	0.70	7.8
46	donderaar	0.60	1.80	-2.8
46	situatief	0.60	2.16	-6.8
46	ijseend	0.60	0.85	5.3
46	voltekend	0.59	1.68	4.8
46	verzenuwd	0.59	2.02	0.3
46	strammig	0.59	0.00	-3.9
47	toongat	0.58	0.30	-2.6
47	paganist	0.58	1.92	-3.5
47	puncteren	0.58	1.70	1.3
47	zuigsnuit	0.58	1.62	-6.0
47	watteren	0.58	1.62	-1.2
47	serologie	0.58	2.01	4.0
47	weduwgift	0.57	0.00	6.1
47	afmijnen	0.57	0.90	-9.1
47	anomie	0.57	2.69	3.2
47	keelzak	0.57	2.07	2.0
47	liniatuur	0.56	0.95	1.9
47	equatie	0.56	2.26	8.5
47	ratsen	0.56	1.97	5.4
47	triarchie	0.55	0.70	8.4
47	bindwilg	0.55	1.00	-5.0
47	afzanding	0.55	1.76	0.1
47	lipogram	0.55	2.51	3.8
47	retorsie	0.54	2.08	7.9
47	homonymie	0.54	2.74	7.7
47	betering	0.54	2.73	4.8
48	cyanose	0.53	2.13	-2.8
48	confiseur	0.53	2.49	1.6
48	sarrig	0.53	0.48	-8.8
48	neuronaal	0.52	1.95	9.8
48	patroniem	0.52	2.72	8.9
48	dekhuis	0.52	1.72	2.9
48	ijsappel	0.51	0.30	7.9
48	exemplair	0.50	1.77	4.8
48	keuterij	0.50	0.60	8.6
48	afmijning	0.50	0.48	-9.4
48	walgang	0.50	0.78	-3.7
48	pronomen	0.49	3.07	6.4
48	waaraf	0.49	2.64	7.4
48	desambigueren	0.48	0.85	8.4
48	indologie	0.48	2.82	2.1
48	titreren	0.48	1.64	1.4
48	palatum	0.48	2.21	6.8
48	mesoderm	0.48	1.79	4.5
48	cultisch	0.48	2.30	-0.5
48	roffelig	0.48	0.00	-1.1
49	pakmand	0.47	0.60	1.1
49	maatvis	0.47	0.00	-4.7
49	schoring	0.46	0.70	-4.3
49	ossuarium	0.45	2.41	7.1
49	failleren	0.45	2.02	1.3
49	tijrivier	0.45	0.30	3.5

49	heliosis	0.44	2.68	6.3
49	statieus	0.43	0.85	-3.7
49	chiasma	0.43	2.18	-3.9
49	klipvis	0.43	1.93	-2.1
49	tanktas	0.43	0.00	-7.5
49	persevereren	0.43	0.90	6.3
49	tablatuur	0.42	0.60	11.0
49	macrofaag	0.42	1.60	5.7
49	tekenaap	0.42	0.85	-4.1
49	raseren	0.42	1.62	2.9
49	oxidase	0.42	2.43	6.2
49	bomig	0.42	0.00	-0.5
49	statuesk	0.42	0.60	6.4
49	trachiet	0.41	2.45	-6.1
50	judicium	0.41	2.61	1.3
50	pernicieus	0.41	2.15	4.2
50	decaloog	0.41	2.61	4.4
50	trypsine	0.40	1.71	4.0
50	fibril	0.39	1.43	9.0
50	smedig	0.39	2.23	0.2
50	velijn	0.39	3.07	-4.9
50	heisteren	0.39	0.48	-7.6
50	glosseren	0.38	0.78	5.8
50	slinking	0.38	0.90	-1.8
50	gebroekt	0.38	1.00	-2.0
50	ostracisme	0.37	2.66	8.1
50	galantine	0.37	0.90	-2.1
50	egotisme	0.37	2.53	5.0
50	majoraat	0.37	1.82	5.8
50	rituaal	0.37	2.72	-5.8
50	teratogeen	0.36	1.72	8.5
50	bentoniet	0.36	1.82	-2.8
50	picogram	0.36	0.78	-3.5
50	dalmatiek	0.35	1.98	7.7
51	revideren	0.35	1.81	-8.3
51	axon	0.34	2.55	9.8
51	vasopressine	0.33	2.19	3.6
51	stuwage	0.33	1.81	4.9
51	meerkol	0.33	0.30	6.8
51	titaniet	0.32	0.90	3.7
51	neutrum	0.32	2.59	-1.4
51	repelen	0.32	1.00	-4.0
51	lemuur	0.32	1.62	0.1
51	kordeel	0.31	0.00	8.4
51	illiquide	0.31	1.67	2.7
51	gitten	0.31	2.43	-4.3
51	hortoloog	0.30	2.19	7.0
51	holoniem	0.30	0.00	2.5
51	trigram	0.30	1.99	-3.2
51	talhout	0.30	2.26	-4.2
51	provenu	0.28	1.94	2.9
51	siderisch	0.28	1.91	0.8
51	quaestrix	0.28	0.70	-0.6

51	fomenteren	0.27	0.48	1.9
52	exantheem	0.26	1.88	-0.6
52	cenotaaf	0.26	2.86	9.9
52	spinel	0.25	2.15	0.2
52	vaceren	0.25	2.06	-7.9
52	refactie	0.24	0.48	2.1
52	rekwirant	0.24	0.95	4.9
52	miasma	0.23	2.46	-4.3
52	kloptor	0.23	0.30	-2.2
52	strekel	0.22	0.78	-2.1
52	grafeem	0.22	2.37	-1.9
52	katabool	0.21	1.00	8.1
52	pellagra	0.21	2.06	-2.2
52	solipsist	0.21	3.41	-2.1
52	jangat	0.21	0.48	-0.6
52	prosodie	0.20	3.22	8.4
52	mantiek	0.20	2.57	0.4
52	sinopel	0.20	2.43	3.0
52	sluif	0.20	0.48	5.0
52	dukaton	0.19	2.04	-7.0
52	paplam	0.19	0.60	-2.8
53	harpuis	0.19	1.76	1.4
53	anakoloet	0.18	2.40	-4.6
53	meristeem	0.18	1.63	-2.2
53	heulsap	0.17	1.95	-4.9
53	viaticum	0.16	2.48	2.2
53	labarum	0.16	2.66	-3.0
53	salpicon	0.16	0.90	3.5
53	grietenij	0.16	2.85	-4.5
53	pelagiaan	0.15	0.70	1.7
53	liplap	0.15	2.22	3.4
53	acribie	0.15	2.78	0.1
53	indult	0.14	2.04	-3.7
53	decalogus	0.14	0.85	3.3
53	kwabaal	0.14	2.00	6.3
53	beug	0.14	2.28	5.1
53	silurisch	0.14	0.30	2.9
53	zwade	0.13	0.90	-1.0
53	apocope	0.12	2.67	4.7
53	salangaan	0.12	0.60	0.4
53	lumbecken	0.11	1.88	-0.4
54	deiktisch	0.10	2.08	1.0
54	slatten	0.10	0.48	-0.7
54	smuiger	0.10	0.60	-0.3
54	jarosiet	0.09	0.95	3.2
54	putto	0.09	3.24	1.8
54	entrijs	0.08	0.00	-3.8
54	malvezij	0.07	0.95	-1.0
54	salmi	0.07	2.57	1.6
54	pinyin	0.06	2.64	2.2
54	kebon	0.06	2.76	-1.9
54	seider	0.05	2.39	-0.8
54	loeach	0.05	0.90	0.6

54	romusha	0.04	2.81	0.0
54	sfagnum	0.04	0.70	1.7
54	tussor	0.03	0.60	1.4
54	ghazel	0.03	2.18	0.0
54	dawet	0.03	0.70	0.0
54	ypresien	0.03	0.70	1.6
54	saguweer	0.02	1.60	0.5
54	hoedna	0.02	0.78	0.0

Run 2: Non-words

**Non-word**	**%correct Belgium**	**%correct Netherlands**	**Difference Belgium/ Netherlands**
aangringen	91.8	92.8	-1
aanjonterlijk	93.7	95.5	-1.8
aanschagenen	90.6	91	-0.3
aarberheid	96.1	94.4	1.6
abkupt	97.4	97.5	-0.1
ablociltuur	92.5	92.2	0.3
adaandes	97.1	97.1	0
adpront	95.2	94.1	1.1
adukreker	96	97.4	-1.4
adwanderen	96.7	96.3	0.4
afbas	94.3	92.4	1.9
afgerstrezen	97.4	97.7	-0.2
afrepent	92.2	94.6	-2.4
agsinent	95.6	95.2	0.4
agumotie	91.3	90.9	0.4
akeer	90.6	93.5	-2.9
aketolief	93.5	92	1.6
akitet	94.9	97.9	-3
akkerief	95.3	95.3	0
aktheminaren	90.8	92.7	-1.9
alapilitie	91.7	94.3	-2.6
algeziet	92.3	91.7	0.7
alortstoding	97	97	0
amektel	96.6	96.2	0.3
ampekestigd	97.4	97.4	0
ampetwinsbaar	96.4	97	-0.6
amunt	92.5	91	1.5
amusazist	93.2	90.4	2.8
andstopt	97.2	97.3	-0.1
angeideling	96.3	96.2	0.1
angraar	93.2	94.1	-0.9
anschorp	96.1	97.2	-1
apennatig	92.6	92	0.6
aperuild	97.4	97.8	-0.4
aplordigen	97.7	96.6	1.1
apmuselief	97.8	96.3	1.5
aprennief	94.5	94	0.5
arachteleem	94	95.9	-2

arfoezelen	93.4	95	-1.6
artilef	93.2	92.8	0.4
aruindig	94.2	92.9	1.3
asdanonaal	92.2	95.5	-3.3
asdilaal	90.5	92.3	-1.7
asseldig	96.2	96.7	-0.6
astemeeslijk	95.8	95.2	0.6
asteraards	90.1	93.8	-3.7
astienen	91.8	96.4	-4.6
atalsmaal	97	96	1
atendueen	92.3	93.3	-1
aterboren	96.4	96	0.4
atsel	95.2	93.5	1.7
attikt	96.5	95.5	1
awanteur	92.8	95.7	-3
bajoktrant	95.3	93.1	2.3
ballelliek	94.4	92.5	1.9
bameen	96.8	95.4	1.4
bamitoseren	90.4	94.4	-4
banstelijk	94.3	94.3	0
basrikton	97.6	96.9	0.7
bastarheid	94.3	96	-1.7
bebriepig	95.5	96	-0.5
bechts	96	96.5	-0.5
bedrannik	96.3	97.5	-1.2
begildig	94.1	91.8	2.3
bekattupeerd	94.4	94.1	0.3
bekelijk	93.3	91.7	1.6
bekeutoging	96.4	96	0.4
belonzelijk	96.3	94.9	1.4
bemokend	90.4	93	-2.6
bendasta	96.5	97.1	-0.6
benebbel	96	91.9	4.1
bepeudigen	94.9	96.6	-1.7
berantweerdeteit	97.1	97.5	-0.3
berdeeft	96.5	96.6	-0.2
berdemd	96.7	97.7	-1
berdomelen	95.9	97.3	-1.4
berennelen	92.7	95.7	-3
berhakkening	94.4	94.8	-0.5
berkaaien	92.1	90.1	2
berkleiten	94.8	96.1	-1.3
berkluitig	91.8	92.4	-0.6
berknelpen	94.2	95.7	-1.5
berkoer	92.6	94.1	-1.5
bermaring	97.6	97.3	0.2
bermegen	96	96.7	-0.8
bermoring	95.5	97	-1.5
bermurmen	96	94.7	1.3
berobaren	94.5	96.2	-1.6
berrillelijk	97.5	91.7	5.8
berschieking	96.8	97.4	-0.6
berspreuking	93.7	93.1	0.6

berstraaid	94.5	94.4	0.1
berveugd	96.6	94.8	1.7
bervichten	96.8	97.2	-0.5
berzieging	95.6	96	-0.3
beschreeg	91.5	90.8	0.8
besleek	91.9	90.8	1.1
bespreizing	94.3	94.3	0
besprering	94.3	91.8	2.5
betijring	90	90.2	-0.1
betrallel	96.7	95	1.7
bieuw	95.6	96.4	-0.8
bijbarting	91.6	93.7	-2.1
bijtole	96.4	96.8	-0.5
bladetig	96.4	94.4	2
blankelen	92.8	90.5	2.3
blanstig	91.7	95.6	-3.9
bleetbaar	97	97.4	-0.4
bleubel	95	95.1	-0.1
bloefpe	97.3	97.6	-0.3
blort	96	96.6	-0.6
bodervlaad	94.5	96.8	-2.2
bolsode	93	94.9	-1.8
boltpomier	93	96.3	-3.3
braar	93.6	93.3	0.3
brapering	96.9	96	0.9
brapla	97.4	97.3	0.1
breep	95.1	94.9	0.2
brelder	95.5	96.2	-0.8
broeg	90.5	93.7	-3.1
brog	91.8	91.8	0
brongen	93.1	95	-1.8
broorroos	97.3	97.1	0.2
calalect	92.4	93.7	-1.3
callepaneel	91.3	92.1	-0.7
castroek	94.7	95.6	-1
coddactie	90.9	95.2	-4.3
comgemetie	93.7	94.4	-0.7
congildie	95.6	93.4	2.1
conjentralen	92.4	95.4	-3
contallaren	95.1	94.2	0.9
contokistaal	90.6	94	-3.4
contrellaren	95.7	95.2	0.4
corbemitie	93.1	92.7	0.4
corlesitie	91.5	91.5	-0.1
corrublindent	91.4	94.6	-3.2
corstorte	93.2	95.1	-1.9
cougeise	96.5	94.8	1.7
daargemmenin	97.2	97.1	0.1
darfect	96.1	96	0.1
dargel	95.1	93.7	1.4
dasken	93.3	93.5	-0.2
debivet	90.8	92	-1.1
degisch	91.1	92.4	-1.3

deharstructie	93.5	92.3	1.2
dehomueel	95.6	95.1	0.5
delinkpandel	97.2	97	0.1
demaans	90.1	92	-1.9
derebas	95	93.9	1.1
derultor	94.2	96.9	-2.7
desakentie	94.4	97.5	-3.1
deskelijk	96.5	97	-0.5
detig	91.7	93.4	-1.6
devensbiel	97.1	97.2	-0.1
dierdig	91.7	90.9	0.9
dimestolieel	90.6	93.7	-3.1
dimor	91.4	96.4	-5
dochtkrij	94.1	95.3	-1.2
dofikotief	93.7	96.2	-2.5
doleton	94.5	93	1.5
domibel	90.7	94.9	-4.2
dookels	97.2	97	0.1
doornarstoken	93.4	95.4	-2
doorwijns	91.4	92.3	-0.9
drant	91.3	95.7	-4.4
drapolen	92.3	92.8	-0.5
drorzeloos	96.5	96.9	-0.4
duiketing	96.8	96	0.8
dunileins	96.5	97.2	-0.6
dymkactisch	92.1	96.3	-4.2
efject	96.3	94.6	1.6
egenweist	97.5	96.4	1
eggrotie	91.1	93.6	-2.5
egknotisch	96.6	95.3	1.3
eiltede	94.5	96.7	-2.2
ekaldiek	91.2	94.2	-3
ellepitie	92.4	94.1	-1.7
ematintatie	92.4	93.4	-1.1
embabron	90.9	91.2	-0.3
enturmen	90.1	95.1	-5
enzap	97.8	97	0.8
erkarts	94.2	96.6	-2.4
eukereed	95.9	97.4	-1.5
fabroet	95.4	96.7	-1.2
fafial	95.6	96.4	-0.8
fagawel	96.1	96.3	-0.2
faliliedeen	93	94.1	-1.1
feis	92.5	93.7	-1.2
figuis	90.4	92.4	-2
filfiet	95.2	92.8	2.4
fiteek	94.2	97.7	-3.5
fitgeraal	94	95.4	-1.4
fitken	96.5	97.3	-0.8
flazapeut	94.2	95.7	-1.6
flepelijk	94.4	97	-2.6
flepen	91.9	94.4	-2.5
flibbel	94.5	93.5	0.9

flonstelen	93.2	95.7	-2.5
flonstelend	93	94.2	-1.2
flusegen	95.6	97.7	-2.1
fluwaag	94.1	95.5	-1.4
fomiheer	97.5	95.6	1.9
fortorij	92	92.3	-0.3
fragteur	95.1	90.6	4.5
frideurlet	95.9	96.2	-0.2
frimekel	95.2	95.5	-0.3
fririlen	95.5	95.9	-0.4
fupieus	92.6	92.6	0
gadatast	97.8	96.8	1
ganriteur	95	91.8	3.2
gavimaal	90.2	92.3	-2.1
gebantie	91.3	95.4	-4.1
gebecht	93.5	91.1	2.4
gebruursheerd	93.6	94.6	-1
geelkebigen	97.8	97.1	0.7
gekraad	92.5	90.9	1.6
gelamersine	91.2	93.8	-2.6
geloorderaad	90.6	93.5	-2.9
gemaaf	95.3	96.9	-1.6
gemoeitoedig	97.2	97.7	-0.5
geneetgemdel	97.2	97.6	-0.4
gepoping	92.4	96.3	-3.9
gepromen	96.5	96.1	0.4
gerbongen	96.7	96.2	0.4
gerdel	91.6	92.3	-0.7
gerschuikele	97.5	97.3	0.2
gersovelaar	95.2	97.8	-2.5
gerstordig	95.6	95.3	0.3
gertiesterers	96.7	96.7	0.1
gerulbel	95.9	96.4	-0.5
gescheuste	93.9	90.8	3.1
geschiend	93.5	92.3	1.2
gesigaraseerd	94	92.8	1.2
gespenken	93.1	90.9	2.3
gestensen	93.5	94.3	-0.7
getwijnel	90.8	94.2	-3.4
geuist	97.6	94.7	2.9
gevina	93.6	96.8	-3.2
geweintedis	97	97.2	-0.2
gezerpen	90.4	94.2	-3.8
gigkruchtig	95.3	97.8	-2.4
glappelig	95	92.5	2.4
glarsuis	95.5	97.7	-2.2
glippant	90.1	94.7	-4.6
glistelijk	93.3	95.9	-2.6
grammooien	95	95.4	-0.4
grink	90.6	90.8	-0.1
groeking	96.5	94.2	2.2
gruleristisch	93	95.1	-2.2
guitak	93.3	93.7	-0.4

gulpontig	91.6	96.5	-4.9
gussiraal	92	96.5	-4.5
haispisse	97.4	97.3	0.1
halboeglijk	92	94.7	-2.7
halgepollig	96	97	-1
halnomen	91.6	93.5	-1.9
hekatie	92.3	91.4	0.9
heltelijk	94.1	95.5	-1.4
hentin	95.4	96.6	-1.2
hepeloos	97.1	94	3
herklefel	96	97.2	-1.2
hermeerleter	97.3	97.7	-0.4
hetetiekorps	92	92.2	-0.2
heunel	91.8	93.1	-1.3
himpig	90.7	96.4	-5.7
hokelal	97.6	96.3	1.3
holalen	95.5	96.3	-0.8
horicijn	90.9	94.3	-3.3
horps	91.8	96.7	-4.9
hutaar	92.5	97.4	-4.9
iddimeren	94.1	95.4	-1.2
ijfling	90.4	90.3	0.1
iklap	97.9	97.1	0.8
imbing	97.6	95.5	2.1
immagaal	91	90.2	0.8
inbricetie	90	92.5	-2.5
indokstatetie	94.4	94.2	0.2
infilrie	95	93.6	1.4
infost	90.3	96.5	-6.2
inkichtig	94.6	96.3	-1.7
inkouken	97.2	97.8	-0.6
inonfie	92.6	96.2	-3.6
insloeding	95.7	94.1	1.5
inspate	95.3	95.9	-0.5
instennaren	94.2	94.2	0
intabavueel	97.1	96.5	0.6
inteldaag	94.5	96.3	-1.8
intilm	96.7	97.2	-0.5
intotovitie	96	97.4	-1.3
intuileinend	95.6	96.6	-1
invensiptie	90	93	-3
invenstordig	96.6	96.4	0.2
invisting	91.8	93.5	-1.7
invoekelijk	91.8	95	-3.2
inwartief	95.2	96.1	-0.8
iriel	92.7	90	2.7
isenkatie	97.8	96.1	1.7
jagogdictie	95.6	94	1.6
jagradieel	95.6	92.2	3.4
jamier	94.2	95.1	-0.9
jangerenkift	97.4	97.5	-0.1
jarloficaat	92.5	93.8	-1.3
joddegrok	95.1	95.7	-0.6

jomisch	91	95.1	-4.1
jontast	97.2	96.4	0.8
judrulant	92.5	94.8	-2.2
kadelziek	90.9	95.8	-4.8
kalbelade	93.3	94.9	-1.6
kalzelen	91.8	95.2	-3.4
kangegen	97.5	97.2	0.3
kardidieus	90.7	92.4	-1.7
kavree	93.4	96.8	-3.5
kegemant	96.1	95	1.2
kienelig	93.1	92.8	0.4
kieneling	92.1	91.9	0.2
kienstbaal	93.3	95.3	-2
kigneneris	97.2	97.6	-0.4
kiksel	92	93.8	-1.8
killamolatie	90.3	95	-4.7
kirgedier	95.9	94.3	1.6
klampigaal	92.6	93.5	-0.9
klergte	95	97.4	-2.4
knam	96.4	97.4	-0.9
koddenhoor	96.9	96.1	0.7
koefmobberij	95.9	97.8	-1.9
koermink	91.8	93.7	-1.9
kojoluut	93.5	94.7	-1.2
konbijleut	97.7	97.6	0.1
kondlerstig	97.9	97.3	0.6
kondreiten	93	92.6	0.4
konnelig	92.5	93.9	-1.5
korculien	94.4	95.4	-1
korket	91.6	93.8	-2.1
kosenteur	96.2	96.4	-0.2
kotaal	92.7	93.7	-1
kovetie	92.7	94.4	-1.6
krachteteel	91.9	92.2	-0.3
krepadetie	92.4	95.1	-2.8
krocuraas	96.1	97.3	-1.2
kroden	92.5	92.8	-0.3
kroren	96.1	97.3	-1.2
krussig	92.9	92.6	0.3
kwetelijk	91.8	91.6	0.1
lagelen	94.5	91.9	2.6
legroperiseren	93.3	93.8	-0.5
lekinklijk	97	97	0
lepenuit	94.7	96.2	-1.6
lepeven	97.3	97.1	0.1
lestrundie	95.6	96	-0.4
liepenpasier	97	97.3	-0.3
ligittie	92.3	96.4	-4.1
lilikent	97.2	96.6	0.6
lioleit	92.8	94.6	-1.9
lirultief	91.5	93.1	-1.6
loerna	96.2	96.4	-0.2
lolict	96.4	95.9	0.6

lomguasief	94.6	96.7	-2.1
lorfararen	97.1	97.8	-0.7
lozeraal	92.6	94.6	-2
lulstbandig	93.1	97.2	-4
lurdig	92.8	94.1	-1.3
maarmarstig	91.5	93.6	-2
mabikaal	90.7	96.3	-5.6
mamiergrem	97.8	97.7	0.1
mandon	90.2	91.9	-1.7
mandvarrijd	97	97.7	-0.7
maneetdoen	97.9	97.7	0.1
manijn	91.7	96	-4.4
mardon	92.8	95.5	-2.7
marsens	93.8	94.7	-0.9
mazein	93.4	93.4	0.1
medormap	91.5	95.1	-3.5
mempen	93.4	92.7	0.8
menlariteit	95.3	95.2	0
mentekid	97.1	97.4	-0.3
mererist	91.9	90.6	1.3
merraguur	94.3	95.6	-1.3
messtaad	92.1	94.1	-2
miakinaal	91.9	93.8	-1.9
mialaltiek	92	95.5	-3.5
michten	93.9	93.9	0
miklenatisch	96	95.8	0.2
mipel	92.6	95.2	-2.6
mobbijn	95.8	95.5	0.3
moetel	96.5	93.4	3.1
momelons	97.2	97.6	-0.4
mopcurn	97	97.6	-0.6
mosimaal	94.1	93.2	0.9
muikdelijk	96.4	96.9	-0.6
mukiek	96.6	96.9	-0.3
mupline	93.2	96.3	-3.1
muzegen	94.3	95.7	-1.4
nacef	97.4	97.1	0.4
nachteraan	91.5	94.3	-2.8
namerijn	93.6	94.8	-1.2
naromender	94	94.5	-0.5
nartelijk	91.7	97.6	-5.9
natcoom	97.2	97	0.2
navanning	95.5	97.7	-2.2
neekrag	97.1	97.4	-0.3
neharber	97.5	97.7	-0.1
nekeven	97.4	96.3	1.1
nemenraal	96	93.2	2.8
nenteking	97.7	97.7	0
neuroek	95.6	97.6	-2
nidasteren	91.4	95.2	-3.8
nokaar	92.7	92.9	-0.2
obbeschegt	97.6	96.7	0.9
odenhuiten	97.5	97	0.5

odenwuitang	97.4	97.8	-0.4
oehamen	96.1	96.6	-0.5
ofslijkbaar	95.9	97	-1.1
okstobutie	95.6	96.5	-0.9
olbangs	96.7	97.5	-0.8
olletude	93.6	96.4	-2.9
olwafolisch	95	97.3	-2.3
ompuchtbaar	91.6	94.7	-3
omvaan	94.4	93.5	0.9
omvarvorsen	97	97.3	-0.2
omvoelaan	96.1	97.6	-1.6
ongertief	95.8	93.8	1.9
onlongellijk	95.1	96.7	-1.6
onnerwande	95.6	95.5	0.1
onrolen	94.2	94.4	-0.2
onstronen	94.8	95.3	-0.5
onterweven	94.1	93.4	0.8
onterziemen	96.4	95.2	1.1
ontlertenen	97.7	97.6	0
ontzuppen	92.3	94.2	-1.9
onveschremen	94.8	94.4	0.5
onvoger	96.3	97	-0.7
oordiet	90.8	90.2	0.6
opana	94.1	93.9	0.2
opbino	97.7	97.9	-0.2
opgakerij	93.3	95.9	-2.6
opongelen	91.8	94.6	-2.8
oppebeuglijk	96.3	94.3	2
opprinee	93.5	95.8	-2.3
oppumaren	95.5	95.3	0.3
oranfafeet	94.4	97.7	-3.3
osmeling	95.3	95.4	0
ospassedeur	96.7	95.4	1.4
ostemarkt	91.8	93.1	-1.3
otij	94.1	95.9	-1.8
otlikisme	92.2	94.9	-2.7
ouwacieten	94.9	96.6	-1.7
overmifis	97.7	97.8	-0.1
padelstiten	97.1	96.8	0.2
pamend	90.6	93.2	-2.5
pammerd	94.7	93.9	0.7
papijl	94.4	93.1	1.2
paud	97.4	97.8	-0.4
peject	94.1	96.6	-2.5
pensiet	92.1	90.6	1.5
pideletuur	92.4	93.3	-0.9
pigiek	93.1	94.9	-1.8
pigulder	97.3	97.6	-0.3
pihengiaat	93.7	97.3	-3.6
pijtens	96.3	94.7	1.7
pinterlaarlijk	91.8	95.9	-4.1
plaricaat	91.4	90.7	0.7
plisterig	92.5	92.3	0.2

pluwelaan	92.4	93.2	-0.8
poenbouber	97.1	97.3	-0.1
pokenin	94.2	96.7	-2.5
pondoes	90	90.1	-0.2
pongeloos	90.3	95.6	-5.3
porkon	95.3	96	-0.7
poventig	94.5	94.6	-0.1
prenilaren	90.7	93.8	-3.1
pretebentie	90	96.4	-6.4
pribuur	96.1	97.2	-1
prikezie	92.2	93.5	-1.3
prodoet	97	96.4	0.6
progeceteur	93.2	92.6	0.6
propikkeron	93	95.3	-2.3
protridenade	91.2	91.8	-0.6
pulstueel	90.1	91.6	-1.5
puvide	90.1	91.9	-1.8
rakwan	93.6	95.8	-2.2
rallibule	90.5	92.7	-2.2
ralm	90.1	91.1	-1
ramarij	93	95.1	-2.2
ramenin	94.9	94.6	0.3
ramfe	97.5	97.7	-0.2
rebrijk	94.4	97.3	-2.9
reddine	91.8	95.4	-3.6
rehotoren	95.2	94.5	0.7
rerditie	95.7	95.2	0.5
restuinenswaardig	94.5	91.8	2.7
reuperd	91.2	92.2	-1
revost	93	95	-2
riepoedig	94.6	95.7	-1.1
rilomode	92.9	96.6	-3.7
riodist	90.9	93.1	-2.2
roebiel	93.4	96.2	-2.8
roefdra	95.4	97.4	-2
roetorre	95.8	96.9	-1.1
rolk	91.6	95.1	-3.5
rolpstarrig	95.2	94.3	1
roortrig	95.9	96.4	-0.5
rordoyant	94.4	95.2	-0.8
rorio	96.7	96.4	0.3
ruimkreging	94.8	93.9	0.9
rummij	96.9	96.9	0
schepting	96.4	96.2	0.2
sches	97.1	95.6	1.6
schesks	97.1	97.1	0
schesser	95.1	96.9	-1.8
schieluin	93.6	90.2	3.4
schijgste	96.8	97	-0.2
schobbalen	91.6	92	-0.4
schobeting	93.8	97.1	-3.4
schokeldijn	92.1	96.6	-4.5
scholotisch	92.7	93.1	-0.4

schossig	91.1	91.5	-0.4
schroezer	90.6	94.3	-3.7
scruputoel	96.3	95.9	0.4
sefpor	97.3	97.8	-0.5
sietsel	95.2	93.7	1.4
sinuragen	94.6	94.9	-0.4
slaarmekker	94.5	97.1	-2.6
slaarpol	96.4	97.7	-1.4
slanspalent	95.7	96.8	-1.1
slapioloog	94	91.5	2.5
smakteel	93.3	95.4	-2.1
smapmatisch	95.4	95.8	-0.3
smoebaan	96	96.5	-0.6
snemmer	96.3	96.8	-0.6
sonspering	92.8	92.3	0.4
soorteraan	94.8	93.6	1.2
sormies	90.9	96.2	-5.2
soscaal	95	96.8	-1.8
speesing	95.7	93.6	2.1
spipper	96	96.2	-0.2
splimpen	91.8	94	-2.2
sprans	94.3	91.5	2.8
spropen	95	97.4	-2.4
spropend	95.2	93.8	1.4
spruichen	96.1	94.3	1.8
stadderaar	91.4	93.6	-2.2
stakrast	92.3	91.7	0.6
staliam	94.9	94.4	0.6
stessig	90.4	93.3	-2.9
steupem	96.1	96.3	-0.1
stiellijs	91.8	94.4	-2.6
strissen	90.5	92.9	-2.4
strokon	94.7	94.1	0.6
stroon	91	91.5	-0.5
studiediet	91.7	93.3	-1.5
tadeboe	97.7	96.5	1.3
tagisor	95.6	96.1	-0.6
taslon	91.7	94.5	-2.8
tecastentie	93	91.8	1.2
tederieden	95.2	95.7	-0.5
telbioen	95.7	94.8	0.9
temifrase	90.4	90.1	0.3
teramikratie	90	91.6	-1.5
theoraak	93	90.4	2.6
tibbegen	95.9	97	-1.1
tidrolen	95.4	96.9	-1.5
tienenis	97.8	97.3	0.6
tijmant	94.3	96.7	-2.4
tijvinigheid	91	95.4	-4.3
tinpeloos	92.1	94.2	-2.1
toeptelig	96.9	96.4	0.5
tokedoni	96.8	97.5	-0.7
topelig	92	93.4	-1.5

torgegraads	96.6	96.3	0.2
toteateur	97	97.8	-0.8
traar	94	94	0.1
tralaan	91.6	92.1	-0.4
trandellien	91.5	95.7	-4.3
trarrofoon	90.9	94.9	-4
traten	93.7	93.8	-0.1
trekkaat	91.8	92.8	-0.9
tretting	92.4	92.6	-0.2
trewen	95.4	96.5	-1.1
trirvioen	92.4	95.2	-2.8
tronsttijn	96	96.2	-0.1
truma	94.1	94.8	-0.7
tups	91.2	91.8	-0.6
tweelerkjus	97.1	97.3	-0.2
twikkig	92.4	91	1.4
uitklesting	93.3	94.8	-1.5
uitklorsel	95	96.3	-1.4
uitwok	91.2	91.6	-0.4
ukeef	97.3	97.4	-0.1
umotoren	90.3	92.8	-2.5
vaatstonzel	90.1	93.7	-3.6
valdrongen	91.3	94.2	-2.9
valtewas	96.4	97.1	-0.7
vebeerder	96	96.9	-0.9
vebierding	96.2	95.5	0.7
vecindeerd	94.1	93.4	0.7
vedalen	95.2	94.3	0.9
vegachtspunt	96.1	96.3	-0.2
veheitend	96.9	96.4	0.5
vehoutelt	97.3	97.7	-0.4
vejaarlenhaad	97.4	97.6	-0.2
vekeeuwen	96.9	97.9	-1
veleet	93.6	94.7	-1.1
vemechten	95.9	95.6	0.3
vemekken	93.7	95	-1.3
venterm	92.3	93.9	-1.6
veoorlinaar	94.8	97.7	-2.8
veraand	92.7	92.8	-0.1
verbrelen	91.6	91.7	0
verdaspen	92	95	-3
vereberen	93.3	93.2	0.1
vereberend	92.3	94.9	-2.6
vereuben	95.4	97.7	-2.3
verisserig	93.2	97.8	-4.6
verneertezen	97.4	97.8	-0.3
verroppend	95	90.1	5
versaander	95.3	95.9	-0.6
verspreigen	93.3	94.5	-1.2
vervarden	91.7	93.3	-1.6
vervarding	90.9	93.7	-2.8
verwolper	91.9	95	-3.1
vescheligd	95.7	95.5	0.3

vescheming	94.2	96.7	-2.5
veschogbaar	96.1	96.3	-0.3
veschosten	97.6	97	0.5
veschosting	95.9	93.4	2.5
veslammen	96.1	96.9	-0.8
vetactisch	91.5	92.5	-1
vetadiel	94.8	93	1.8
veteggelijk	94.7	97.1	-2.4
vetichten	95.8	94.6	1.2
vewoeg	96	97.1	-1.1
vezaren	93.3	94.5	-1.2
vezeid	97.1	97.8	-0.6
vezerting	93	95.7	-2.7
viarageren	90.5	92.6	-2.1
vinneljaart	96.1	97.9	-1.8
viriowaald	95.8	96.4	-0.6
virmieel	93.3	93.7	-0.4
vivoel	94.1	94.2	-0.1
vlum	92	94.8	-2.8
voeping	97.6	96.5	1.1
voeriek	96.4	94.3	2.1
vollundig	94.4	92.5	1.8
volstutig	92.8	92.4	0.4
vontjat	97.6	97.8	-0.2
vorris	92.9	95.9	-3
vory	97.2	97.4	-0.1
voseraar	92	92.9	-0.9
vrallen	97.4	97	0.4
vrebaal	95.2	95.9	-0.7
vrijslerieus	97.4	97.6	-0.2
vrijvopig	97.1	97	0.1
vrunteling	93.2	96.8	-3.6
waanderijks	92.2	93.6	-1.4
waarbijn	93.9	97.2	-3.3
wacromeren	94.1	94	0.1
wadeborker	97.7	96.8	0.9
wanmentine	94.6	96.2	-1.6
wanositie	95.6	96.5	-0.9
warmunetie	92.9	94.1	-1.2
warralist	92.8	96.1	-3.3
waspografie	90.6	92.8	-2.3
wedrokoom	95.7	97.5	-1.8
weelslaad	96.9	96.8	0
welsilaat	94.6	96.6	-2
werium	92.2	94.1	-2
wijpeloegen	97.2	97.3	-0.1
wimming	94.5	95.4	-0.8
wingstondig	93	91.6	1.4
wrijmeraar	93.3	97	-3.7
wutlen	97.2	97.5	-0.3
zetidisch	91.8	93.9	-2.1
zijketig	94.5	92.5	2
zossue	97.1	97.8	-0.7

zuimnatuatie	95.4	97.7	-2.2
zuitend	91.8	93	-1.3

2. Peabody picture vocabulary test

Example trial:

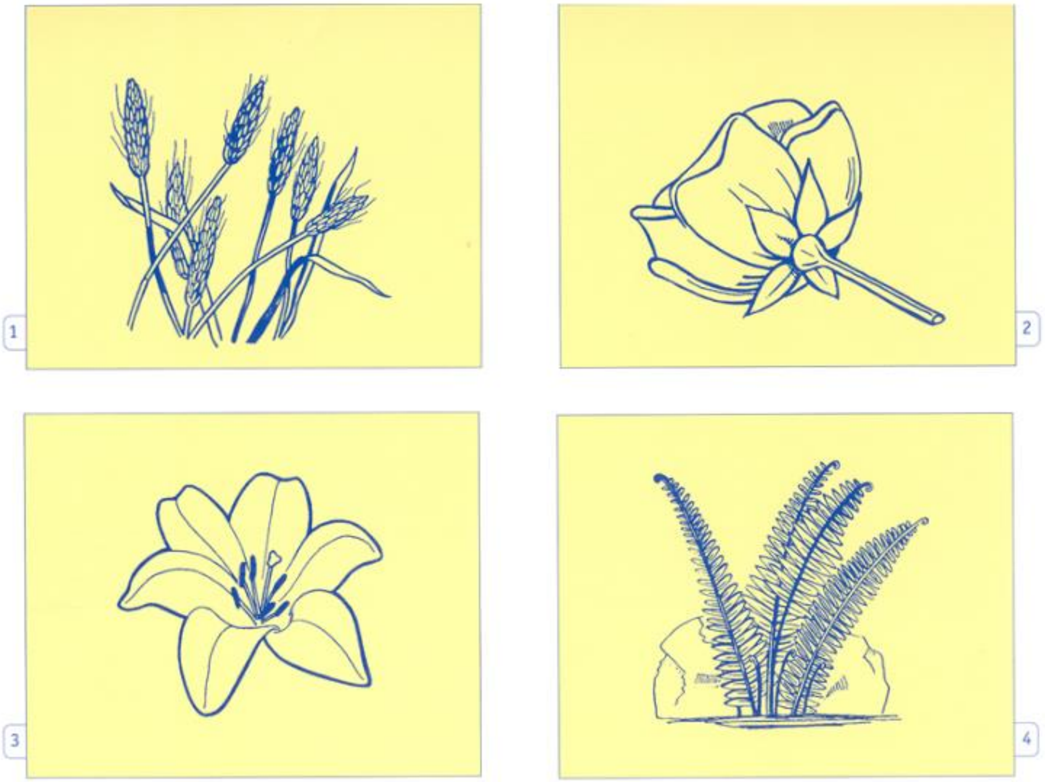

"Druk op spatie om het woord te horen. Klik op het plaatje dat bij het woord hoort." (Press the spacebar to hear the word. Click on the picture associated with the word.)

Audio: "Meeldraad" (stamen)

3. Spelling test

Word	Correctness	Correct spelling	Category	ZipF	Prevalence
kauwgom (chewing gum)	correct	-	au/ou	3.99	0.99
akkoord (agreement/accord)	correct	-	c/k + letter doubling	2.43	1.00
februari (February)	correct	-	capital + end-i	3.78	1.00
geliefkoosd (carressed)	correct	-	d/t	1.7	0.95
litteken (scar)	correct	-	d/t	3.26	0.99
dreumes (toddler)	correct	-	e/u	2.51	0.99
allerlei (all kinds of)	correct	-	ei/ij	2.36	1.00
refrein (chorus)	correct	-	ei/ij	3.06	0.99
eindhalte (final stop)	correct	-	ei/ij + d/t	2.26	0.99
discipline (discipline)	correct	-	knowledge	3.92	1.00
feliciteren (congratulate)	correct	-	knowledge	3.82	1.00
hypnose (hypnosis)	correct	-	knowledge	3.42	0.99
interview (interview)	correct	-	knowledge	4.22	0.99
jaloezie (jealousy)	correct	-	knowledge	3.78	1.00
pyjama (pajamas)	correct	-	knowledge	3.77	0.99
succes (success)	correct	-	knowledge	5.02	1.00
ananas (pineapple)	correct	-	letter doubling	3.4	0.99
apparaat (device)	correct	-	letter doubling	4.25	0.99
initiatief (initiative)	correct	-	letter doubling	3.68	1.00
kannibaal (cannibal)	correct	-	letter doubling	3.01	1.00
ontplooiing (unfoldment)	correct	-	letter doubling	1.95	n/a
onverbiddelijk (inexorable)	correct	-	letter doubling	2.48	0.99
personage (character)	correct	-	letter doubling	3.86	1.00
hondenhok (doghouse)	correct	-	n	2.89	1.00
papegaai (parrot)	correct	-	n	3.52	0.99
bacteriën (bacteria)	correct	-	plural-ie/trema	3.58	0.98
hoofdstad (capital)	correct	-	silent letter + within-s	3.81	1.00
afkickcentrum (rehab center)	correct	-	single word	1.85	-
blindedarmontsteking (appendicitis)	correct	-	single word	2.43	-
sympathie (sympathy)	correct	-	t/th	2.04	1.00
ternouwernood (narrowly)	incorrect	ternauwernood	au/ou	2.57	0.99
reklame (advertisement)	incorrect	reclame	c/k	4.11	1.00
gefotokopieërd (photocopied)	incorrect	gefotokopieerd	c/k + trema	1.7	n/a
neerwaards (down)	incorrect	neerwaarts	d/t	3.77	0.99
onbemint (unloved)	incorrect	onbemind	d/t	2.2	0.99
stiekum (secretly)	incorrect	stiekem	e/u	4.07	0.99
chagerijnig (grumpy)	incorrect	chagrijnig	ei/ij	3.54	0.96
mislijdend (misleading)	incorrect	misleidend	ei/ij	3.01	1.00
porselijn (china)	incorrect	porselein	ei/ij	3.26	1.00

alinia (paragraph)	incorrect	alinea	i/e	2.72	0.99
joghurt (yogurt)	incorrect	yoghurt	knowledge	3.33	1.00
licensie (license)	incorrect	licentie	knowledge	3.19	0.99
nivo (level)	incorrect	niveau	knowledge	4.4	1
sollisiteren (apply)	incorrect	solliciteren	knowledge	3.35	1
spagetti (spaghetti)	incorrect	spaghetti	knowledge	3.83	0.99
aggressie (aggression)	incorrect	agressie	letter doubling	3.7	0.99
anuleren (cancel)	incorrect	annuleren	letter doubling	3.54	0.99
brocoli (broccoli)	incorrect	broccoli	letter doubling	4.28	0.99
diaree (diarrhea)	incorrect	diarree	letter doubling	3.6	0.99
onmiddelijk (immediately)	incorrect	onmiddellijk	letter doubling	4.69	0.99
sinasappelsap (orange juice)	incorrect	sinaasappelsap	letter doubling	3.43	0.98
slimmerikken (geniuses)	incorrect	slimmeriken	letter doubling	4.37	n/a
eigelijk (actually)	incorrect	eigenlijk	n	5.49	1
waardenloos (worthless)	incorrect	waardeloos	n	4.4	1
caloriën (calories)	incorrect	calorieën	plural-ie/trema	3.06	1
tennister ((female) tennis player)	incorrect	tennisster	s + letter doubling	1.95	n/a
muzeum (museum)	incorrect	museum	s/z	4.55	1
lage drukgebied (low pressure area)	incorrect	lagedrukgebied	single word	2.43	n/a
vantevoren (in advance)	incorrect	van tevoren	single word	-	n/a
autentiek (authentic)	incorrect	authentiek	t/th	2.97	1

Note: ZipF = Zipf frequency.

4. Author recognition test

Name	Code	Difficulty	Item.Tot.woi	Item.Rel.woi	Discrim
AFTh Van der Heijden	AUT	0.28	0.57	0.26	0.59
Agatha Christie	AUT	0.78	0.21	0.09	0.32
Anne Provoost	AUT	0.6	0.34	0.17	0.53
Annet de Jong	AUT	0.1	0.46	0.14	0.24
Annie MG Schmidt	AUT	0.83	0.36	0.14	0.36
Arnon Grunberg	AUT	0.38	0.53	0.26	0.59
Astrid Lindgren	AUT	0.42	0.51	0.25	0.67
Bart Moeyaert	AUT	0.66	0.37	0.17	0.52
Bertolt Brecht	AUT	0.42	0.25	0.12	0.32
Carlos Ruiz Zafón	AUT	0.2	0.45	0.18	0.39
Cees Nooteboom	AUT	0.3	0.6	0.27	0.58
Charles Baudelaire	AUT	0.39	0.39	0.19	0.53
Connie Palmen	AUT	0.19	0.53	0.21	0.42
Dan Brown	AUT	0.78	0.4	0.17	0.5
Daniel Mason	AUT	0.15	0.58	0.21	0.38
Dante Alighieri	AUT	0.24	0.45	0.19	0.42
David Baldacci	AUT	0.2	0.6	0.24	0.47
David Grossman	AUT	0.17	0.6	0.22	0.39
Dimitri Verhulst	AUT	0.76	0.33	0.14	0.38
Donna Tartt	AUT	0.21	0.46	0.18	0.39
Elizabeth George	AUT	0.23	0.54	0.23	0.47
Emily Brontë	AUT	0.41	0.49	0.24	0.59
Fay Weldon	AUT	0.1	0.58	0.17	0.27
Gabriel Garcia Márquez	AUT	0.44	0.39	0.19	0.52
George Eliot	AUT	0.4	0.55	0.27	0.68
Georges Simenon	AUT	0.15	0.46	0.16	0.33
Guy de Maupassant	AUT	0.3	0.48	0.22	0.47
Harper Lee	AUT	0.24	0.44	0.19	0.44
Harry Mulisch	AUT	0.52	0.38	0.19	0.48
Helen Fitzgerald	AUT	0.39	0.52	0.25	0.64
Henning Mankell	AUT	0.15	0.45	0.16	0.3
Herman Brusselmans	AUT	0.9	0.16	0.05	0.09
Herman Koch	AUT	0.43	0.59	0.29	0.7
Hermann Hesse	AUT	0.23	0.52	0.22	0.48
Hubert Lampo	AUT	0.22	0.55	0.23	0.45
Isabel Allende	AUT	0.22	0.54	0.22	0.45
James Patterson	AUT	0.32	0.42	0.2	0.44
Jane Austen	AUT	0.61	0.36	0.17	0.45
JD Salinger	AUT	0.31	0.44	0.2	0.45
Jean M Auel	AUT	0.21	0.49	0.2	0.44
JJ Voskuil	AUT	0.2	0.55	0.22	0.48

JK Rowling	AUT	0.94	0.21	0.05	0.12
JM Coetzee	AUT	0.28	0.41	0.18	0.45
John Grisham	AUT	0.3	0.47	0.21	0.47
John le Carré	AUT	0.16	0.56	0.2	0.42
Joris van Casteren	AUT	0.12	0.57	0.18	0.32
JRR Tolkien	AUT	0.69	0.29	0.13	0.47
Kader Abdolah	AUT	0.2	0.58	0.23	0.5
Karin Slaughter	AUT	0.33	0.4	0.19	0.45
Khaled Hosseini	AUT	0.49	0.3	0.15	0.47
Kristien Hemmerechts	AUT	0.42	0.44	0.22	0.5
Lulu Wang	AUT	0.23	0.46	0.19	0.41
Manon Uphoff	AUT	0.12	0.61	0.2	0.32
Marc De Bel	AUT	0.94	0.19	0.04	0.09
Margaret Atwood	AUT	0.3	0.59	0.27	0.65
Marianne Fredriksson	AUT	0.16	0.47	0.17	0.36
Mario Vargas Llosa	AUT	0.12	0.5	0.16	0.33
Marion Pauw	AUT	0.12	0.55	0.18	0.29
Mark Twain	AUT	0.42	0.47	0.23	0.55
Michael Connelly	AUT	0.21	0.57	0.23	0.5
Miguel de Cervantes	AUT	0.35	0.53	0.25	0.59
Milan Kundera	AUT	0.21	0.44	0.18	0.38
Nicci French	AUT	0.6	0.29	0.14	0.33
Patricia Cornwell	AUT	0.41	0.49	0.24	0.58
Per Olov Enquist	AUT	0.1	0.51	0.15	0.26
PF Thomése	AUT	0.13	0.67	0.22	0.36
Pieter Aspe	AUT	0.92	0.2	0.05	0.12
Ray Bradbury	AUT	0.1	0.52	0.16	0.27
Raymond Chandler	AUT	0.18	0.51	0.2	0.39
Roald Dahl	AUT	0.83	0.2	0.07	0.23
Roberto Bolaño	AUT	0.08	0.51	0.14	0.23
Ruth Rendell	AUT	0.21	0.64	0.26	0.53
Saskia De Coster	AUT	0.26	0.42	0.19	0.41
Simone van der Vlugt	AUT	0.23	0.49	0.2	0.48
Stefan Zweig	AUT	0.13	0.62	0.21	0.35
Stephen King	AUT	0.79	0.23	0.09	0.27
Stieg Larsson	AUT	0.51	0.47	0.24	0.71
Sue Grafton	AUT	0.12	0.55	0.17	0.32
Susan Smit	AUT	0.33	0.42	0.2	0.48
TC Boyle	AUT	0.29	0.6	0.27	0.61
Thea Beckman	AUT	0.56	0.36	0.18	0.53
Tim Krabbé	AUT	0.37	0.33	0.16	0.39
Tom Clancy	AUT	0.25	0.45	0.19	0.44
Tom Lanoye	AUT	0.81	0.24	0.09	0.27
Toni Morrison	AUT	0.18	0.45	0.17	0.38

Umberto Eco	AUT	0.32	0.42	0.2	0.56
Uwe Tellkamp	AUT	0.13	0.41	0.14	0.26
Virginia Woolf	AUT	0.32	0.42	0.2	0.5
Vladimir Nabokov	AUT	0.29	0.55	0.25	0.55
Willem Frederik Hermans	AUT	0.27	0.46	0.2	0.41
Andrée Oudin	NON	0.14	0.62	0.21	0.36
Chiara Ricci	NON	0.16	0.44	0.16	0.33
E Buxton	NON	0.08	0.3	0.08	0.17
EL Wilford	NON	0.16	0.51	0.19	0.3
Elizabeth Wigelsworth	NON	0.14	0.45	0.15	0.29
Emily Oldani	NON	0.08	0.58	0.16	0.24
Emmanuelle Duvernay	NON	0.08	0.56	0.15	0.23
Eric Ferey	NON	0.1	0.56	0.17	0.27
Georges Roudaut	NON	0.09	0.5	0.14	0.24
Hans Ulfsson	NON	0.11	0.53	0.16	0.24
Hendrik van Weenen	NON	0.11	0.58	0.18	0.3
HM van der Grinten	NON	0.15	0.53	0.19	0.35
Jane Jessup	NON	0.06	0.37	0.09	0.17
John Kestley	NON	0.13	0.49	0.16	0.3
John Punnett	NON	0.13	0.51	0.17	0.32
Jorge Eudoro Remache	NON	0.04	0.24	0.05	0.12
Judith L Schecter	NON	0.11	0.57	0.18	0.26
Kathryn Lightner	NON	0.1	0.52	0.15	0.26
Kelly Weaver	NON	0.1	0.53	0.16	0.24
Kettil Christoffersen	NON	0.09	0.48	0.14	0.26
Kim Wassing	NON	0.03	0.24	0.04	0.06
Konstantin Ryschkov	NON	0.14	0.52	0.18	0.33
Kyra Appels	NON	0.08	0.45	0.12	0.23
Ludwig Lorenz	NON	0.13	0.46	0.15	0.27
Mahmoud Abdellah	NON	0.21	0.45	0.18	0.36
Marcus Fernandes	NON	0.14	0.48	0.16	0.33
Mark Robin	NON	0.11	0.55	0.17	0.27
Martijn van der Worp	NON	0.12	0.59	0.19	0.32
Mathijs L van Bueren	NON	0.1	0.35	0.1	0.23
Melanie Marrero Morales	NON	0.13	0.51	0.17	0.33
Pablo Daniel Gonzalez	NON	0.15	0.45	0.16	0.33
Pim Duijster	NON	0.1	0.51	0.15	0.23
Richard Grigley	NON	0.1	0.49	0.15	0.24
Robert Teesdale	NON	0.09	0.37	0.1	0.2
Roberto Borsani	NON	0.12	0.51	0.16	0.29
Roy Leeman	NON	0.14	0.44	0.15	0.26
Sara Lakin	NON	0.1	0.5	0.15	0.27
Theresa Ziegler	NON	0.18	0.44	0.17	0.3
Tim Singler	NON	0.13	0.65	0.22	0.36

Tomas Arensman	NON	0.05	0.37	0.08	0.12
Yasushi Sugawara	NON	0.1	0.6	0.18	0.29
Zofia Kwiatkowski	NON	0.1	0.49	0.15	0.23

Note: Item.Tot.woi = correlation of item with total test score; Item.Rel.woi = item reliability index; Discrim = discrimination.

5. Idiom recognition test

1. Tussen de regels door lezen (de diepere betekenis opmerken)
(EN: read between the lines)

lezen terwijl je iets iets anders doet	iets oppervlakkig lezen
iets niet begrijpen	de diepere betekenis opmerken

2. . Iemand iets in de maag splitsen (iemand iets dwingen te doen)
(EN: force someone to do something)

iemand iets te eten geven	iemand iets dwingen te doen
iemand ergens de schuld van geven	iemand met iets helpen

3. Een slag om de arm houden (iets onder voorbehoud afspreken)
(EN: conditionally agree on something)

iets onder voorbehoud afspreken	iets voor iemand willen doen
iets zonder afspraak uitvoeren	iets zonder beloning doen

4. Een gat in de lucht springen (heel blij zijn)
(EN: to be very happy)

gevaar lopen	voorzichtig zijn
heel blij zijn	bezorgd zijn

5. Iets uit je duim zuigen (iets verzinnen)
(EN: to make something up)

iets verzinnen	iets vergeten
iets verstoppen	iets heel goed kennen

6. Van zijn stokje gaan (flauwvallen)
(EN: to faint)

verhuizen	naar bed gaan
flauwvallen	lef hebben

7. Het schip ingaan (verliezen)
(EN: to lose)

trouwen	verliezen
zich verstoppen	gek worden

8. Iemand in de kaart spelen (iemand onbedoeld helpen)
(EN: to help someone unintentionally)

iemand onbedoeld helpen	iemand onbedoeld tegenwerken
met iemand samenwerken	met iemand ruzie hebben

9. Iets onder de knie hebben (iets goed kunnen)
(EN: to be good at something)

op iets kunnen zitten	iets vergeten zijn
iets niet willen doen	iets goed kunnen

10. Tegen de lamp lopen (betrapt worden)
(EN: to get caught)

snel beledigd zijn	betrapt worden
ergens mee stoppen	geslagen worden

6. Prescriptive grammar test

(green = Correct, red = incorrect)

Sentence	Correctness	Correct word	Category
De koekjes zijn even lekker **als** de taart van gisteren<br>(The cookies taste just as good as the cake yesterday)	correct	als	als/dan
Mijn broer is een jongen **die** altijd veel vriendinnen heeft<br>(My brother is a guy who always has a lot of girlfriends)	correct	die	die/dat
Er werd breed **geglimlacht** op de familiefoto<br>(There were broad smiles on the family photo)	correct	geglimlacht	spelling
Jij bent veel beter dan **mij** in het bedenken van oplossingen<br>(You are much better than me in coming up with solutions)	incorrect	ik	mij/ik
Misschien willen **hun** even alleen zijn<br>(Maybe they just want to be alone)	incorrect	zij	hun/zij
Daan is even geschikt **als** Sanne voor die functie<br>(Daan is as suitable as Sanne for that position)	correct	als	als/dan
Ik heb twee keer zo veel boeken **als** mijn broer<br>(I have twice as many books as my brother)	correct	als	als/dan
Suriname is vier keer zo groot **als** Nederland<br>(Suriname is four times the size of the Netherlands)	correct	als	als/dan
Waarom werkt Jan even hard **als** Gert aan de opdracht?<br>(Why does Jan work as hard as Gert on the assignment?)	correct	als	als/dan
De jongen eet veel minder **als** zijn grote neef<br>(The boy eats much less than his big cousin)	incorrect	dan	als/dan
Een dokter verdient meer **als** een ervaren timmerman<br>(A doctor earns more than an experienced carpenter)	incorrect	dan	als/dan
Niets is mooier **als** een zonsondergang op het strand<br>(Nothing is more beautiful than a sunset on the beach)	incorrect	dan	als/dan
Waarom werken zij sneller **als** de andere ploeg?<br>(Why do they work faster than the other team?)	incorrect	dan	als/dan
Ik praat met een vrouw **die** een rode jurk draagt<br>(I talk to a woman who is wearing a red dress)	correct	die	die/dat
In haar kamer staat een foto **die** ik nog nooit gezien heb<br>(There is a picture in her room that I have never seen before)	correct	die	die/dat
Piet werkt in een kledingwinkel **die** ik te duur vind<br>(Piet works in a clothing store that is too expensive I think)	correct	die	die/dat
Wij wachten op een vriend **die** altijd laat is	correct	die	die/dat

(We are waiting for a friend who is always late)

Daarachter ligt een gebied **die** de boer heeft opgekocht<br>(Behind it is an area that the farmer bought up)	incorrect	dat	die/dat
Hij was niet blij met een filmpje **die** van hem was gemaakt<br>(He was not happy with the video that was about him)	incorrect	dat	die/dat
Kim is in een vliegtuig gestapt **die** naar Londen gaat<br>(Kim boarded a plane that is going to London)	incorrect	dat	die/dat
Vorige week heb ik een boek gelezen **die** net is uitgebracht<br>(Last week I read a book that has just been released)	incorrect	dat	die/dat
Gisteravond heb ik **hun** gelukkig nieuwjaar gewenst<br>(Last night I wished them happy new year)	correct	hun	hun/zij
Gisteren heb ik **hun** twee boeken gegeven<br>(Yesterday I gave them two books)	correct	hun	hun/zij
Misschien zitten die kleren **hun** niet lekker<br>(Maybe those clothes are not so comfortable to them)	correct	hun	hun/zij
Morgen zullen we **hun** adviseren om de trein te nemen<br>(Tomorrow we will advise them to take the train)	correct	hun	hun/zij
Hopelijk gaan **hun** niet meer verhuizen<br>(Hopefully they will not move again)	incorrect	zij	hun/zij
Samen hebben **hun** heel veel plezier<br>(Together they have a lot of fun)	incorrect	zij	hun/zij
Vorige week liepen **hun** naar de speeltuin<br>(Last week they walked to the playground)	incorrect	zij	hun/zij
Waarom komen **hun** nu niet naar het feestje?<br>(Why are they not coming to the party?)	incorrect	zij	hun/zij
Hij kan Vera beter begrijpen dan **mij** omdat ze langzamer spreekt<br>(He can understand Vera better than he understands me, because she speaks slower)	correct	mij	mij/ik
Hij vindt Linda aardiger dan **mij** maar niet grappiger<br>(He likes Linda better than me but he doesn't think she is funnier)	correct	mij	mij/ik
Jullie hielpen Dirk net zo veel als **mij** met het huiswerk<br>(You helped Dirk as much as you helped me with homework)	correct	mij	mij/ik
Kees belt Lotte vaker dan **mij** als hij iets nodig heeft<br>(Kees is calling Lotte more than he is calling me if he needs something)	correct	mij	mij/ik
Jij kunt hoger dan **mij** klimmen in deze boom<br>(You can climb higher in this tree than me)	incorrect	ik	mij/ik
Mijn collega gaat een week langer dan **mij** weg in de vakantie	incorrect	ik	mij/ik

(My colleague is one week longer on holiday than I am)			
Mijn zus is niet zo handig als **mij** in dat soort dingen<br>(My sister is not as handy with that kind of things as I am)	incorrect	ik	mij/ik
Steven heeft eerder dan **mij** zijn rijbewijs gehaald<br>(Steven got his driver’s license before I did)	incorrect	ik	mij/ik
Het kindje had koorts en **stuiptrekte** met zijn armen en benen<br>(The child had a fever and convulsed with his arms and legs)	correct	stuiptrekte	spelling
In het café zat een man die tegen betaling **waarzegde**<br>(There was a man in the cafe who told fortunes for a fee)	correct	waarzegde	spelling
Mijn vader heeft jaren **gezweefvliegd** bij een Friese club<br>(My father has been gliding for years at a Frisian club)	correct	gezweefvliegd	spelling
Thuis werd er voortdurend **gebekvecht** om de tablet<br>(At home there was constantly wrangling for the tablet)	correct	gebekvecht	spelling
De schoonmaakster **stofzoog** het hele huis grondig<br>(The cleaning woman vacuumed the entire house thoroughly)	incorrect	stofzuigde	spelling
Het is bekend dat een arts in de middeleeuwen patiënten **aderlaatte**<br>(It is known that a doctor in the Middle Ages bled patients)	incorrect	aderliet	spelling
Ik bewonderde de figuren die uit marmer waren **beeldgehouwen**<br>(I admired the figures sculpted from marble)	incorrect	gebeeldhouwd	spelling
Tessa had **schatergelachen** om de opmerking van haar vriendin<br>(Tessa had laughed at her friend’s comment)	incorrect	geschaterlacht	spelling

7. Syntest

Available upon request.

General cognitive skills

8. Auditory simple reaction time test

Auditory stimulus: sine tone (550 Hz)

9. Auditory choice reaction time test

Auditory stimulus: low (300 Hz) or high sine tone (800 Hz)

10. Letter comparison test

Block	Condition	Top	Bottom
Practice	same	LVK	LVK
Practice	same	RWP	RWP
Practice	same	GTK	GTK
Practice	different	RDM	RNM
Practice	different	HSW	HSF
Practice	different	BKT	BKJ
1	same	VMJ	VMJ
1	same	TDL	TDL
1	same	MWP	MWP
1	same	TDM	TDM
1	same	RNH	RNH
1	same	FZT	FZT
1	same	GTK	GTK
1	same	KNV	KNV
1	same	RWP	RWP
1	same	BWS	BWS
1	same	LVK	LVK
1	same	RJN	RJN
1	different	TZF	TCF
1	different	DPG	DWG
1	different	RDM	RNM
1	different	KTG	KTB
1	different	HSW	HSF
1	different	ZKD	NKD
1	different	FRW	CRW
1	different	DWJ	DHJ
1	different	BKT	BKJ
1	different	WDN	SDN

1	different	THP	DHP
1	different	PBS	PBD
2	same	BWSDNJ	BWSDNJ
2	same	RJNTHP	RJNTHP
2	same	GTKPBS	GTKPBS
2	same	RWTDPG	RWTDPG
2	same	MWPHSW	MWPHSW
2	same	LVKWDN	LVKWDN
2	same	FZTBKL	FZTBKL
2	same	RNHKTG	RNHKTG
2	same	TDLKZF	TDLKZF
2	same	KNVRDM	KNVRDM
2	same	TDMFRW	TDMFRW
2	same	VMJZKD	VMJZKD
2	different	BKJFZT	BKVFZT
2	different	TCFBDL	TMFBDL
2	different	KTBRNH	KTFRNH
2	different	SPNTDM	SPNTDG
2	different	CRWVMJ	CRWVLJ
2	different	HSFMWP	HSFJWP
2	different	DWGRVP	KWGRVP
2	different	DHPRJN	DHPRJL
2	different	RNMKSV	GNMKSV
2	different	DHJBWS	DTJBWS
2	different	PBDGTK	PBDLTK
2	different	NKDLVK	NKDLPK

Note: Top = letters at top of the screen; Bottom = letters at the bottom of the screen.

11. Visual simple reaction time test

Visual stimulus:

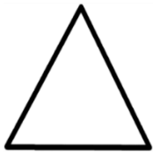

12. Visual choice reaction time test

Visual stimuli:

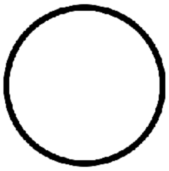

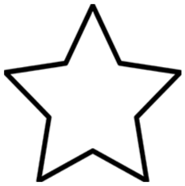

13. Digit span test (forward & backward)

Digit span test forward

**Trial length**		
2 digits	1-7	6-3
3 digits	5-8-2	6-9-4
4 digits	6-4-3-9	7-2-8-6
5 digits	4-2-7-3-1	7-5-8-3-6
6 digits	3-9-2-4-8-7	6-1-9-4-7-3
7 digits	4-1-7-9-3-8-6	5-9-1-7-4-2-8
8 digits	3-8-2-9-5-1-7-4	5-8-1-9-2-6-4-7
9 digits	2-7-5-8-6-2-5-8-4	7-1-3-9-4-2-5-6-8

Digit span test backward

**Trial length**		
2 digits	2-4	5-7
3 digits	4-1-5	6-2-9
4 digits	3-2-7-9	4-9-6-8
5 digits	1-5-2-8-6	6-1-8-4-3
6 digits	5-3-9-4-1-8	7-2-4-8-5-6
7 digits	4-7-3-9-1-2-8	8-1-2-9-3-6-5
8 digits	7-2-8-1-9-6-5-3	9-4-3-7-6-2-5-8

14. Corsi block clicking test (forward & backward)

Example trial (3 blocks):

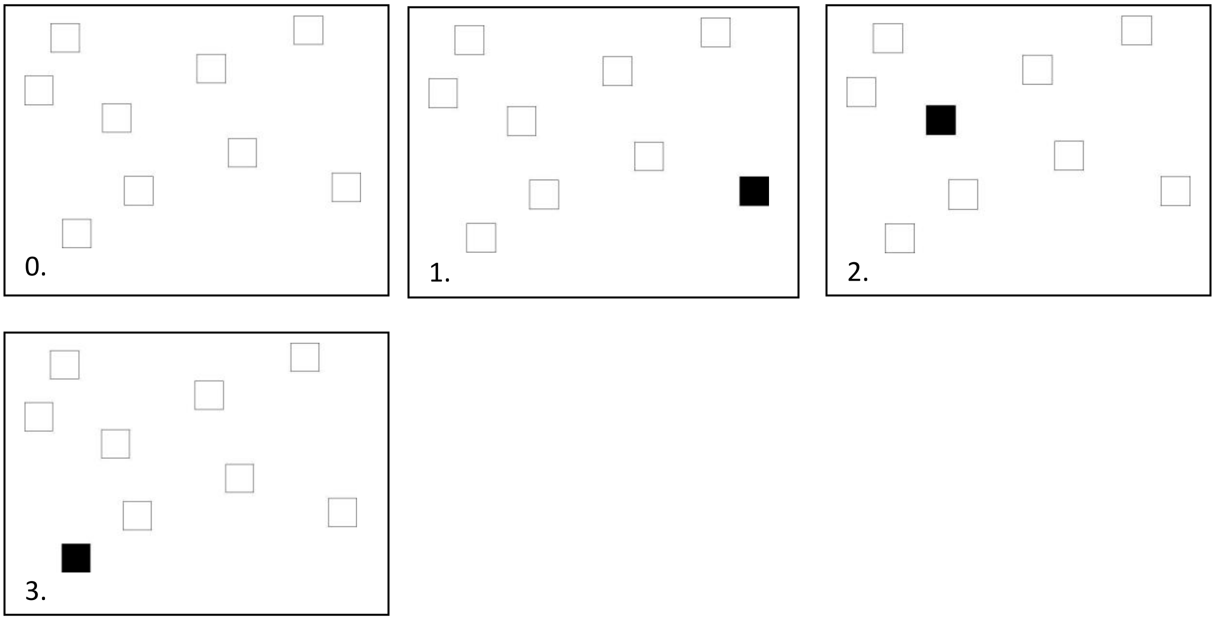

15. Eriksen flanker test

**Condition**	**Stimulus**
neutral	--<--
congruent	<<<<<
incongruent	>><>>

16. Antisaccade test

Fixation cross – Cue – Target - Mask

17. Raven's advanced progressive matrices test

Example trial:

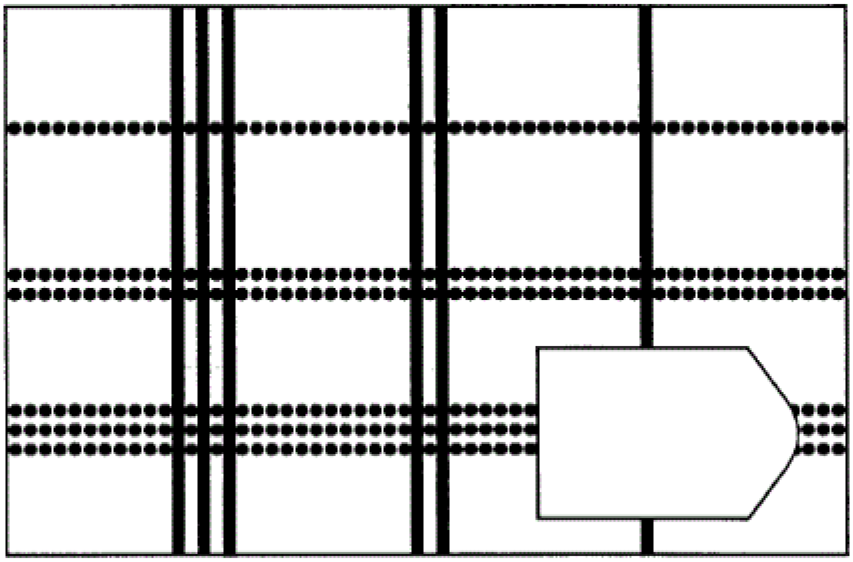

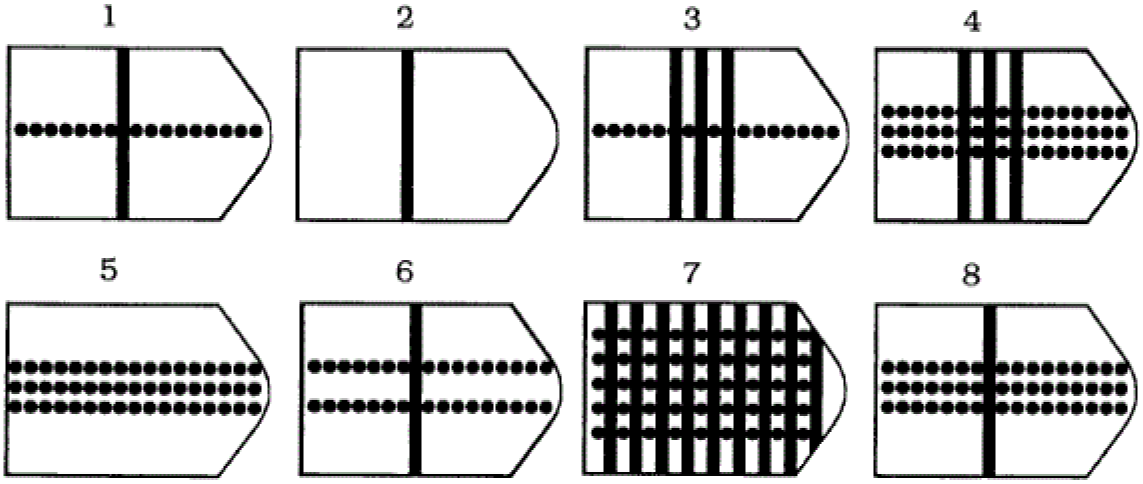

Linguistic processing skills: Word production

18. Picture naming test

Target	Zipf Frequency	Prevalence
kussen (pillow, practice)	4.66	1.00
lippenstift (lipstick, practice)	3.81	1.00
schaats (skate, practice)	2.81	1.00
zebra (zebra, practice)	3.49	1.00
appel (apple)	4.01	1.00
bureau (bureau)	4.83	0.99
cactus (cactus)	3.26	0.99
deur (door)	5.39	1.00
egel (hedgehog)	2.76	1.00
eiland (island)	4.71	1.00
emmer (bucket)	3.83	1.00
gieter (watering can)	2.51	0.99
glijbaan (slide)	2.7	1.00
helm (helmet)	4.04	1.00
jurk (dress)	4.75	1.00
koffer (suitcase)	4.53	1.00
konijn (rabbit)	4.28	1.00
kraan (tap)	3.81	1.00
neus (nose)	4.85	1.00
orka (orca)	2.53	0.99
paspoort (passport)	4.27	1.00
passer (compasses)	2.04	0.99
pincet (tweezers)	2.95	0.99
pinguin (penguin)	3.39	0.98
punaise (pushpin)	2.26	0.99
raam (window)	4.85	1.00
schaap (sheep)	3.82	1.00
schoen (shoe)	4.13	0.99
schommel (swing)	3.2	0.99
sigaret (cigarette)	4.44	1.00
slang (snake)	4.33	1.00
sleutel (key)	4.91	1.00
snavel (beak)	3.24	1.00
tafel (table)	4.92	1.00
telefoon (telephone)	5.2	1.00
trommel (drum)	3.27	1.00
varken (pig)	4.39	1.00
vergiet (colander)	2.94	1.00
vleugel (wing)	3.95	0.99
vogel (bird)	4.51	1.00
vuist (fist)	3.83	1.00

waaier (fan)	3.01	0.99
wortel (carrot)	3.79	1.00
zeef (sieve)	2.76	1.00

19. Rapid automatized naming

Word	Condition	ZipF	PND	Prevalence
bed (bed)	high frequency - high PND	5.38	38	1.00
hoed (hat)	high frequency - high PND	4.56	31	1.00
mond (mouth)	high frequency - high PND	5.22	20	1.00
paard (horse)	high frequency - high PND	4.92	18	1.00
tent (tent)	high frequency - high PND	4.61	20	1.00
arm (arm)	high frequency - low PND	4.9	7	1.00
bank (couch)	high frequency - low PND	4.96	12	1.00
deur (door)	high frequency - low PND	5.39	12	1.00
glas (glass)	high frequency - low PND	4.76	7	1.00
trap (stairs)	high frequency - low PND	4.72	10	1.00
bijl (axe)	low frequency - high PND	3.97	17	1.00
boon (bean)	low frequency - high PND	3.15	19	0.99
mand (basket)	low frequency - high PND	3.63	25	1.00
pauw (peacock)	low frequency - high PND	2.98	25	1.00
vaas (vase)	low frequency - high PND	3.66	26	1.00
dolk (dagger)	low frequency - low PND	3.65	8	1.00
hark (rake)	low frequency - low PND	3.18	9	0.99
mais (corn)	low frequency - low PND	2.88	5	0.96
slee (sled)	low frequency - low PND	3.61	7	1.00
wieg (cradle)	low frequency - low PND	3.74	11	1.00

Note: ZipF = Zipf frequency; PND = phonological neighborhood density.

20. Antonym production

Cue	ZipF	Prevalence	Target	ZipF	Prevalence	Alternative(s)
dwerg (dwarf, practice)	3.95	1.00	reus (giant)	3.87	1.00	
vallen (fall, practice)	5.26	1.00	opstaan (rise)	4.62	1.00	
winst (gain, practice)	4.34	0.99	verlies (loss)	4.69	1.00	
absent (absent)	2.43	0.98	present (present)	3.29	0.99	aanwezig (present)
bevestiging (confirmation)	3.8	1.00	ontkenning (denial)	3.47	1.00	
deficientie (deficieny)	-	0.87	overdaad (excess)	2.7	1.00	overschot, teveel (surplus, too much)
fluisteren (whisper)	3.65	1.00	schreeuwen (scream)	4.47	1.00	gillen, roepen (shout, yell)
geforceerd (forced)	3.27	1.00	vrijwillig (voluntary)	3.98	1.00	dwangloos, natuurlijk (noncompulsory, naturally)
goedkoop (cheap)	4.21	1.00	duur (expensive)	4.54	1.00	
lawaai (noise)	4.23	1.00	stilte (silence)	4.58	1.00	rust, rustig (quiet)
legaal (legal)	4.07	1.00	illegaal (illegal)	4.24	0.99	verboden (forbidden)
leugen (lie)	4.42	0.99	waarheid (truth)	5.28	1.00	
mager (skinny)	3.94	1.00	dik (fat)	4.63	1.00	gezet (heavy)
minimaal (minimal)	3.69	1.00	maximaal (maximal)	3.73	1.00	
monochroom (monochrome)	-	0.91	bont (coloured)	3.63	1.00	polychroom, kleurrijk (polychrome, colourful)
nadeel (disadvantage)	3.54	1.00	voordeel (advantage)	4.32	1.00	
negatief (negative)	4.16	1.00	positief (positive)	4.17	1.00	
officieus (unofficially)	2.95	0.97	officieel (official)	4.44	0.99	
ouderwets (old fashioned)	3.98	0.99	modern (modern)	3.81	0.99	eigentijds, hedendaags, geavanceerd (contemporary, advanced)
overwinning (victory)	4.36	1.00	nederlaag (defeat)	3.62	1.00	verlies (loss)
passief (passive)	3.06	1.00	actief (active)	3.99	1.00	
succes (success)	5.02	1.00	mislukking (failure)	3.71	0.99	sof, flop, falen (fiasco, fail)
tanen (decrease)	1.7	0.87	toenemen (increase)	3.14	1.00	stijgen (rise)
theoretisch (theoretical)	3.48	1.00	praktisch (practical)	4.06	1.00	
traag (slow)	3.99	1.00	snel (fast)	5.67	1.00	gauw, vlug (soon, quickly)
unanimiteit (unanimity)	-	0.85	Onenigheid (disagreement)	3.45	1.00	verdeeldheid (dividedness)
vijand (enemy)	4.78	0.99	vriend (friend)	5.69	1.00	bondgenoot (ally)
zaaien (sow)	3.6	0.99	oogsten (harvest)	3.64	1.00	

Note: ZipF = Zipf frequency.

21. Verbal fluency

Categories

1) Animals

2) Food and drinks

Letters

1) Letter M

2) Letter S

22. Maximal speech rate

Task: Recite the months of the year as quickly as possible with clear pronunciation.

23. One-minute-test

waar	zijpad	priemen	struikgewas
kar	inham	getik	speelvergunning
been	stoutheid	oertijd	hernieuwen
min	proefstuk	aanplanten	berging
vos	lapje	slopen	nanacht
net	doch	vooruitduwen	uitspuwen
bruin	vegen	steigeren	herplaatsing
hand	koplamp	opsparen	onnozel
morgen	koelte	handelaar	medelid
eten	rekenen	diamant	zijrivier
mak	verdieping	bezig	vermindering
voorbij	geknoei	hanger	reling
hamer	genieten	puree	getuigschrift
zieke	paffen	achting	herkenning
luilak	warenhuis	opspuiten	overloodsen
trekken	aanzitten	plaatsnemen	bijeenkomst
verlaten	stelen	navliegen	tekortkoming
verhuizen	treurig	messteek	gelaatskleur
worden	voorstaan	ontschieten	beoefenaar
dichten	overlaten	scheepgaan	welsprekend
uithuilen	afwissen	boerenwoning	verslagenheid
kruid	groeve	begaafd	ontraadselen
grootmoeder	verdwijnen	pijlsnel	omslachtig
roeping	dichtwerpen	schouwing	tijdsbepaling
laan	hozen	schoenveter	ontworstelen
kruimel	zorgvol	kibbelen	saluutschot
heenlopen	spiegelen	schede	wetsrol
fietsbel	stamtafel	rotsvast	oneffen
schroeven	houtvlot	frommelen	rubberaanplanting

English translation:

where/true	side road	to pierce	shrubbery
cart	cove	ticking noise	event licence
leg	audacity	prehistory	to renew
wet nurse	masterpiece	to plant	storage room
fox	[little] patch	to demolish	late-night
net	however	to push forward	to spit out
brown	to sweep	to prance	reinsertion
hand	headlight	to save up	empty-headed
morning	coolness	merchant	fellow member
to eat	to calculate	diamond	tributary
tame	floor [of a building]	busy	reduction
past	mess	pendant	railing
hammer	to enjoy	purée	certificate
sick person	to smoke	regards	recognition
lazybones	warehouse	to spray	to pilot [a ship to a place]
to pull	to sit at	to take a seat	meeting
to leave	to steal	to fly after	shortcoming
to move out	sad	knife stab	complexion
to become	to stand for	to slip [a memory]	practitioner
to write poetry	to leave something	to embark	eloquent
to cry	to wipe off	farmhouse	dismay
herb	quarry	gifted	to unravel
grandmother	to disappear	swift as an arrow	cumbersome
calling	to close off	inspection	time determination
avenue	to bail water out	shoelace	to break away from
crumb	caring	to bicker	gun salute
to walk away	to mirror	sheath	law scroll
bicycle bell	table for regular guests	rock-solid	uneven
to screw	wooden raft	to fumble	rubber plantation

24. Klepel test

taaf	zapod	briekem	stroukgewep
vas	ijnhas	gelup	stoolvordanning
deek	stuikhaut	oeltijk	lerdieuwen
nim	truufstin	aafplenton	dergonk
gol	kepjo	drepes	nonochd
vek	roch	keenautluwer	outpluwin
fruim	veben	steiperan	derkraatsong
jund	fiplemp	ipsporel	annasel
nargel	goelte	hosdelaar	sederab
aven	rapones	fiamont	vijruzier
baf	serdieving	podig	dervinseming
noogsij	teploei	denger	talung
zaper	megoezen	butee	gemuikschruft
hiene	zaffon	ochtung	zertonning
duihas	worenpuis	okspuilon	iverleetsel
trinnen	oonlittek	klaanstemen	nijaaltorst
kerpanes	stebun	vodrienen	gepaltnomeng
retluiven	treukel	baffraas	bolaatsvloeg
lardon	voogstaag	intschuipen	geoeteraan
wochtel	ogerjaken	scheempaag	berstrapind
ounlouget	ofwannin	souwentonang	terstogenheis
vreud	groeza	beguisd	antpiedselen
kraatduiper	berdwijmel	wijlspok	onvlachtig
poebing	pochtbersen	schieveng	notsberapong
laas	wozen	schautkeder	ontmertselen
steumel	nargvop	mokkelin	taluukschet
hootgepen	spietelin	scheke	batskel
liertvek	kwilhogel	dotshast	inekken
schruidel	muigkrit	krobbelon	nalleroonplinteng

Linguistic processing skills: Word comprehension

25. Monitoring in noise in lists

green = target; orange = foil

Non-word monitoring in non-word lists

Round	SNR	Condition	Cue basis	Cue	Word1	Word2	Word3	Word4	Word5	Word6
Practice	-2	target-only	schaap (sheep)	schuip	kelf	schuip	bientjel	jirker		
Practice	-8	target-absent	zak (bag)	zas	pieder	krel	ern			
Practice	-12	foil-target	hart (heart)	hats	traap	halgherm	spaas	hats	baphies	
1	-2	target-only	touw (rope)	tomp	werrens	knaf	regni	apveid	tomp	penk
1	-4	target-absent	tang (pliers)	mang	halktat	wieel	vreeuw			
1	-6	target-only	kroon (crown)	broon	nijmsaard	wulen	pluif	broon	swi	
1	-8	foil-target	man (man)	mas	kieg	guitel	marralk	mas	res	
1	-10	target-absent	eend (duck)	oend	fraal	stoek	raf	noger		
1	-12	foil-target	taart (cake)	taapt	taafmong	taapt	sloog	gies		
1	-14	target-only	kaars (candle)	kiers	slon	kiers	waga			
1	-16	target-absent	gat (hole)	gar	famker	cigroon	snaan	vets	rirte	
1	-18	foil-target	bom (bomb)	bog	bofreun	bog	zamp			
1	-20	target-only	haai (shark)	hoei	viet	spunel	hoei	nielund		
2	-2	foil-target	band (tire)	baxt	wirums	hesk	bafruip	pras	baxt	grulon
2	-4	target-only	kruis (cross)	fluis	doon	rades	fluis	eedond	wemp	goeling
2	-6	foil-target	hand (hand)	haps	harfbijg	dreflier	worzel	haps	keeg	
2	-8	target-absent	zwaan (swan)	praan	veg	gebog	siekoed	fonguin		
2	-10	target-only	bloem (flower)	blijm	roor	anwoep	blijm	efschauk		
2	-12	target-only	smaak (taste)	smaaf	gog	smaaf	kleis			
2	-14	foil-target	hoed (hat)	hoem	hoegpaat	strieg	hoem	vleek		
2	-16	target-only	bier (beer)	biem	fots	olgeer	biem	tijnkel		
2	-18	target-absent	peen (carrot)	peef	zek	kijs	miel	draxtor	redel	vrop
2	-20	foil-target	bord (plate)	bops	bolgwap	bops	lank			

3	-2	target-absent	loep (look)	luip	baaf	mefdel	schuik			
3	-4	foil-target	broer (brother)	broen	launning	broefnak	pag	broen	akbuip	rornas
3	-6	target-absent	doek (cloth)	deek	oedbijp	jor	stunk	burbijn		
3	-8	target-only	muis (mouse)	muin	pran	muin	barp	euzer	schekder	
3	-10	foil-target	hond (dog)	hogt	aafbag	holmdrins	hogt	toor	goe	
3	-12	target-absent	beest (beast)	beept	luin	spom	zoeg	kraaf	roog	
3	-14	target-absent	blaas (bladder)	draas	antveg	wref	jomel	loor	proos	
3	-16	foil-target	brood (bread)	broog	dauk	broopkimp	broog	knekgel		
3	-18	target-only	pop (doll)	pos	woetaug	pos	snerm	goos		
3	-20	target-absent	klad (draft)	klag	vuitlil	laaps	hook	brelp	raben	otrong

Note: SNR = Signal-to-noise ratio.

Round	SNR	Condition	Cue	Word1	Word2	Word3	Word4	Word5	Word6
Practice	0	target-only	kers (cherry)	zebra (zebra)	kers (cherry)	zwembad (swimming pool)	stap (step)		
Practice	-6	target-absent	hert (deer)	speld (pin)	dijk (dike)	lid (member)			
Practice	-10	target-foil	zalf (ointment)	pomp (pump)	zandbak (sandbox)	hof (court)	zalf (ointment)	bosjes (bushes)	
1	0	target-only	fiets (bicycle)	ontbijt (breakfast)	bijbel (Bible)	strop (noose)	persoon (person)	fiets (bicycle)	voorbeeld (example)
1	-2	target-absent	veer (feather)	geld (money)	oorlog (war)	slaap (sleep)			
1	-4	target-only	spook (ghost)	ruil (exchange)	vlecht (braid)	klomp (clog)	spook (ghost)	applaus (applause)	
1	-6	target-foil	roos (rose)	bries (breeze)	veerman (ferryman)	roofdier (predator)	roos (rose)	klik (click)	
1	-8	target-absent	roem (fame)	tulband (turban)	kalf (calf)	object (object)	steel (stem)		
1	-10	target-foil	mouw (sleeve)	moutwijn (malt wine)	mouw (sleeve)	kamp (camp)	sigaar (cigar)		
1	-12	target-only	stift (marker)	zondag (Sunday)	stift (marker)	tijd (time)			
1	-14	target-absent	haas (hare)	pand (house/property)	koffie (coffee)	scherf (shard)	regel (rule)	armoe (poverty)	
1	-16	target-foil	bus (bus)	burger (burger)	bus (bus)	wieg (cradle)			
1	-18	target-only	bijl (axe)	schildpad (turtle)	robot (robot)	bijl (axe)	gitaar (guitar)		
2	0	target-foil	sik (goatee)	piloot (pilot)	hal (hall)	signaal (signal)	ets (etching)	sik (goatee)	fortuin (fortune)
2	-2	target-only	moord (murder)	fluit (flute)	begrip (understanding)	moord (murder)	aalmoes (alms)	veulen (foal)	sla (lettuce)
2	-4	target-foil	huis (house)	huidcel (skin cell)	vest (vest)	gebied (area)	huis (house)	kameel (camel)	
2	-6	target-absent	uil (owl)	folder (flyer)	pil (pill)	lijn (line)	ballon (balloon)		
2	-8	target-only	been (leg)	feestdag (holiday)	merk (brand)	been (leg)	water (water)		
2	-10	target-only	sjaal (scarf)	gewei (antlers)	sjaal (scarf)	proef (test)			

2	-12	target-foil	zon (sun)	zolder (attic)	moed (courage)	zon (sun)	bakker (baker)		
2	-14	target-only	rok (skirt)	schip (ship)	waanzin (madness)	rok (skirt)	hertog (duke)		
2	-16	target-absent	bloed (blood)	lijm (glue)	kegel (cone)	zoon (son)	plaats (place)	viool (violin)	jager (hunter)
2	-18	target-foil	jas (coat)	jargon (jargon)	jas (coat)	print (print)			
3	0	target-absent	zorg (care)	naam (name)	sloep (sloop)	tempel (temple)			
3	-2	target-foil	blaar (blister)	non (nun)	blaaspijp (blowpipe)	liter (liter)	blaar (blister)	tas (bag)	mis (mass)
3	-4	target-absent	steeg (alley)	hoofdpijn (head ache)	actie (action)	meter (meter)	gif (poison)		
3	-6	target-only	baard (beard)	voedsel (food)	baard (beard)	gil (scream)	kramp (cramp)	zadel (saddle)	
3	-8	target-foil	hoef (hoof)	maandag (Monday)	hoektand (canine)	hoef (hoof)	zaal (hall/room)	worp (throw)	
3	-10	target-absent	slak (snail)	figuur (figure)	halm (grass stem)	voorkeur (preference)	toets (test)	kan (can)	
3	-12	target-absent	kom (bowl)	buil (bump)	piraat (pirate)	aanpak (approach)	nicht (cousin/niece)	film (film)	
3	-14	target-foil	oog (eye)	spoor (trail)	oostgrens (eastern border)	oog (eye)	versie (version)		
3	-16	target-only	spin (spider)	boog (bow)	spin (spider)	pleister (band aid)	tomaat (tomato)		
3	-18	target-absent	sop (suds)	kamer (room)	bles (blaze)	trots (pride)	ven (fen)	leraar (teacher)	deksel (lid)

Note: SNR = Signal-to-noise ratio.

Meaning monitoring in word lists

Round	SNR	Condition	Cue	Target	Word1	Word2	Word3	Word4	Word5	Word6
Practice	-2	target-present	wol (wool)	schaap (sheep)	keus (choice)	schaap (sheep)	borstel (brush)	cirkel (circle)		
Practice	-8	target-absent	tas (bag)	zak (bag)	gieter (watering can)	stel (couple)	erf (property)			
Practice	-12	target-present	long (lung)	hart (heart)	troep (mess)	kuiken (chick)	spies (skewer)	hart (heart)	badhuis (bathhouse)	
1	-2	target-present	knoop (button)	touw (rope)	oordeel (judgement)	staf (staff)	rente (interest)	arbeid (work)	touw (rope)	wenk (hint)
1	-4	target-absent	klem (clip)	tang (pliers)	hangmat (hammock)	riool (sewer)	sneeuw (snow)			
1	-6	target-present	prins (prince)	kroon (crown)	nijlpaard (hippo)	regen (rain)	druif (grape)	kroon (crown)	ski (ski)	
1	-8	target-present	vrouw (woman)	man (man)	kier (crack)	beitel (chisel)	datum (date)	man (man)	ren (run)	
1	-10	target-absent	dons (fluff)	eend (duck)	kwaal (disease)	sjeik (sheikh)	ras (breed)	zomer (summer)		
1	-12	target-present	vlaai (flan)	taart (cake)	wapen (weapon)	taart (cake)	sloot (ditch)	nies (sneeze)		
1	-14	target-present	vlam (flame)	kaars (candle)	slot (lock)	kaars (candle)	lama (lama)			
1	-16	target-absent	kuil (pit)	gat (hole)	masker (mask)	citroen (lemon)	traan (tear)	vent (guy)	hitte (heat)	
1	-18	target-present	knal (bang)	bom (bomb)	tiener (teenager)	bom (bomb)	zand (sand)			
1	-20	target-present	vin (fin)	haai (shark)	riet (cane)	puzzel (puzzle)	haai (shark)	weiland (meadow)		
2	-2	target-present	velg (rim)	Band (tire)	minuut (minute)	heup (hip)	eiwit (protein)	gras (grass)	band (tire)	sultan (sultan)
2	-4	target-present	graf (grave)	kruis (cross)	loon (salary)	dadel (date)	kruis (cross)	eiland (island)	werk (work)	voeding (food)
2	-6	target-present	duim (thumb)	hand (hand)	gedicht (poem)	premier (prime minister)	wortel (carrot)	hand (hand)	keer (occasion)	
2	-8	target-absent	nest	zwaan	vel	gebak	sieraad	fontein		

			(nest)	(swan)	(skin)	(pastry)	(ornament)	(fountain)		
2	-10	target-present	geur (smell)	bloem (flower)	koor (choir)	oproep (call)	bloem (flower)	afspraak (appointment)		
2	-12	target-present	tong (tongue)	smaak (taste)	god (god)	smaak (taste)	kluis (locker)			
2	-14	target-present	pet (cap)	hoed (hat)	cijfer (grade)	striem (stripe)	hoed (hat)	bleek (bleach)		
2	-16	target-present	tap (tap)	bier (beer)	fort (fort)	onweer (thunderstorm)	bier (beer)	vaarwel (farewell)		
2	-18	target-absent	biet (beet)	peen (carrot)	hek (fence)	wijs (tune)	ziel (soul)	tractor (tractor)	ketel (kettle)	stop (stop)
2	-20	target-present	krijt (chalk)	bord (plate)	najaar (autumn)	bord (plate)	land (land)			
3	-2	target-absent	glas (glass)	loep (lens)	baan (job)	middel (waist)	struik (bush)			
3	-4	target-present	zus (sister)	broer (brother)	leerling (student)	raket (rocket)	pad (toad)	broer (brother)	afloop (ending)	harnas (armor)
3	-6	target-absent	zeil (sail)	doek (cloth)	eerbied (respect)	por (poke)	stank (smell)	buslijn (bus line)		
3	-8	target-present	kaas (cheese)	muis (mouse)	plan (plan)	muis (mouse)	berg (mountain)	emmer (bucket)	schouder (shoulder)	
3	-10	target-present	kat (cat)	hond (dog)	aanbod (offer)	bamboe (bamboo)	hond (dog)	tooi (attire)	roe (birch rod)	
3	-12	target-absent	wild (game)	beest (beast)	luik (hatch)	slof (slipper)	voeg (joint)	kraag (collar)	room (cream)	
3	-14	target-absent	gal (bile)	blaas (bladder)	ontzag (awe)	chef (chef)	kogel (bullet)	leer (leather)	prijs (price)	
3	-16	target-present	korst (crust)	brood (bread)	duik (dive)	accent (accent)	brood (bread)	vleugel (wing)		
3	-18	target-present	kind (child)	pop (doll)	rijtuig (carrier)	pop (doll)	zwerm (swarm)	goot (gutter)		
3	-20	target-absent	schets (sketch)	klad (draft)	voetbal (football)	laars (boot)	rook (smoke)	breuk (fracture)	haven (port)	omgang (interaction)

Note: SNR = Signal-to-noise ratio.

26. Rhyme judgment

Condition	Non-word 1	Non-word 2
rhyme (practice)	kruul	bruul
rhyme (practice)	ries	zies
foil (practice)	zor	lon
unrelated (practice)	daaf	puis
rhyme	bienk	tienk
rhyme	doer	zoer
rhyme	dral	vral
rhyme	graak	plaak
rhyme	guust	muust
rhyme	haap	baap
rhyme	hur	nur
rhyme	jik	zik
rhyme	kel	mel
rhyme	klet	gret
rhyme	lous	gous
rhyme	meus	deus
rhyme	momp	tomp
rhyme	muik	huik
rhyme	narst	larst
rhyme	nelg	helg
rhyme	noost	woost
rhyme	peist	jeist
rhyme	sluin	pruin
rhyme	stas	pras
rhyme	staum	graum
rhyme	trop	glop
rhyme	veef	keef
rhyme	wes	nes
foil	bruip	fluik
foil	floes	broel
foil	gaam	jaal
foil	kag	taf
foil	krup	slun
foil	pum	wul
foil	spak	traf
foil	zeel	reem
unrelated	beus	fuug
unrelated	fap	lim
unrelated	kweig	preet
unrelated	meif	zoot
unrelated	mirf	julp
unrelated	stig	pruk

unrelated	treis	breuf
unrelated	wulg	zank

27. Auditory lexical decision

Word	ZipF	Prevalence	Non-word
bek (beak, practice)	4.71	1.00	bun (practice)<br>vor (practice)
aardbei (strawberry)	3.19	1.00	aardloe
ambtenaar (civil servant)	3.58	1.00	anktekier
arend (eagle)	3.31	1.00	olend
auto (car)	5.66	1.00	auli
azijn (vinegar)	3.23	1.00	ameen
bijbel (bible)	4.36	1.00	boevel
bliksem (lightning)	4.03	0.99	droksem
bloem (flower)	4.13	1.00	zwoem
brein (brain)	4.22	1.00	brien
bril (glasses)	4.39	1.00	vril
chocolade (chocolate)	4.14	1.00	quosirade
clown (clown)	4.07	1.00	clonx
doorn (thorn)	3.30	1.00	daarn
fles (bottle)	4.66	1.00	smes
gebouw (building)	4.83	1.00	gebamp
geraamte (skeleton	2.85	1.00	gedijgte
geschenk (gift)	4.27	1.00	geschork
gesp (buckle)	2.98	0.99	gelg
gitaar (guitar)	4.06	1.00	bitier
gong (gong)	2.68	0.97	gork
gras (grass)	4.27	1.00	gral
hagel (hail)	3.13	1.00	hepel
hark (rake)	3.18	0.99	halg
haver (oat)	2.95	1.00	faver
huig (uvula)	2.04	0.99	kuig
jager (hunter)	4.05	1.00	reber
kastanje (chestnut)	2.48	0.99	kollanje
kasteel (castle)	4.44	1.00	mastiel
kegel (pin)	2.63	0.99	mavel
kever (beetle)	3.34	1.00	mazer
klarinet (clarinet)	3.01	0.99	gralinek
klomp (clog)	2.95	1.00	gromp
korf (basket)	2.63	1.00	kolm
krater (crater)	3.33	1.00	grader
kruis (cross)	4.32	1.00	kries
lamp (lamp)	4.14	0.99	lard
magneet (magnet)	3.20	1.00	madveet
marmot (marmot)	2.63	0.99	marbit

meeuw (gull)	2.81	1.00	peeuw
microfoon (microphone)	4.01	1.00	miklohien
pantoffel (slipper)	2.36	1.00	pantabbel
papier (paper)	4.49	1.00	panoer
parfum (perfume)	4.04	0.99	palmus
pedaal (pedal)	2.95	1.00	periel
pistool (pistol)	5.01	1.00	postiel
pizza (pizza)	4.39	1.00	pebza
professor (professor)	4.83	0.97	rapek
raket (rocket)	4.17	1.00	ropaan
rozijn (raisin)	2.48	0.99	schebes
schemer (dusk)	2.57	0.99	schelg
scherm (screen)	4.14	1.00	schars
schors (bark)	3.06	1.00	sardaat
soldaat (soldier)	4.72	1.00	terekapie
televisie (television)	4.35	1.00	treik
trein (train)	4.86	0.99	letel
veter (shoe lace)	3.12	0.99	dijg
vijg (fig)	2.36	0.99	smaai
vlaai (flan)	2.26	1.00	vlijs
vlees (meat)	4.79	1.00	vliemkuig
voetbal (football)	4.11	0.99	vaatlal

Note: ZipF = Zipf frequency.

28. Semantic categorization

Category: Professions

Words	ZipF	Prevalence	Foils	ZipF	Prevalence
leraar (teacher, practice)	4.47	0.99	kofferbak (trunk, practice)	4.05	1.00
tandarts (dentist, practice)	4.16	1.00	woestijn (desert, practice)	4.44	1.00
architect (architect)	3.76	0.99	balkon (balcony)	3.82	1.00
astronaut (astronaut)	3.65	1.00	batterij (battery)	3.83	1.00
bakker (baker)	3.60	1.00	bladzijde (page)	3.63	1.00
chirurg (surgeon)	3.95	1.00	bushalte (busstop)	3.36	1.00
docent (teacher)	3.53	1.00	container (container)	3.59	0.99
fotograaf (photographer)	4.00	1.00	microfoon (microphone)	4.01	1.00
juwelier (jeweler)	3.31	1.00	orgaan (organ)	3.39	1.00
kapper (hair dresser)	4.07	1.00	pakket (package)	3.69	1.00
kelner (waiter)	3.38	0.99	regenwoud (rain forest)	3.24	1.00
leraar (teacher, practice)	4.47	0.99	ticket (ticket)	4.07	1.00
machinist (operator)	3.25	1.00	vanille (vanilla)	3.39	1.00
makelaar (estate agent)	3.71	1.00	zaklamp (flashlight)	3.71	1.00
matroos (sailor)	3.60	1.00			
monteur (mechanic)	3.70	1.00			
notaris (notary)	3.34	1.00			
postbode (mailman)	3.80	1.00			
redacteur (editor)	3.69	1.00			
schipper (skipper)	3.51	0.99			
slager (butcher)	3.82	1.00			
tandarts (dentist, practice)	4.16	1.00			

Note: ZipF = Zipf frequency.

Category: Vehicles

**Words**	**ZipF**	**Prevalence**	**Foils**	**ZipF**	**Prevalence**
helikopter (helicopter, practice)	4.34	0.98	oceaan (ocean, practice)	4.29	0.99
vrachtwagen (truck, practice)	4.24	1.00	schilderij (painting, practice)	4.33	1.00
brommer (moped)	3.03	1.00	badjas (bathrobe)	3.35	1.00
camper (camper)	3.57	0.99	blokhut (log cabin)	2.88	1.00
jeep (jeep)	3.89	0.98	borstel (brush)	3.32	1.00
kano (canoe)	3.35	0.99	contactlens (contact lens)	2.66	1.00
koets (carriage)	3.87	1.00	hamster (hamster)	3.28	1.00
limousine (limousine)	3.69	0.98	kachel (heater)	3.46	1.00
metro (subway)	4.12	1.00	kurk (cork)	3.21	1.00
motorfiets (motorbicycle)	3.28	1.00	lippenstift (lipstick)	3.81	1.00
politieauto (police car)	3.31	0.99	pijl (arrow)	3.85	1.00
roeiboot (rowing boat)	2.98	1.00	pruik (wig)	3.81	1.00
schoolbus (school bus)	3.22	1.00	roman (novel)	4.04	0.99
scooter (scooter)	3.69	0.99	stoplicht (traffic light)	3.21	1.00
sloep (sloop)	3.3	1.00			
sneltrein (express train)	2.74	1.00			
speedboot (speedboat)	2.95	1.00			
tandem (tandem)	2.2	0.99			
tractor (tractor)	3.38	1.00			
tram (tram)	3.26	1.00			
veerpont (ferry)	2.15	0.99			
vrachtschip (cargo ship)	3.4	1.00			

Note: ZipF = Zipf frequency.

Linguistic processing skills: Sentence production

29. Phrase and sentence generation

Phrase generation

**Block 1 (single words)**	**ZipF (single words)**	**Prevalence (single words)**	**Block 2 (two words)**	**Block 3 (adj/number+object)**	**Block 4 (adj+number+object)**
bus (bus) (practice)	4.81	1.00	bus en klok (bus and clock)	twee katten (two cats)	twee blauwe katten (two blue cats)
kat (cat) (practice)	4.72	1.00	kat en touw (cat and rope)	blauw touw (blue rope)	drie gele touwen (three yellow ropes)
klok (clock) (practice)	4.38	1.00			
touw (rope) (practice)	4.42	1.00			
blad (leaf)	4.05	1.00	blad en riem (leaf and belt)	blauw boek (blue book)	drie blauwe borden (three blue plates)
boek (book)	5.18	1.00	boek en hond (book and dog)	blauw slot (blue rope)	drie blauwe paarden (three blue horses)
bord (plate)	4.44	1.00	bord en slang (plate and snake)	blauwe neus (blue nose)	drie gele bladeren (three yellow leaves)
dak (roof)	4.74	1.00	dak en bord (roof and dog)	drie paarden (three horses)	drie gele daken (three yellow roofs)
hond (dog)	5.23	1.00	hond en muis (dog and mouse)	drie pennen (three pens)	drie gele muizen (three yellow mice)
muis (mouse)	4.05	1.00	muis en paard (mouse and horse)	drie slangen (three snakes)	drie gele neuzen (three yellow noses)
neus (nose)	4.85	1.00	neus en dak (nose and roof)	geel bord (yellow plate)	twee blauwe slangen (two blue snakes)
paard (horse)	4.92	1.00	paard en boek (horse and book)	geel dak (yellow roof)	twee blauwe boeken (two blue books)
pen (pen)	4.34	1.00	pen en slot (pen and lock)	gele muis (yellow mouse)	twee blauwe honden (two blue dogs)
riem (belt)	4.15	1.00	riem en pen (belt and pen)	twee bladeren (two leaves)	twee blauwe pennen (two blue pens)
slang (snake)	4.33	1.00	slot en blad (lock and leaf)	twee honden (two dogs)	twee gele riemen (two yellow belts)
slot (lock)	4.72	0.99	slang en neus (snake and nose)	twee riemen (two belts)	twee gele sloten (two yellow locks)

Note: ZipF = Zipf frequency.

Sentence generation

Verbs used (blocks 5-7)	ZipF	Prevalence
tekenen (draw) (practice)	4.68	0.9989
vinden (find) (practice)	5.78	0.9978
betalen (pay)	5.21	0.9964
fotograferen (photograph)	3.67	0.9991
interviewen (interview)	3.72	0.9921
kammen (comb)	3.42	0.9972
masseren (massage)	3.50	0.9966
meten (measure)	3.78	0.9989
achtervolgen (chase)	4.04	0.9968
helpen (help)	5.76	0.9988
voeren (feed)	4.56	0.9995
kussen (kiss)	4.66	0.9995
troosten (comfort)	3.66	0.9979
vervoeren (transport)	3.84	0.998
knuffelen (hug)	3.77	0.9964
slepen (drag)	3.95	0.997
stoppen (stop)	5.29	0.998
bedienen (serve)	3.85	0.9957
begroeten (greet)	3.74	0.9991
verzorgen (aiding)	4.03	0.9973

**Block 5**<br>**(questions)**	**Block 6**<br>**(active/passive)**	**Block 7**<br>**(conjunctive)**
tekent de vrouw de man?<br>(does the woman draw the man?)	de vrouw tekent de man<br>(the woman draws the man)	de zoektocht is ten einde, **omdat de man de vrouw vindt**<br>(the search is to an end, **because the man finds the woman**)
vindt de man de vrouw?<br>(does the man find the woman?)	de vrouw wordt door de man gevonden<br>(the woman is being found by the man)	de cursus moet al begonnen zijn, **want de vrouw tekent de man**<br>(the class must have started, **since the woman is drawing the man**)
betaalt de vrouw de man?<br>(does the woman pay the man?)	de man achtervolgt de vrouw<br>(the man chases the woman)	de automobilist moet wachten, **omdat de man de vrouw helpt**<br>(the motorist has to wait, **because the man is helping the woman**)
fotografeert de man de vrouw?<br>(does the woman photograph the man?)	de man helpt de vrouw<br>(the man helps the woman)	de EHBO-les lijkt in volle gang, **omdat de man de vrouw verzorgt**<br>(the first aid class seems well underway, **because the man is aiding the woman**)
interviewt de man de vrouw?<br>(does the man interview the woman?)	de man voert de vrouw<br>(the man feeds the woman)	de wandelaar kijkt raar op, **omdat de man de vrouw achtervolgt**<br>(the walker looks surprised, **because the man is chasing the woman**)
kamt de vrouw de man?<br>(does the woman comb the man?)	de vrouw kust de man<br>(the woman kisses the man)	er moet sprake zijn van een gevaarlijke situatie, **omdat de vrouw de man sleept**<br>(there must be a dangerous situation, **because the woman is dragging the man**)
masseert de man de vrouw?<br>(does the man massage the woman?)	de vrouw troost de man<br>(the woman comforts the man)	het publiek juicht, **omdat de vrouw de man kust**<br>(the audience is cheering, **because the woman is kissing the man**)
meet de vrouw de man?<br>(does the woman measure the man?)	de vrouw vervoert de man<br>(the woman transports the man)	iedereen weet dat de meeting begint, **omdat de man de vrouw begroet**<br>(everybody knows the meeting starts, **because the man is greeting the woman**)
	de man wordt door de vrouw geknuffeld<br>(the man is being hugged by the woman)	de wandelvierdaagse was vast zwaar, **want de vrouw vervoert de man**<br>(the four day walk must have been tough, **since the woman transports the man**)
	de man wordt door de vrouw gesleept<br>(the man is being dragged by the woman)	er moet iets naars gebeurd zijn, **want d**e **vrouw troost de man**<br>(something awful must have happened, **since the woman is comforting the man**)
	de man wordt door de vrouw gestopt<br>(the man is being stopped by the woman)	hardlopen is waarschijnlijk niet toegestaan hier, **want de vrouw stopt de man**<br>(it is probably not allowed to run here, **since the woman transports the man**)
	de vrouw wordt door de man bediend<br>(the woman is being served by the man)	het is tijd voor een drankje, **want de man bedient de vrouw**<br>(it is time for a drink, **since the man serves the woman**)
	de vrouw wordt door de man begroet<br>(the woman is being greeted by the man)	hun relatie moet goed zijn, **want de vrouw knuffelt de man**<br>(their relationship must be good, **since the woman hugs the man**)
	de vrouw wordt door de man verzorgd<br>(the woman is being aided by the man)	zij zullen wel geliefden zijn, **want de man voert de vrouw**<br>(they must be lovers, **since the man is feeding the woman**)

30. Spontaneous speech

Question 1: “Wat heeft u het afgelopen weekend gedaan?”

“What did you do during the last weekend?”

Question 2: “Vertel de verhaallijn van een film of serie die u recentelijk hebt gezien of een boek dat u hebt gelezen.”

“Tell the storyline of a movie or series that you have recently seen or a book that you have read.”

Question 3: “Vertel hoe uw perfecte vakantie eruit zou zien.”

“What would be your dream holiday?”

Linguistic processing skills: Sentence comprehension

31. Gender cue activation during sentence comprehension

Item	Gender	ZipF	Prevalence
fles (bottle, practice)	de	4.66	1
haai (shark, practice)	de	3.97	1
slot (lock, practice)	het	4.72	0.99
wiel (wheel, practice)	het	3.85	1
appel (apple)	de	4.01	1
auto (car)	de	5.66	1
bal (ball)	de	4.91	1
bever (beaver)	de	3.47	1
bezem (broom)	de	3.58	1
boom (tree)	de	4.72	1
doos (box)	de	4.58	1
fiets (bicycle)	de	4.34	0.99
fontein (fountain)	de	3.6	1
geit (goat)	de	3.91	0.99
gieter (watering can)	de	2.51	1
glijbaan (slide)	de	2.7	1
handschoen (glove)	de	3.82	1
hoed (hat)	de	4.56	1
kaas (cheese)	de	4.36	1
koelkast (refrigerator)	de	4.17	1
krokodil (crocodile)	de	3.69	1
muis (mouse)	de	4.05	1
neus (nose)	de	4.85	1
piano (piano)	de	4.15	1
pijl (arrow)	de	3.85	0.99
pinguïn (penguin)	de	3.39	-
piramide (pyramid)	de	3.44	0.98
radio (radio)	de	4.77	1
ring (ring)	de	4.72	1
rits (zipper)	de	3.64	1
roos (rose)	de	4.07	1
rugzak (backpack)	de	3.88	1
slak (snail)	de	3.38	1
sneeuwpop (snowman)	de	3.03	1
spijker (nail)	de	3.55	1
spin (spider)	de	3.89	1
stoel (chair)	de	4.71	1
tak (branch)	de	3.93	1
tandenborstel (toothbrush)	de	3.62	1
trompet (trumpet)	de	3.43	1
vleugel (wing)	de	3.95	0.99

vork (fork)	de	3.72	1
weegschaal (scale)	de	3.26	1
zebra (zebra)	De	3.49	1
anker (anchor)	het	3.7	1
aquarium (fish tank)	het	3.46	1
balkon (balcony)	het	3.82	1
been (leg)	het	4.73	0.99
blik (can)	het	4.56	1
boek (book)	het	5.18	1
bord (plate)	het	4.44	1
bot (bone)	het	4.16	1
brood (bread)	het	4.53	1
cadeau (gift)	het	4.47	1
gewei (antlers)	het	3.08	0.99
glas (glass)	het	4.76	1
graf (grave)	het	4.54	1
hart (heart)	het	5.29	1
hek (fence)	het	4.36	1
hert (deer)	het	3.79	1
hoefijzer (horseshoe)	het	2.85	1
horloge (watch)	het	4.45	1
kanon (cannon)	het	3.79	1
kasteel (castle)	het	4.44	1
konijn (rabbit)	het	4.28	1
kruis (cross)	het	4.32	1
masker (mask)	het	4.28	1
mes (knife)	het	4.67	1
nest (nest)	het	4.05	1
nijlpaard (hippo)	het	3.22	1
oog (eye)	het	4.84	1
oor (ear)	het	4.4	1
paard (horse)	het	4.92	1
palet (palette)	het	2.36	0.98
potlood (pencil)	het	3.74	1
raam (window)	het	4.85	1
schaap (sheep)	het	3.82	1
spaarvarken (piggy bank)	het	2.66	1
strijkijzer (iron)	het	2.85	1
stuur (steering wheel)	het	5.06	1
touw (rope)	het	4.42	1
varken (pig)	het	4.39	1
vliegtuig (airplane)	het	4.95	1
zadel (saddle)	het	3.84	1

Note: ZipF = Zipf frequency.

32. Verb semantics activation during sentence comprehension

Target object	Verb	Distractor	ZipF (target)	Prevalence (target)	CP
**Predictable items**					
bril (glasses, practice)	schilderen (paint)	slang (snake)	4.39	1	0
standbeeld (statue, practice)	onthullen (reveal)	zaklamp (flashlight)	3.67	1	0.17
baard (beard)	scheren (shave)	trui (sweater)	4.07	1	0.51
band (tire)	verwisselen (switch)	krant (paper)	4.9	1	0.46
bank (couch)	bekleden (upholster)	schip (ship)	4.96	1	0.14
beker (cup)	winnen (win)	kikker (frog)	3.94	1	0.06
biertje (beer)	drinken (drink)	koffer (suitcase)	4.54	1	0.6
bloem (flower)	planten (plant)	spuit (syringe)	4.13	1	0.06
boterham (sandwich)	smeren (prepare sandwich)	lucifer (lucifer)	3.78	1	0.77
broek (pants	passen (fit)	voet (foot)	4.83	1	0.49
contract (contract)	ondertekenen (sign)	vogel (bird)	4.55	1	0.69
deur (door)	openen (open)	pak (suit)	5.39	1	0.31
dief (thief)	arresteren (arrest)	aap (monkey)	4.48	1	0.34
ijsje (ice cream)	likken (lick)	emmer (bucket)	3.87	1	0.77
lamp (lamp)	vervangen (replace)	schop (shovel)	4.14	0.99	0.31
muur (wall)	behangen (paper)	trein (train)	4.83	1	0.8
sinaasappel (orange)	persen (press)	typemachine (typing machine)	3.25	0.98	0.71
taart (cake)	bakken (bake)	berg (mountain)	4.5	1	0.51
tafel (table)	dekken (cover)	sleutel (key)	4.92	1	0.66
tas (bag)	dragen (carry)	bus (bus)	4.77	1	0.09
vis (fish)	vangen (catch)	tent (tent)	4.7	1	0.23
wond (wound)	hechten (suture)	vlag (flag)	4.15	1	0.8
**Unpredictable items**					
ballon (balloon)	verbergen (hide)	laptop (laptop)	3.72	1	0
bijl (axe)	lenen (borrow)	pan (pan)	3.97	1	0
fluit (flute)	zoeken (search)	schaar (scissors)	3.87	1	0
frisbee (frisbee)	betalen (pay)	trommel (drum)	3.26	0.99	0
gitaar (guitar)	bewaken (guard)	robot (robot)	4.06	1	0
hanger (hanger)	pakken (get)	muffin (muffin)	3.26	0.98	0
kind (child)	zien (see)	bel (bell)	5.52	1	0
magneet (magnet)	tekenen (draw)	stoplicht (traffic light)	3.2	1	0
muts (cap)	verstoppen (hide)	zaag (saw)	3.65	1	0
overhemd (shirt)	verkopen (sell)	lippenstift (lipstick)	3.83	0.99	0
pen (pen)	stelen (steal)	klok (clock)	4.34	1	0
pijp (pipe)	verliezen (lose)	ei (egg)	4.14	0.99	0
sigaar (cigar)	filmen (film)	viool (violin)	3.99	1	0
sjaal (scarf)	ontvangen (receive)	kaars (candle)	3.73	0.99	0
spiegel (mirror)	kopen (buy)	pizza (pizza)	4.44	1	0
stethoscoop (stethoscope)	bekijken (watch)	paddestoel (mushroom)	2.7	0.97	0
uil (owl)	beschrijven (describe)	boor (drill)	3.5	1	0
veer (feather)	overhandigen (hand over)	mand (basket)	3.54	1	0
zeilboot (sailboat)	kiezen (choose)	colbert (jacket)	3.14	1	0
zwaard (sword)	fotograferen (photograph)	jurk (dress)	4.57	1	0

Note: ZipF = Zipf frequency; CP = Cloze probability.

33. Monitoring in noise in sentences
(green = target present; red = target absent)

Predictable Items

**Condition**	**Cue**	**ZipF**	**Prevalence**	**CP**	**Sentence**
cue-present (practice)	kroon (crown)	4.16	1.00	0.87	Op zijn hoofd droeg de koning een kroon en in zijn hand hield hij een scepter (On his head the king wore a crown and in his hand he held a mace)
cue-present (practice)	veer (feather)	3.54	1.00	0.00	Je vergeet het maar waarschijnlijk heb ik een veer van die zeldzame vogel (You might have forgotten, but I probably have a feather from that rare bird)
cue-absent (practice)	plein (plaza)	3.74	1.00	0.87	De valentijnskaart had de vorm van een hart en was rood (The valentine's day card was shaped like a heart and red)
cue-present	berg (mountain)	4.54	1.00	1.00	De klimmers bereikten het hoogste punt van de berg met een euforisch gevoel (The climbers reached the highest point of the mountain with a feeling of euphoria)
cue-present	deur (door)	5.39	1.00	1.00	Je moet altijd kloppen op mijn deur voordat je binnenkomt (You always have to knock on my door before entering)
cue-present	kaars (candle)	3.77	1.00	1.00	De stroom viel uit maar gelukkig had ik thuis nog een kaars om aan te steken (The power went out but luckily I had a candle at home to lit)
cue-present	boek (book)	5.18	1.00	1.00	De schrijfster ondertekende haar nieuwe boek voor haar trouwe fans (The writer signed her new book for her loyal fans)
cue-present	bord (plate)	4.44	1.00	1.00	Ik schep meestal teveel op mijn bord als mijn moeder kookt (I usually put too much food on my plate when my mother cooks)
cue-present	bril (glasses)	4.39	1.00	1.00	Mijn opa kan niets lezen zonder zijn bril met multifocale glazen (My grandfather cannot read anything without his glasses with customized progressive lenses)
cue-present	helm (helmet)	4.04	1.00	1.00	De jongen reed op een scooter zonder helm en kreeg een bekeuring (The boy was riding his scooter without a helmet and got fined)
cue-present	hond (dog)	5.23	1.00	1.00	De puppy werd een grote hond met veel haar (The puppy became a big dog with a lot of hair)
cue-present	mand (basket)	3.63	1.00	1.00	Voor de picknick pakken we alles in een mand van riet (For the picnic, we gather everything in a basket made of cane)

cue-present	nest<br>(nest)	4.05	1.00	1.00	De jonge vogeltjes leefden nog steeds in hun nest gemaakt van takjes en bladeren<br>(The little birds still lived in their nest made of twigs and leaves)
cue-present	pijl<br>(arrow)	3.85	0.99	1.00	De boogschutter schoot met een puntige pijl op het doel<br>(The archer shot a pointy arrow towards the goal)
cue-present	clown<br>(clown)	4.07	1.00	1.00	In dit circus hebben ze een heel grappige clown die iedereen aan het lachen maakt<br>(In this circus they have a very funny clown who makes everybody laugh)
cue-present	kaart<br>(map)	4.90	1.00	1.00	De wereldatlas bevat van ieder land een kaart met legenda<br>(The world atlas contains for each country a map with a legend)
cue-present	schoen<br>(shoe)	4.13	0.99	1.00	Voor Sinterklaas zetten de kinderen hun schoen bij de open haard<br>(For Sinterklaas all the children put out their shoe at the fireplace)
cue-present	zweep<br>(whip)	3.69	1.00	1.00	Het paard wordt aangespoord met een zweep door de strenge ruiter<br>(The horse is urged with a whip by the stern rider)
cue-present	fluit<br>(whistle)	3.87	1.00	1.00	De scheidsrechter blies hard op zijn fluit toen er een overtreding werd gemaakt<br>(The referee loudly blew his whistle when a violation was made)
cue-present	maan<br>(moon)	4.62	1.00	1.00	De astronauten landden op de maan op 20 juli 1969<br>(The astronauts landed on the moon on 20 July 1969)
cue-present	bij<br>(bee)		1.00	1.00	Honing wordt gemaakt door een bij die nectar uit bloemen verzamelt<br>(Honey is made by a bee that collects nectar from flowers)
cue-present	graf<br>(grave)	4.54	1.00	0.93	Op het kerkhof bezocht ze haar vaders graf en legde er bloemen<br>(At the graveyard she visited her father's grave and put flowers on it)
cue-present	bank<br>(couch)	4.96	1.00	1.00	Op zondagen hang ik vaak lekker op de bank met een zak chips<br>(On Sundays I am often relaxing on the couch with a bag of crisps)
cue-absent	pan<br>(pan)	3.97	1.00	1.00	De gelovigen gaan met kerstavond altijd naar de kerk in het dorp<br>(On Christmas eve, people who believe in God always go to church in the village)
cue-absent	vork<br>(fork)	3.72	1.00	1.00	Om haar vinger droeg ze een mooie ring van goud<br>(On her ring she wore a beautiful ring made of gold)

cue-absent	ruit (diamonds)	3.57	1.00	1.00	Ik doe deze bloemen in een vaas met een laag schoon water (I put these flowers in a vase filled with clean water)
cue-absent	vos (fox)	3.88	1.00	1.00	Jonge meisjes spelen graag met een pop of een barbie (Young girls like to play with a doll or a Barbie doll)
cue-absent	knop (button)	4.22	1.00	1.00	Mijn nichtje is jarig dus ik bak een taart met veel chocolade (It is my cousin's birthday so I am baking a cake with a lot of chocolate)
cue-absent	lijm (glue)	3.60	1.00	1.00	Pinocchio was een jongen met een lange neus omdat hij steeds loog (Pinocchio was a boy with a long nose because he always lied)
cue-absent	plank (shelf)	4.05	1.00	1.00	Zijn broek zakt af want hij heeft geen riem omgedaan (His pants are dropping because he did not put on a belt)
cue-absent	sneeuw (snow)	4.39	1.00	1.00	Bob was moe dus ging hij naar bed zodra zijn vriendin thuiskwam (Bob was tired so he went to bed as soon as his girlfriend got home)
cue-absent	haak (hook)	4.10	1.00	1.00	De Utrechtse Dom luidt heel hard haar klok die gerenoveerd was (The Dom in Utrecht very loudly rings her bell which was renovated)
cue-absent	noot (nut)	3.58	1.00	1.00	Deze twee planken moet je vastzetten met een schroef in de muur (These two shelves should be secured with screws onto the wall)

Note: ZipF = Zipf frequency; CP = Cloze probability.

Control Items

Condition	Cue	ZipF	Prevalence	CP	Sentence
cue-present	bal (ball)	4.91	1.00	0	We proberen het eerst met een bal die niet zo zwaar is (We will first try with a ball that is not so heavy)
cue-present	kan (can)		1.00	0	De winkel verkocht een rode kan voor tien euro (The store sold a red can for ten euros)
cue-present	koe (cow)	4.27	1.00	0	Het kind tekende een koe op het schoolbord (The child drew a cow on the blackboard)
cue-present	mug (mosquito)	3.35	0.99	0	In de ruimte was een irritante mug die rond mijn hoofd zoemde (In the room there was an annoying mosquito buzzing around my head)
cue-present	net (net)		1.00	0	Ze wilden iets anders dus ze kochten een net om vissen te vangen (They wanted to do something else so they bought a net to catch fish)
cue-present	put (well)	4.07	0.99	0	Vroeger moesten mensen lang lopen naar een put om aan water te komen (Back in the days people walked a long time to a well to get water from)
cue-present	tas (bag)	4.77	1.00	0	Het tijdschrift adverteerde met een dure tas van een Frans merk (The magazine advertised with an expensive bag of a French brand)
cue-present	zon (sun)	4.84	1.00	0	Dit is een plaats met veel zon in de zomer (This is a place with a lot of sun in the summer)
cue-present	doos (box)	4.58	1.00	0	Toen ik in de hoek keek, zag ik een doos waarin een poes lag te slapen (When I looked in the corner, I saw a box with a cat that was sleeping)
cue-present	jurk (dress)	4.75	1.00	0	Het meisje maakte een lange jurk van paars zijde (The girl made a long dress of purple silk)
cue-present	lamp (lamp)	4.14	0.99	0	Voor het bezoek van mijn oom kocht ik een lamp bij de IKEA (For the visit to my uncle I bought a lamp at IKEA)
cue-present	tent (tent)	4.61	1.00	0	Gedurende de dag was hij nauwelijks in de tent te vinden (During the day he was hardly ever in the tent)
cue-present	roos (rose)	4.07	1.00	0	De jongen kocht op de markt een gele roos voor zijn vriendin (At the market the boy bought a yellow rose for his girlfriend)
cue-present	slang (snake)	4.33	1.00	0	Die groep mensen is druk in gesprek over een slang die als huisdier werd gehouden (That group of people is busy discussing a snake which was held as a pet)
cue-present	schaar (scissors)	3.80	0.99	0	Ik moet dit kunnen aanpassen maar heb helaas geen schaar bij me (I need to be able to adjust this but I don’t have a pair of scissors with me)

cue-present	schelp (shell)	3.13	1.00	0	We zoeken op het werk naar een schelp om aan onze collectie toe te voegen (At work we are searching for a shell to add to our collection)
cue-present	bad (bath)	4.63	1.00	0	In de avond neem ik heel graag een bad voordat ik naar bed ga (At night I love to take a bath before going to bed)
cue-present	bot (bone)	4.16	1.00	0	Toen ik mijn eten at, zag ik daar een stukje bot in zitten (When eating my dinner, I noticed it had a piece of bone in it)
cue-present	pruik (wig)	3.81	1.00	0	De vrouw gebruikte steeds meer haar pruik als ze naar buiten ging (The woman more often used her wig when going outside)
cue-present	kurk (cork)	3.21	1.00	0	Ik maak dit nog dicht met een kurk en ga dan naar huis (I will close this with a cork and then I will go home)
cue-absent	staart (tail)	4.25	1.00	0	In de reclame zag je een bijl om hout mee te hakken (In the advertisement you saw an axe for wood chopping)
cue-absent	rijst (rice)	3.96	1.00	0	Ze kunnen hier niet lopen vanwege een onbekend probleem aan de brug dat nog niet verholpen is (They cannot walk here because of an unknown problem with the bridge that has not yet been fixed)
cue-absent	wijn (wine)	4.78	1.00	0	Ik las een verhaal over een kooi en een tijger (I read a story about a cage and a tiger)
cue-absent	vlag (flag)	4.25	1.00	0	De man genoot van het ruiken aan de pijp als hij thuis was (The man enjoyed smelling the pipe when he was home)
cue-absent	zwaard (sword)	4.57	1.00	0	Dit wordt gemaakt van brood dat een paar dagen oud is (This is made of bread that is a few days old)
cue-absent	boot (boat)	4.98	1.00	0	In het hoekje vond hij zijn knoop die hij verloren was (In the corner he found his button that he had lost)
cue-absent	troon (throne)	4.08	1.00	0	Het meisje maakte een sjaal voor de winter (The girl made a scarf for the winter)
cue-absent	rook (smoke)	4.67	0.99	0	De winkel op de hoek verkoopt een mooie pet van het nationale voetbalelftal (The shop on the corner sells a nice cap from the national soccer team)
cue-absent	trap (stairs)	4.72	1.00	0	Het is niet goed om iets te halen uit een blik dat ongeopend bol staat (It is not good for you to take something from a can that is unopened and bunchy)
cue-absent	sok (sock)	3.49	1.00	0	Voor de jonge vrouw pakken we de kam om haar haar in model te brengen (For the young woman we get the comb to style her hair)

Note: ZipF = Zipf frequency; CP = Cloze probability.
